# β-Ketoamides in Thieno[2,3-*b*]pyridine Series: Synthesis, Quantum Chemical Studies and 2,4-D Herbicide Safening Effects

**DOI:** 10.3390/molecules31172975

**Published:** 2026-08-25

**Authors:** Artem A. Amirkhanyan, Victor V. Dotsenko, Alexandr Bespalov, Tatyana R. Hagur, Eva S. Daus, Igor V. Yudaev, Yuliia V. Daus, Darya Yu. Lukina, Vladimir K. Vasilin, Nicolai A. Aksenov, Inna V. Aksenova

**Affiliations:** 1 Department of Organic Chemistry and Technologies, Kuban State University, 149 Stavropolskaya St., 350040 Krasnodar, Russia; 2 Department of Chemistry, North Caucasus Federal University, 1a Pushkin St., 355017 Stavropol, Russia; 3 Faculty of Energetics, Kuban State Agrarian University, 13 Kalinina St., 350044 Krasnodar, Russia; 4 Department of Biotechnology and Animal Product Technology, Kuban State Technological University, 2 Moskovskaya St., 350072 Krasnodar, Russia

**Keywords:** thieno[2,3-b]pyridines, 4,5,6,7-tetrahydrothieno[2,3-b]pyridines, β-ketoamides, Thorpe-Ziegler cyclization, thieno[2,3*-b*;4,5*-b*]dipyridines, cyanothioacetamide, 2,4-D herbicide safeners, quantum chemical studies, X-ray diffraction analysis, agrochemicals

## Abstract

The aim of this work was to synthesize previously unknown thieno[2,3-b]pyridines bearing a β-ketoamide moiety, to study their structure, and to evaluate their agrochemical potential as 2,4-D herbicide safeners. A series of 3-(3-aminothieno[2,3-b]pyridine-2-yl)-3-oxo-*N*-phenylpropanamides were prepared via S-alkylation of readily available 2-thioxonicotinonitriles or 3-cyanopyridine-2-thiolates with γ-bromoacetoacetanilide, followed by base-promoted Thorpe–Ziegler cyclization. The structure of 4-[(3-cyano-4,6-dimethylpyridin-2-yl)thio]-3-oxo-*N*-phenylbutanamide was unambiguously established by single-crystal X-ray diffraction. Quantum chemical calculations (B3LYP-D3BJ/6-311+G(2d,p)) revealed that the ketone form is significantly more stable than the enol tautomer with a calculated equilibrium constant in DMSO of 1.52 × 10^−5^, which is consistent with the absence of enol form signals in the NMR spectra. The calculated IR frequencies, after scaling, showed excellent agreement with experimental data. Additionally, we found that 4-[(3-cyano-4,6-dimethylpyridin-2-yl)thio]-3-oxo-*N*-phenylbutanamide undergoes intramolecular cyclization upon heating in AcOH or DMF, to give 4-hydroxy-7,9-dimethylthieno [2,3*-b*:4,5*-b*′]dipyridin-2(1*H*)-one via aniline elimination, thus providing an alternative route to tricyclic thieno [2,3*-b*;4,5*-b*]dipyridine ensembles. Agrochemical screening demonstrated that some compounds exhibit pronounced antidote activity against the herbicide 2,4-dichlorophenoxyacetic acid (2,4-D) in sunflower seedlings, with root and hypocotyl protection reaching 119–156% in laboratory tests. Field trials for the three most active compounds confirmed their efficacy, providing crop yield increases of 46–57% relative to the herbicide-only control. These results indicate that the newly synthesized thienopyridine β-ketoamides represent a promising class of 2,4-D herbicide safeners.

## 1. Introduction

Thieno[2,3-b]pyridines are a privileged class of fused heterocyclic scaffolds that have attracted sustained interest in organic and medicinal chemistry owing to their unique structural features, synthetic versatility, and diverse biological profiles (for reviews see [1,2,3,4,5,6,7,8,9,10]). These bicyclic systems, combining an electron-rich thiophene ring with a nitrogen-containing pyridine moiety, exhibit enhanced reactivity at multiple sites, enabling a wide range of transformations such as oxidation [11,12] and further condensations (e.g., [13,14]), and thus affording compounds with valuable properties.

The chemistry of thieno[2,3-b]pyridines has been extensively reviewed over the decades. Early foundational works summarized synthetic methods and pharmacological properties [15,16]. Subsequent comprehensive overviews detailed their role in condensed heterocyclic systems [2,3,4,7,8,9]. More specialized reviews addressed agrochemical applications of thienopyridines [17]. The accessibility of 3-aminothieno[2,3-b]pyridine derivatives positions them as popular starting reagents for constructing more complex polycyclic architectures [18,19].

**Figure 1:**
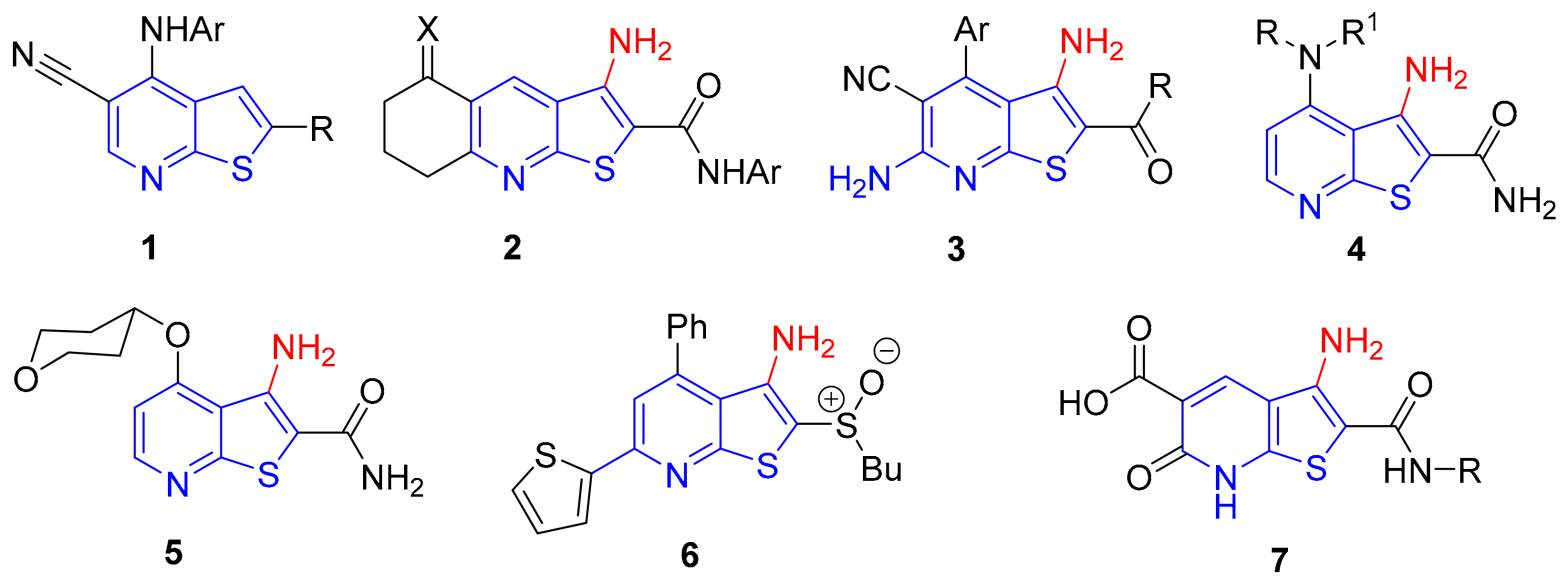
Biologically active 3-aminothieno[2,3-b]pyridines.

Beyond their synthetic utility, thieno[2,3-b]pyridines demonstrate substantial practical value as bioactive molecules, spanning pharmaceuticals and agrochemicals. Thus, 4-aminothienopyridine-3-carbonitriles **1** (Figure 1) have shown potent activity against *Staphylococcus epidermidis* [20], *Leishmania amazonensis* [21], and *Mayaro* virus [22], alongside their role as protein kinase Cθ (PKCθ) inhibitors for treating autoimmune and inflammatory conditions [23,24,25]. Easily available thieno[2,3-b]quinolines **2** [26] function as phosphoinositide-specific phospholipase C-γ (PLC-γ) enzyme inhibitors and G protein-coupled receptor (GPCR) modulators with significant antiproliferative activities against a range of human cancer cell lines [27,28,29,30,31]. Compound **2** also inhibits platelet aggregation, exhibiting synergy with aspirin, with some compounds demonstrating activity comparable to or exceeding that of clopidogrel, the primary antithrombotic agent of the thieno [3,2-*c*]pyridine class [32]. 3,6-Diaminothieno[2,3-b]pyridines **3** act as selective inhibitors of plasmodial glycogen synthase kinase-3 (PfGSK-3) [33,34,35,36], cytokine TGF-βR1 modulators [37], and bacterial histidine kinase autophosphorylation inhibitors [38]. They have also been reported as inhibitors of heat shock protein Hsp90 and serine/threonine kinase B-Raf [39], and inhibitors of infectious prion isoform PrP^Sc^ replication [40]. Bone anabolic agents **4** [41] as well as alkaline phosphatase enhancers **5** [42] promote osteogenesis in osteoporosis models (Figure 1).

Also, thienopyrid-2-yl sulfoxides **6** (Figure 1) were recognized as 15-prostaglandin dehydrogenase (15-PGDH) inhibitors and are effective for recovery from bone marrow transplantation, ulcerative colitis, and partial hepatectomy [43]. 3-Aminothienopyridine-5-carboxylic acids **7** serve as IKKβ inhibitors [44], UCH-L1 inhibitors [45], and HIV-1 integrase inhibitors [46].

In agrochemistry, thienopyridines recently were reported as insecticidal agents [47,48,49], herbicide safeners [50,51,52,53,54,55], plant growth stimulators [56] and fungicides [57].

The aim of this work is the synthesis and characterization of previously undescribed thieno[2,3-b]pyridines bearing a β-ketoamide moiety, as well as the evaluation of the agrochemical potential of the new compounds as herbicide safeners.

It should be noted that β-ketoamides are widely used in organic synthesis as valuable building blocks and active methylene compounds [58,59,60], for the preparation of pharmaceuticals, and as complexing agents [61,62]. As far as we know, β-ketoamides incorporating a thienopyridine fragment have not been described in the literature to date.

## 2. Results and Discussion

### 2.1. Synthesis

As starting reagents for the synthesis of the target products, we used readily available γ-bromoacetoacetanilide and a series of S-nucleophiles of the pyridine series.

**Scheme 1:**
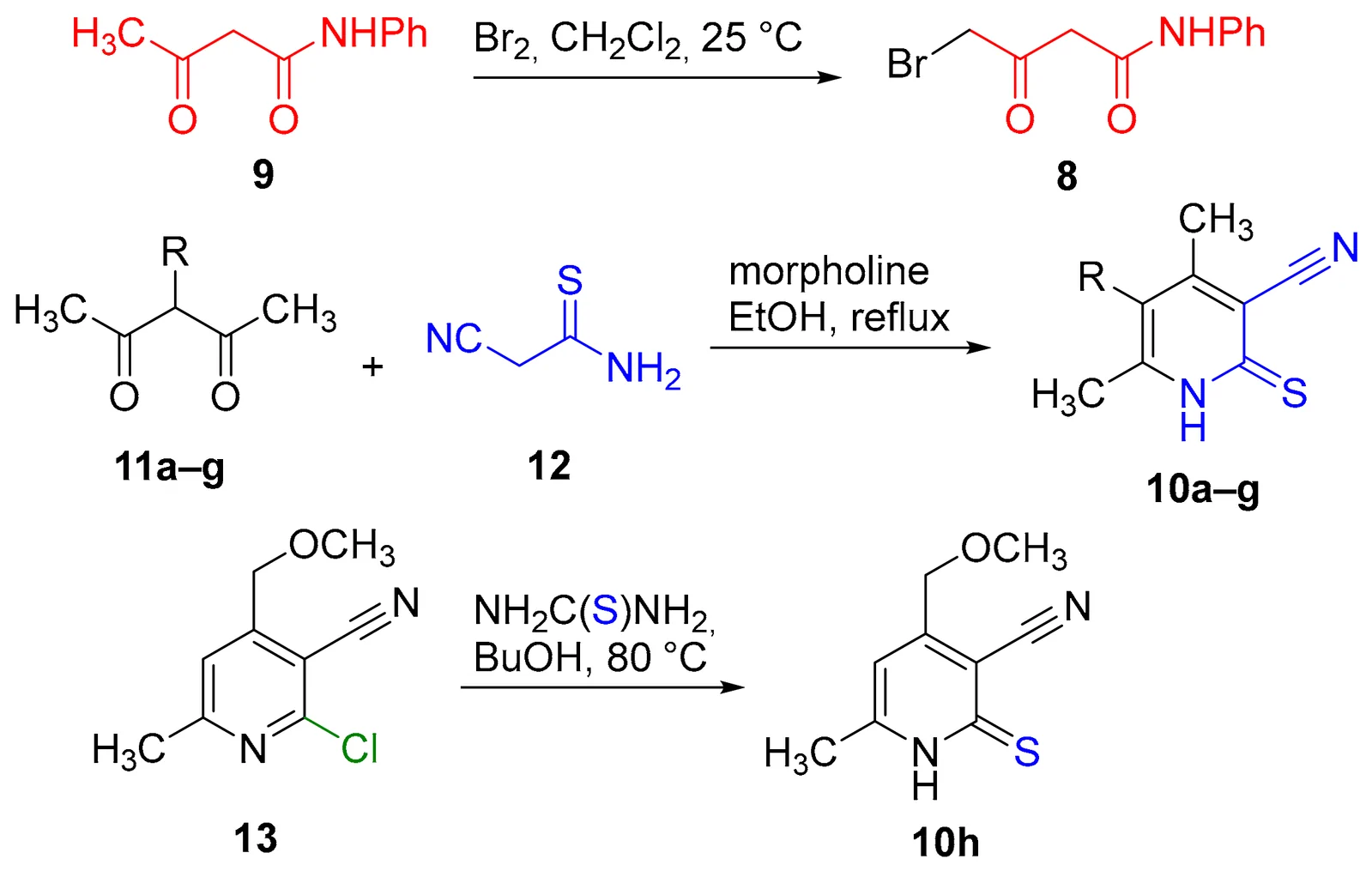
Synthesis of starting compounds **8** and **10a**–**h**. **10**, **11**: R = H (**a**), n-Bu (**b**), allyl (**c**), Et (**d**), Me (**e**), CH_2_C(O)Ph (**f**), CH_3_(CH_2_)_4_ (**g**).

Thus, γ-bromoacetoacetanilide **8** was prepared by direct bromination of acetoacetanilide **9** according to [63]. 2-Thioxonicotinonitriles **10a**–**g** were obtained by the reaction of 3-substituted pentane-2,4-diones **11a**–**g** with cyanothioacetamide **12** [64] in the presence of morpholine according to known procedures [56,65,66,67,68] (Scheme 1). Nicotinonitrile **10h** was prepared by heating 6-methyl-4-(methoxymethyl)-2-chloronicotinonitrile **13** with thiourea in butanol following a described procedure [69].

**Scheme 2:**
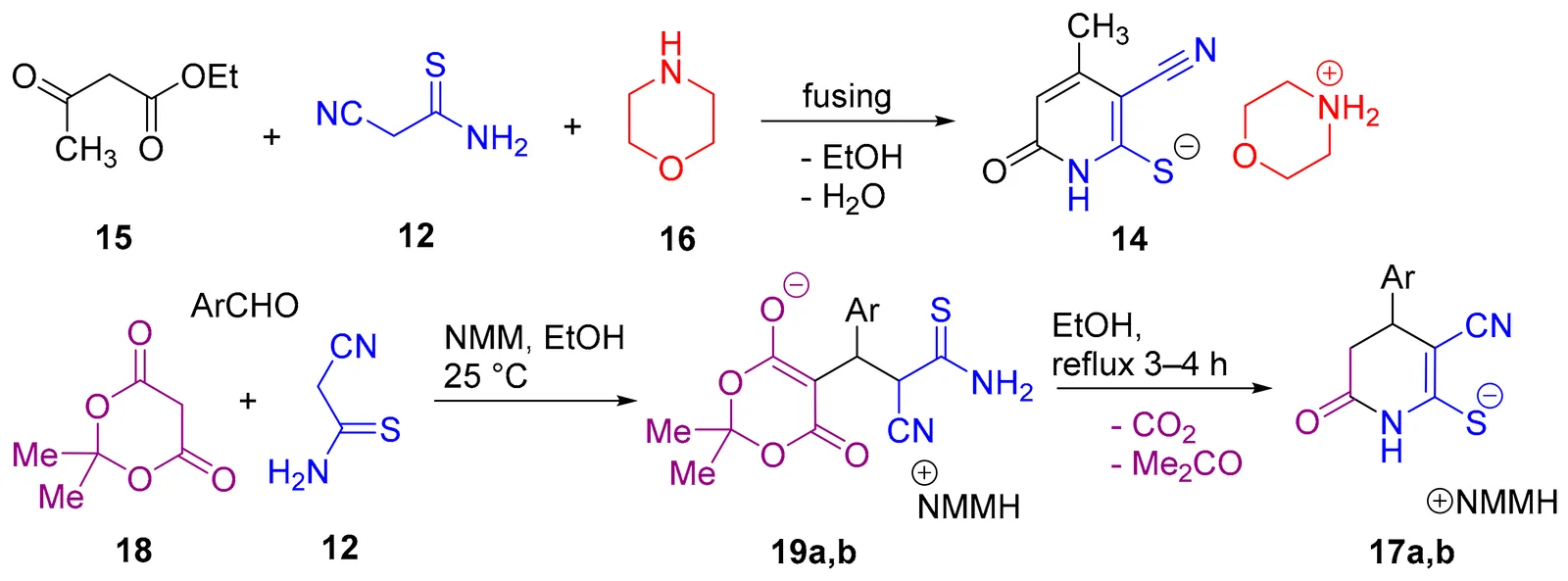
Synthesis of starting pyridine-2-thiolates **14** and **17a**,**b**. **17**, **19**: Ar = 2-ClC_6_H_4_ (**a**), 5-methylfur-2-yl (**b**). NMM = *N*-methylmorpholine.

Pyridine-2-thiolate **14** was obtained by fusion of ethyl acetoacetate **15**, cyanothioacetamide **12**, and morpholine **16** [70]. Another S-nucleophilic species, *N*-methylmorpholinium 1,4,5,6-tetrahydropyridine-2-thiolate **17a**,**b**, was prepared by the known method [71,72] starting from cyanothioacetamide **12**, aromatic aldehydes and Meldrum’s acid **18,** followed by intramolecular cyclization of the isolable Michael adducts **19a**,**b** (Scheme 2).

**Scheme 3:**
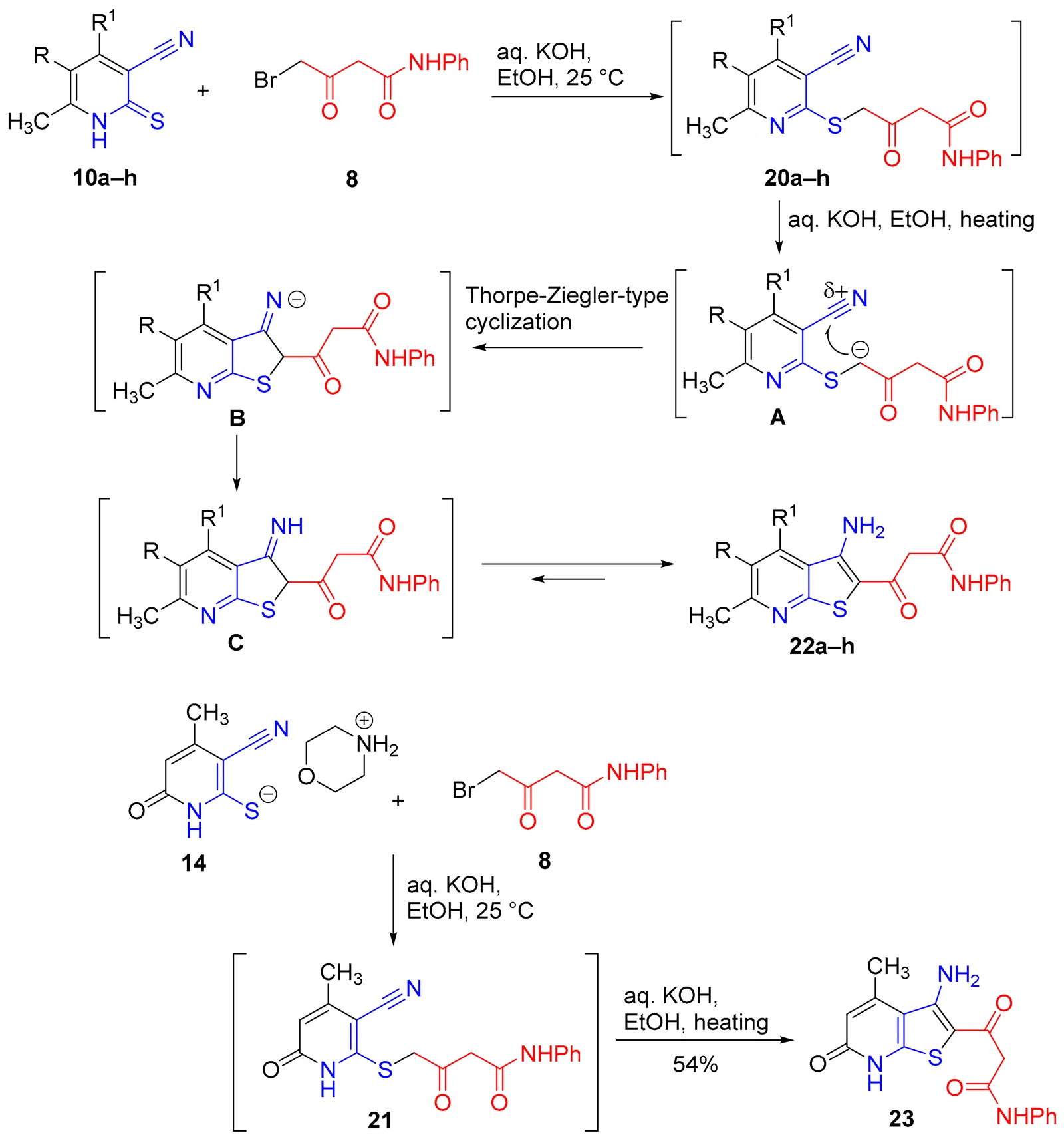
The reaction of S-nucleophiles **10** and **14** with γ-bromoacetoacetanilide **8**.

**Figure 2:**
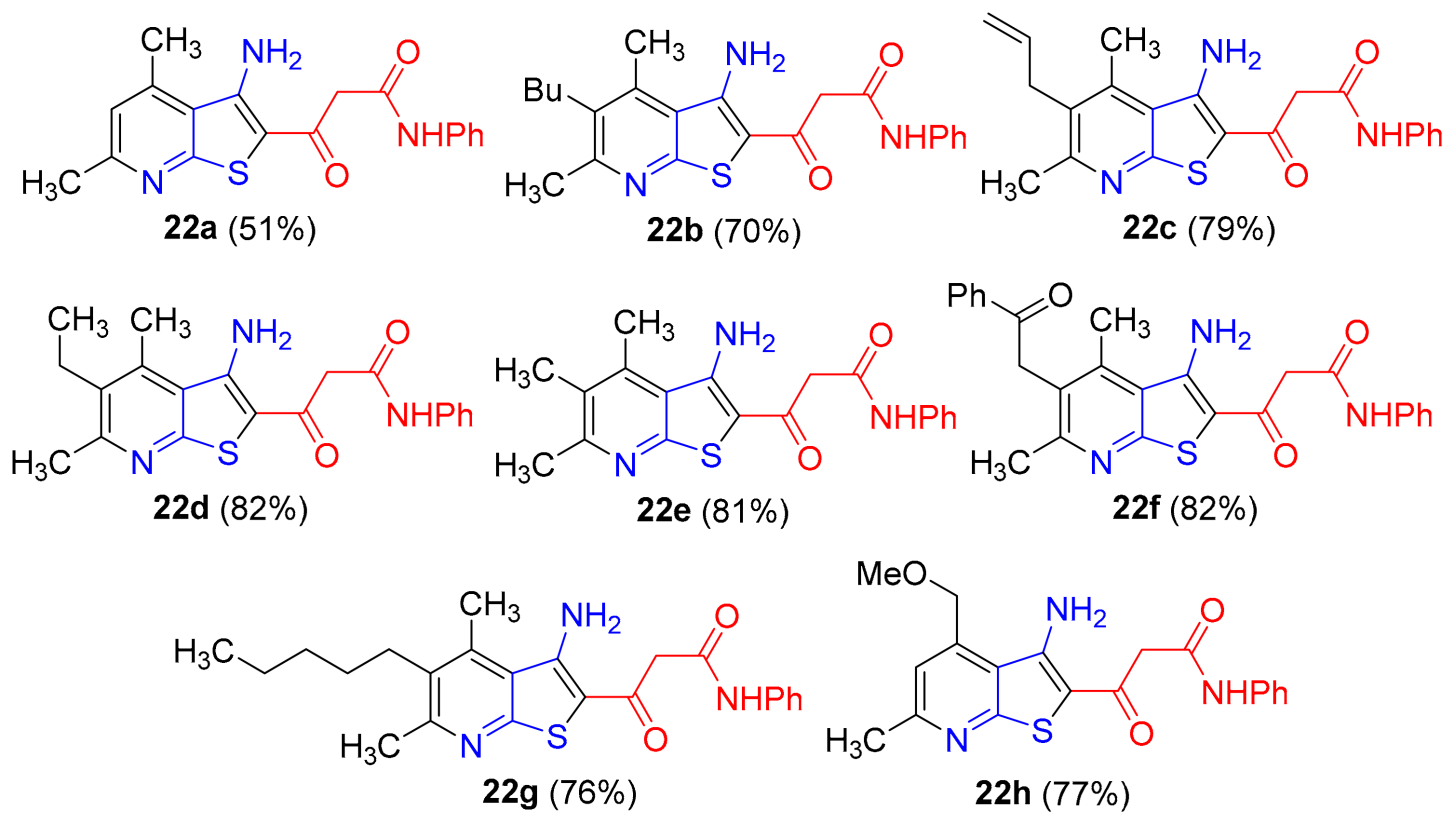
Structure and yields of compounds **22a**–**h**.

We found that the reaction of γ-bromoacetoacetanilide **8** with S-nucleophiles **10** and **14** proceeds via the formation of isolable S-alkylation products **20** and **21**, which upon further heating in the presence of a base very readily undergo Thorpe–Ziegler cyclization to afford 4-(thieno[2,3-b]pyridine-2-yl)-3-ketoamides **22** and **23** in good yields (Scheme 3). The structure of the intermediate was supported by the isolation and characterization of ketoamide **20d** (R = Et, R^1^ = Me). By analogy with the literature data [1,2,3,4,5], we propose that the cascade cyclization proceeds via the formation of carbanion **A**. Although the –SCH_2_C(O)– fragment is notably less acidic than the β-ketoamide residue –C(O)CH_2_C(O)NR, the generation of carbanion **A** drives subsequent cyclization to furnish a stable aromatic system, thereby ensuring a favorable shift in the equilibrium. Intramolecular attack by the carbanion at the nitrile carbon atom affords anion **B**. Being a strong base, the latter is converted into imine **C** in an alcoholic medium, which subsequently undergoes stabilization into the more stable aromatic thienopyridine form (Scheme 3). Figure 2 summarizes the structures and yields of the products obtained in this manner.

**Scheme 4:**
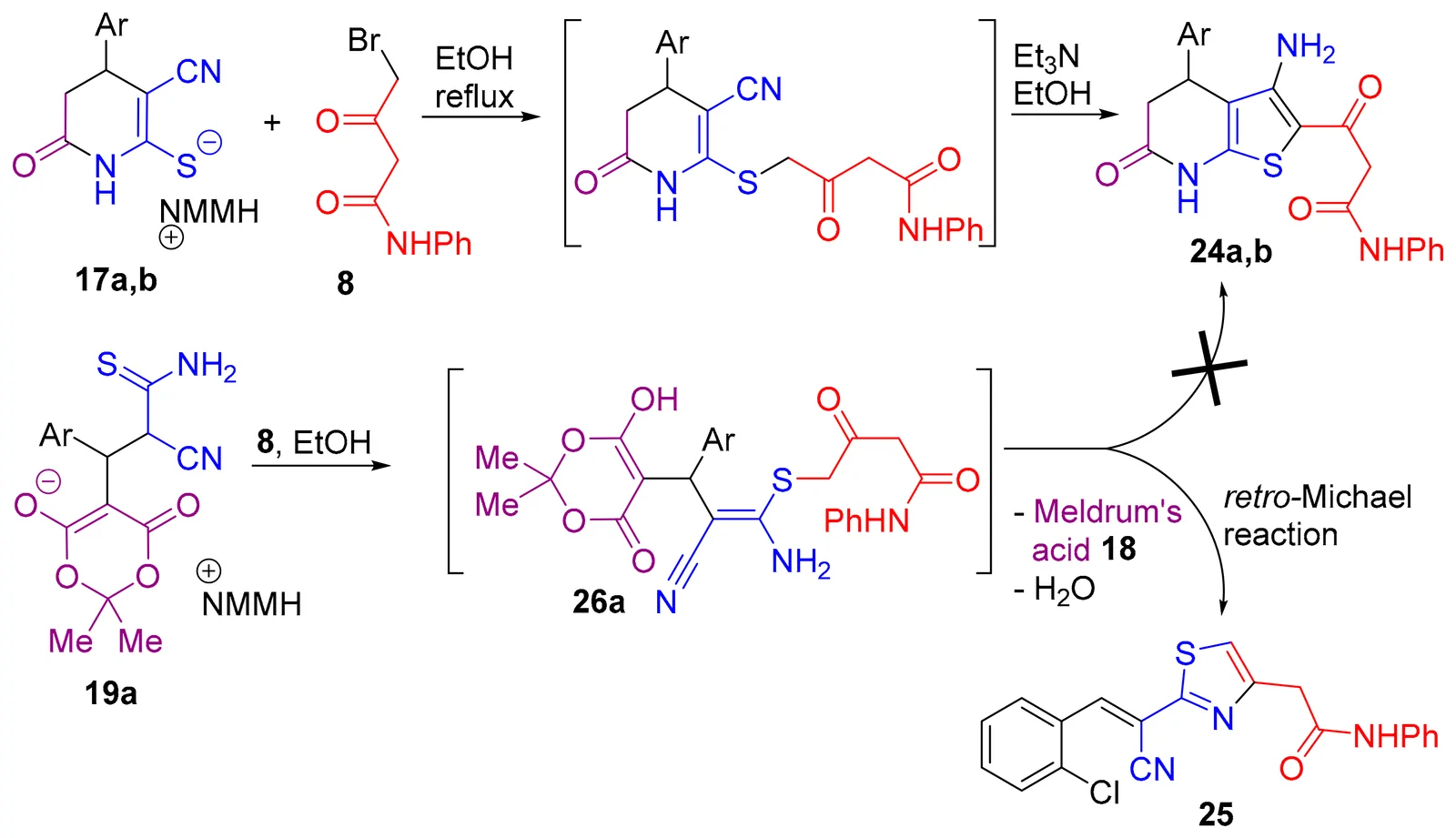
The reaction of S-nucleophiles **17a**,**b** and **19a** with γ-bromoacetoacetanilide **8**. **17**, **19**, **24**, **26**: Ar = 2-ClC_6_H_4_ (**a**), 5-methylfur-2-yl (**b**). NMM = *N*-methylmorpholine.

1,4,5,6-Tetrahydropyridine-2-thiolates **17a**,**b** react in a similar way. Thus, treatment with γ-bromoacetoacetanilide **8** upon heating in EtOH in the presence of Et_3_N afforded the corresponding β-ketoamides **24a**,**b**, which bear the relatively rare 4,5,6,7-tetrahydrothieno[2,3-b]pyridine moiety (Scheme 4). However, when the thiolate **17a** precursor, Michael adduct **19a**, was treated with γ-bromoacetoacetanilide **8**, the corresponding tetrahydrothienopyridine **24a** could not be obtained. Instead, the product of competitive cyclization, namely thiazole **25**, was isolated. We propose that the first step of the process involves S-alkylation to afford acyclic intermediate **26**. This intermediate then undergoes a *retro-*Michael fragmentation with elimination of Meldrum’s acid **18**, followed by thiazole ring closure via the Hantzsch-type cyclization.

**Figure 3:**
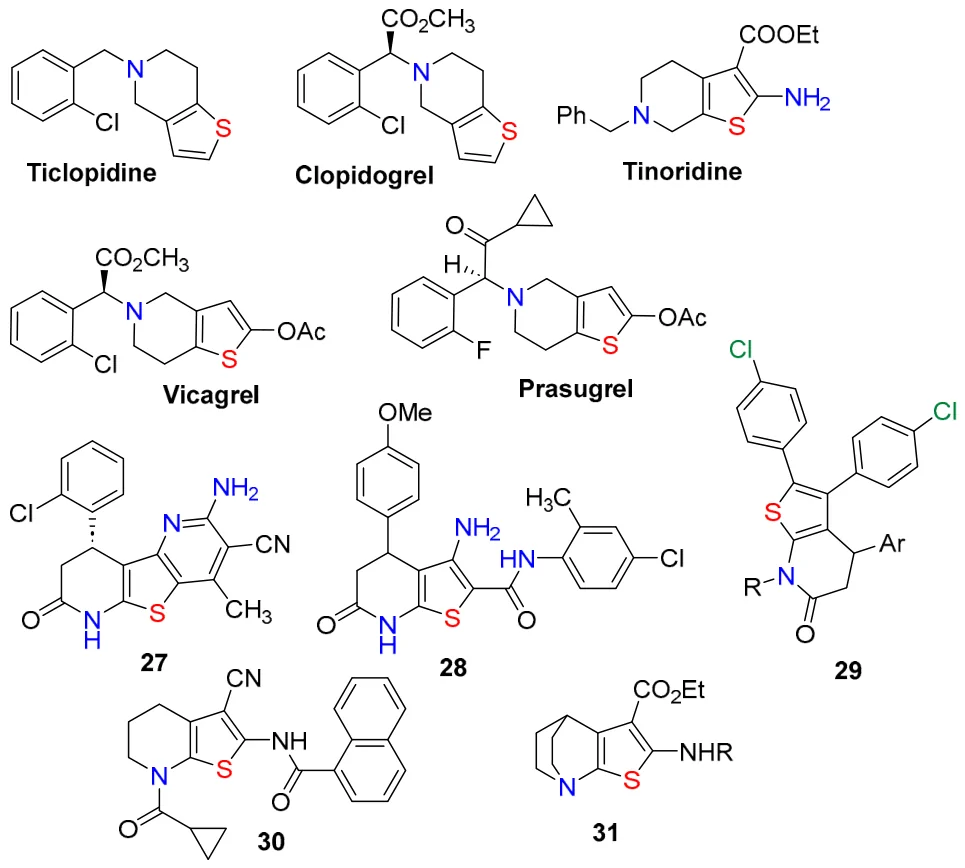
Biologically active tetrahydrothienopyridines.

Here it should be noted that many compounds featuring a 4,5,6,7-tetrahydro thienopyridine core are of considerable practical importance. Among derivatives of the [3,2-*c*]-isomer, attention should be paid to the group of selective irreversible P2Y_12_ ADP receptor inhibitors used to block platelet aggregation—namely, the well-known drugs Ticlopidine, Clopidogrel, Prasugrel, and Vicagrel (for key studies on the chemistry and applications of these tetrahydrothienopyridines, see [73,74,75,76,77,78]) (Figure 3). Among the [2,3-*c*]-isomer derivatives, Tinoridine, a non-steroidal anti-inflammatory agent known since the late 1970s, can be highlighted [79,80]. In contrast, the chemistry of [2,3-b]-isomer has received only limited attention [73,81,82,83,84,85,86,87]. This is probably due to the lack of convenient methods for constructing the 4,5,6,7-tetrahydrothieno[2,3-b]pyridine system, which in turn stems from the relative inaccessibility of tetrahydronicotinonitrile precursors. Nevertheless, the few available reports indicate that research in this area is promising. For example, dipyridothiophene **27** [88] (Figure 3) represents a selective non-steroidal progesterone receptor agonist. Thienopyridine **28** displays agrochemical potential and exhibits a herbicide safening effect against 2,4-D in sunflower seedling experiments [54]. Compound **29** inhibits the interaction between the tumor suppressor P53 and the oncoprotein MDM2 [89]. Thieno[2,3-b]pyridine **30** has been reported as a JNK2/JNK3 kinase inhibitor [90], whereas thienoquinuclidines **31** show antihistamine and anti-exudative activity [91].

Compounds **22**–**24** are beige or yellow powders, typically exhibiting poor solubility in benzene and alcohols, but soluble in acetone or (CD_3_)_2_SO at 25 °C. The IR spectra of β-ketoamides **22**–**24** display an intense absorption band corresponding to the stretching vibrations of the N–H bonds of the amide and amino groups in the region of ν 3491–3167 cm^−1^. Absorption bands attributable to the stretching vibrations of the CONHPh carbonyl groups are expectedly observed at ν 1651–1679 cm^−1^. At the same time, the absorption band of the ketone C=O group undergoes a noticeable bathochromic shift and appears as part of complex absorption bands in the range of 1585–1601 cm^−1^ (see Section 2.2, Quantum chemical studies).

Characteristic signals in the ^1^H NMR spectra of thienopyridine β-ketoamides **22**–**24** include a singlet for the methylene protons of the CO–CH_2_–CONH fragment in the range δ 3.53–3.79 ppm and a broad singlet for the NH_2_ protons at δ 7.36–7.87 ppm. Singlets for the amide N–H protons appeared at δ 10.13–10.28 ppm and three multiplets for the Ph protons expectedly appeared at δ 7.02–7.62 ppm. In addition, singlet resonances for the methyl groups attached to the thienopyridine core (C(4)CH_3_ and C(6)CH_3_) are observed at δ 2.17–2.71 ppm. For the tetrahydro thienopyridines **24a**,**b**, the CH_2_–CHAr protons give rise to a complex ABX-pattern consisting of two multiplets (or dd) for the diastereotopic C(5)H_2_ protons at δ 2.63–3.19 ppm and a multiplet for the C(4)H proton at δ 4.34–4.59 ppm.

In the ^13^C NMR spectra of β-ketoamides **22**–**24**, the methylene carbon (CH_2_C=O) signal appears at δ 49.4–50.4 ppm. The CONHPh carbon resonates at δ 164.9–165.5 ppm, while the ketone carbonyl carbon gives a signal at δ 183.9–188.1 ppm. Also, the characteristic signals of Ph carbons are observed at δ 139.0–139.1 ppm (C-1), δ 119.0–119.1 ppm (C-2, C-6), δ 128.6–128.9 ppm (C-3, C-5) and δ 123.3–123.5 ppm (C-4). The thienopyridine core displays the following characteristic resonances: the C-2 signal appears at δ 100.3–104.0 ppm, C-3 at δ 146.6–152.3 ppm, C-3a at δ 108.8–122.5 ppm and C-4 at δ 143.6–148.2 ppm. The C-5 signal strongly depends on the substituents and is observed in the ranga of δ 119.0–132.3 ppm. Next, the C-6 signal appears at δ 159.7–162.9 ppm and C-7a at δ 153.5–162.9 ppm. The C(4)CH_3_ peak typically appears in the range δ 15.4–20.4 ppm, while the C(6)CH_3_ carbon gives a signal in the narrower range of δ 23.4–24.2 ppm.

**Figure 4:**
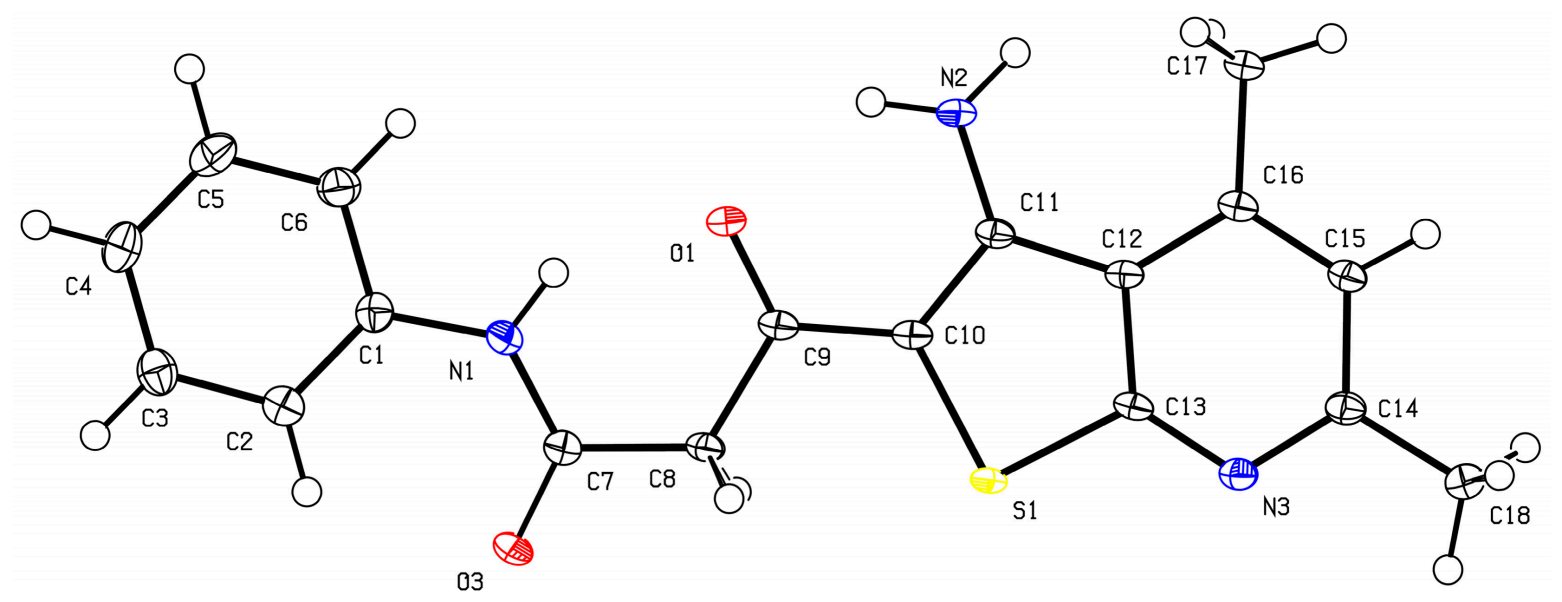
ORTEP drawing of X-ray structure for 4-[(3-cyano-4,6-dimethylpyridin-2-yl)thio]-3-oxo-*N*-phenylbutanamide **22a** with numbering system (not the IUPAC standard) and ellipsoids with 50% probability (CCDC deposition number 2576176).

The structure of 4-[(3-cyano-4,6-dimethylpyridin-2-yl)thio]-3-oxo-*N*-phenylbutanamide **22a** was studied by single-crystal X-ray diffraction analysis (Figure 4, Supplementary Materials File, Tables S1–S8).

**Scheme 5:**
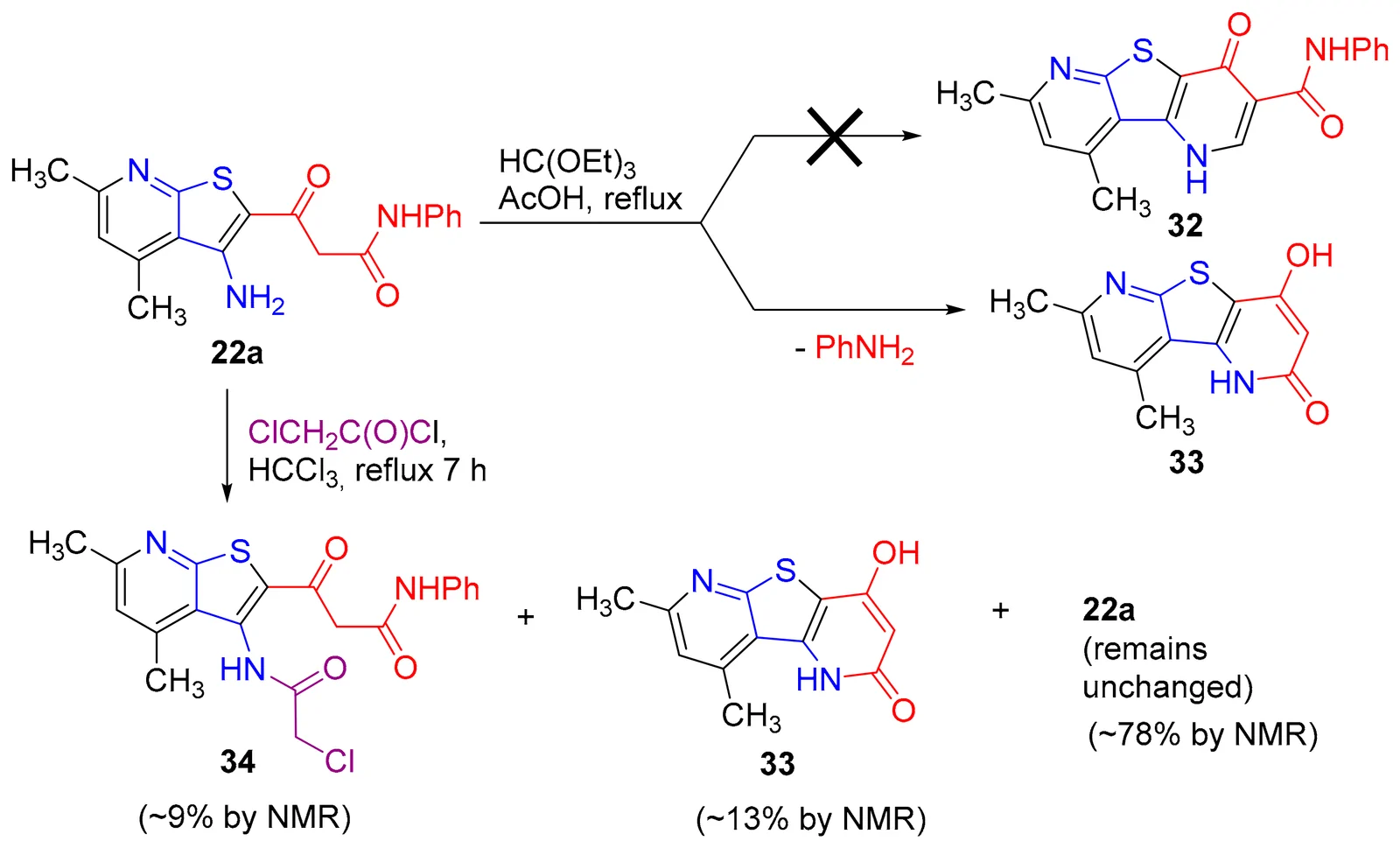
The reactions of β-ketoamide **22a**.

We also investigated some reactions of the model ketoamide **22a**. Thus, we found that treatment with triethyl orthoformate in glacial AcOH gave a new product, to which we initially assigned the dipyridothiophene structure **32**. However, according to NMR data, the new compound is actually thieno[2,3*-b*;4,5*-b*]dipyridine **33** (Scheme 5). The latter can also be obtained by prolonged heating of β-ketoamide **22a** in high-boiling solvents such as AcOH or DMF. Evidently, an intramolecular transamination occurs with elimination of an aniline molecule.

In our view, ketoamide **22a** does not react with triethyl orthoformate owing to insufficient CH acidity, which can be rationalized by the partially amidic character of the C=O group (a vinylogous amide). For the same reason, attempted acylation of **22a** with chloroacetyl chloride gave ambiguous results: along with the formation of a small amount of the desired chloroacetamide **34**, the intramolecular cyclization product **33** was also detected, and a portion of the starting material **22a** was recovered unchanged (Scheme 5). The ratio of **33**, **34**, and **22a** in the mixture was determined by integrating the signals in the ^1^H NMR spectrum of the reaction product.

Additionally, thienopyridine **22a** failed to react with semicarbazide hydrochloride (DMF, 80 °C, 2 h) and with 2,4-dinitrophenylhydrazine (HCl, DMF, 2 h); in both cases, the starting ketoamide **22a** was recovered unchanged.

**Scheme 6:**
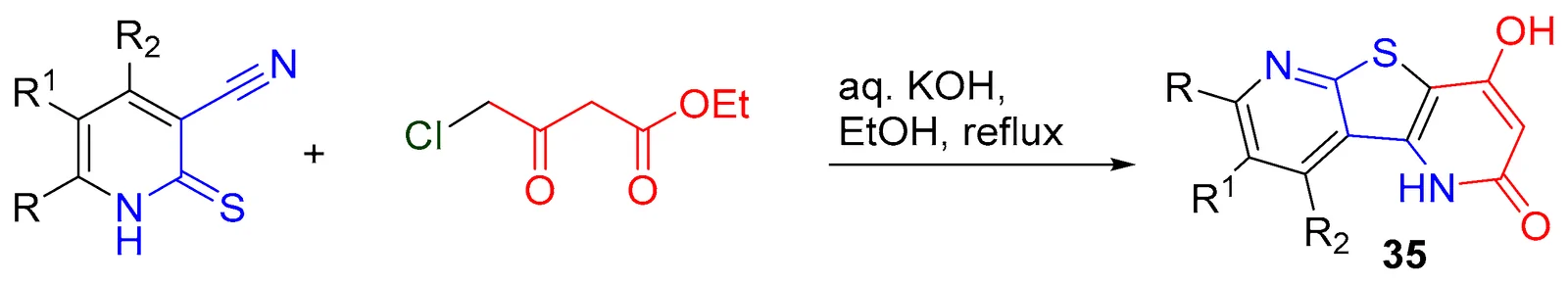
Preparation of thieno[2,3*-b*;4,5*-b*]dipyridines **35** starting from ethyl γ-chloroacetoacetate [92,93,94,95,96,97].

It should also be noted that compounds **35** similar to **33** are known in the literature [92,93,94,95,96,97]. Such thieno[2,3*-b*;4,5*-b*]dipyridines can be prepared in good yields by the reaction of 2-thioxopyridine-3-carbonitriles with ethyl γ-chloroacetoacetate (Scheme 6). The method we have discovered provides an alternative approach to such compounds, although it is obviously less efficient in terms of atom economy.

### 2.2. Quantum Chemical Studies

An interesting feature of the spectral data for compounds **22**–**24** is the absence of a pronounced keto carbonyl absorption band in their FTIR spectra. Moreover, the NMR spectra reveal no keto-enol tautomerism within the β-ketoamide fragment. For this reason, we performed quantum chemical calculations of the molecular geometry and vibrational frequencies of the IR spectrum for molecule **22a**.

The calculations for various conformers and tautomers of **22a** were performed using the ORCA 5.0.1 software package [98,99] with the hybrid DFT functional B3LYP [100,101] including the D3BJ dispersion correction [102] and the valence-triple-zeta basis set 6-311+G(2d,p). A comparative analysis of the energies of the obtained molecular structures in DMSO was carried out using the conductor-like polarizable continuum model (CPCM) [103]. The calculated vibrational frequencies were compared with the experimental data by applying scaling factors of 0.9679 for high-frequency modes (>1800 cm^−1^) and 1.0100 for low-frequency modes (<1800 cm^−1^) [104]. Frequency calculations were performed after a preliminary conformational search for the most stable species, followed by full geometry optimization. Molecular geometries and vibrational frequencies were visualized using the ChemCraft 1.8 program.

**Figure 5:**
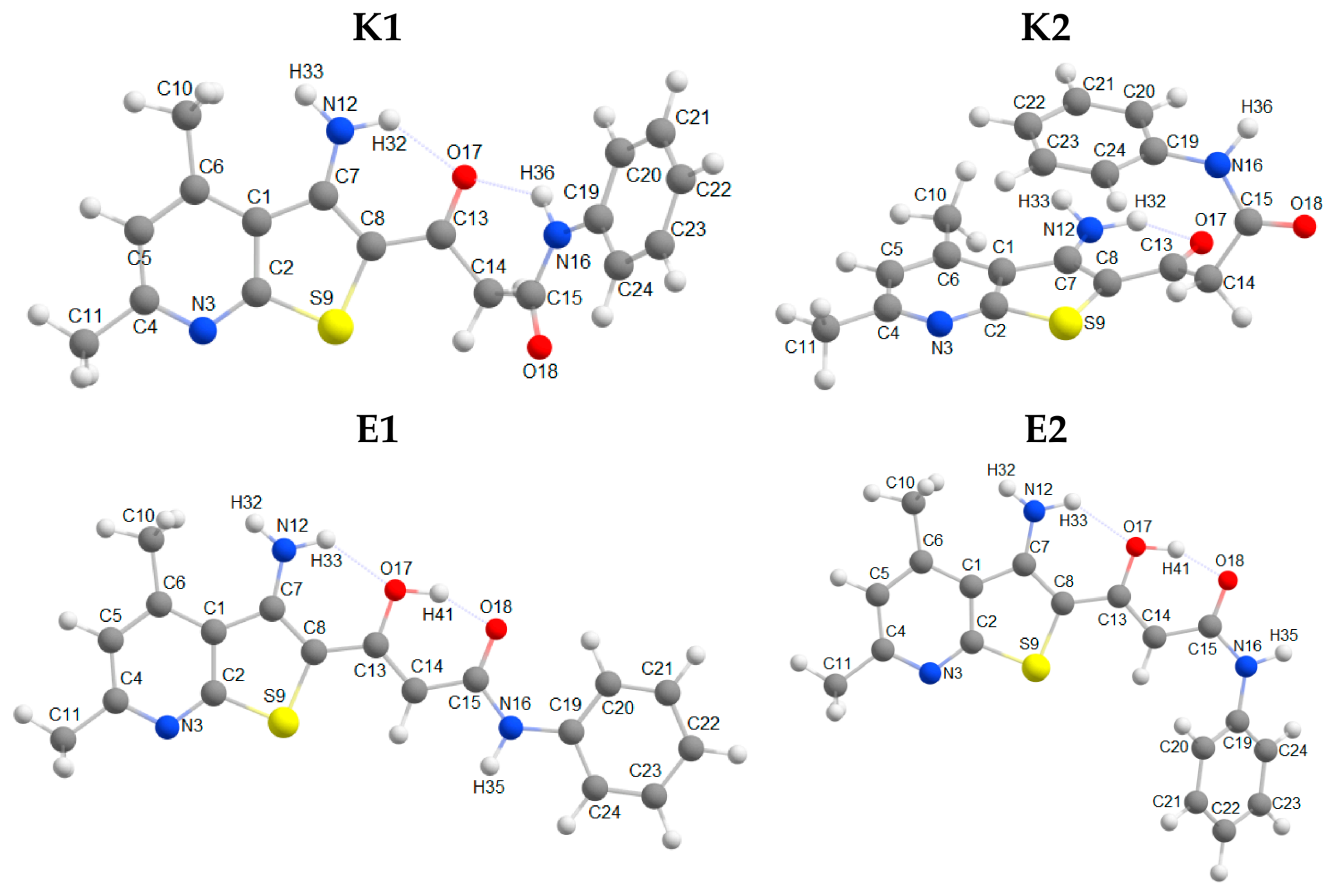
Optimized molecular structures of the most stable conformers of the keto (**K1**, **K2**) and enol (**E1**, **E2**) forms of thienopyridine **22a**, obtained at the B3LYP-D3BJ/6-311+G(2d,p) level of theory.

The molecule **22a** can exist as a considerable number of conformational isomers. According to the quantum-chemical calculations, the most stable conformers are **K1** and **K2**, whose molecular structures are shown in Figure 5. As we can see, both conformers have an intramolecular hydrogen bond between the oxygen atom and one of the amino group hydrogen atoms (N12–H32···O17), with bond lengths of 1.90 Å in **K1** and 1.89 Å in **K2**. The main stabilizing factor of conformer **K1** is an additional intramolecular hydrogen bond (1.97 Å) formed between the oxygen atom and the N–H bond of the carbamide group (N16–H36···O17). In conformer **K2**, the additional stabilization arises from a π–π stacking interaction between the phenyl and thieno[2,3-b]pyridine ring systems.

According to the computational results, conformer **K1** is the most stable. However, the energy difference between **K1** and **K2** is relatively small—13.5 kJ/mol in the gas phase and 4.1 kJ/mol in DMSO solution.

To evaluate the propensity of compound **22a** toward enolization, we performed quantum-chemical calculations for the enol form, including a preliminary search for its most stable conformers. The results showed that the most stable conformers of the enol form are **E1** and **E2** (Figure 5), with an energy difference of 4.3 kJ/mol in the gas phase and 1.8 kJ/mol in DMSO. In these conformers, the OH group forms two intramolecular hydrogen bonds (N12–H33···O17 and O17–H41···O18), with lengths of 1.98 and 1.56 Å for **E1**, and 1.97 and 1.53 Å for **E2**, respectively. According to the calculations, conformer **E1** has a planar structure, whereas in **E2** the phenyl ring is tilted at an angle of 47° relative to the molecular plane, which is attributed to repulsion between the hydrogen atoms attached to C14 and C20. Comparison of the energies of the most stable conformers of the ketone and enol forms (**K1** and **E1**, respectively) revealed that the ketone form is more stable. The energy difference between them is 16.4 kJ/mol in the gas phase and 27.5 kJ/mol in DMSO, which also indicates more efficient solvation of the ketone form. Calculation using the Gibbs equation gave an equilibrium constant for keto-enol tautomerism in DMSO under standard conditions of 1.52 × 10^−5^, indicating a very low concentration of the enol form, which is consistent with the ^1^H NMR spectroscopic data. A comparison of the calculated vibrational frequencies for the most stable ketone conformer, **K1**, with the experimental values is presented in Table 1.

As expected, the use of a scaling factor considerably improves the agreement between the calculated and experimental vibrational frequencies (the mean absolute percentage error (MAPE) for the most intense vibrational frequencies is 3.02% without the scaling factor and 1.40% with the scaling factor, respectively).

The absorption band of the ketone C=O undergoes a bathochromic shift due to conjugation with the amino group at the C–3 of the thienopyridine system. In the experimental IR spectrum, the C=O absorption band overlaps with the N–H bending vibration bands at 1589 cm^−1^. The calculated value for ν C=O, after scaling, is 1565 cm^−1^.

The experimental and calculated IR spectra for molecule **22a** are given in the Supplementary Materials File.

### 2.3. Agrochemical Studies

Some new compounds were evaluated for their herbicidal safener activity against 2,4-dichlorophenoxyacetic acid (2,4-D). 2,4-D is a widely used herbicide that has been reported to exhibit no significant toxicity to humans [105]. However, its application is associated with adverse side effects, including inhibitory effects on crop plants themselves, which can lead to yield losses of approximately 15–60%. To mitigate these negative effects and improve crop productivity, herbicide safeners (also referred to as herbicide antidotes) are successfully employed. The concept of herbicide safeners originated from Otto Hoffmann’s observation that tomato plants treated with 2,4,6-trichlorophenoxyacetic acid (2,4,6-T) did not exhibit the characteristic epinastic and swelling responses when subsequently exposed to vapors of 2,4-D [106].

Herbicide safeners (for recent reviews see [107,108,109,110,111,112,113]) are defined as agrochemicals capable of neutralizing phytotoxins in plants, thereby protecting crops from herbicide injury. They are harmless to crop plants and may even exert a growth-stimulating effect, while not diminishing the herbicidal activity against weeds.

It has been previously reported that 3-aminothieno[2,3-b]pyridines, which can be considered structural analogs of the synthesized compounds **22**–**24**, are effective herbicide safeners [17,50,51,52,53,54,55]. In the present study, we investigated the efficacy of the most accessible new compounds **22a**–**f** and **23** as 2,4-D antidotes using sunflower seedlings and following the procedure described in [114] (see also Materials and Methods). The antidote effect *A* was calculated as the ratio of the length of hypocotyls (or roots) in sunflower seedlings treated with the herbicide plus antidote to that in the reference group treated with 2,4-D alone, expressed as a percentage (Equation (1)):

*A* = (*L*_exp_/*L*_ref_) × 100% (1)

where *L*_exp_ is an organ length (mm) in the group treated with herbicide and antidote, and *L*_ref_ is an organ length (mm) in the reference group treated with 2,4-D alone.

Initial laboratory experiments revealed that all tested compounds exhibited an antidote effect against 2,4-D, albeit to varying degrees (Table 2).

As can be seen from the data in Table 2, the application of substituted 3-(3-aminothieno[2,3-b]pyridine-2-yl)-3-oxo-*N*-phenylpropanamides **22a**–**f** and **23**, proposed as herbicide safeners, substantially attenuated the toxic effects of the herbicide 2,4-D. Thus, thienopyridines **22a**–**f** and **23** exhibited an antidote effect at a level of 110–149% on the hypocotyls of sunflower seedlings, primarily at three or more tested concentrations. On the roots the antidote effect was even more pronounced (119–156%). Thienopyridines **22d**, **22f** and especially **22a** proved to be the most active among the tested series.

To further evaluate their protective efficacy, the safener activity of thienopyridines **22a**, **22d**, and **22f** was assessed under field conditions at the experimental station of the Federal Scientific Center for Biological Protection of Plants (Krasnodar, Russia). Sunflower plants (cv. Master) at the 10–16 leaf stage were sequentially treated with an aqueous solution of 2,4-D (18 g/ha), followed three days later by a foliar application of the test safener at a dose of 100 g/ha, with a working fluid volume of 300 L/ha. The field trial comprised three treatment variants:

- Control group—untreated plants;
- “Herbicide” (reference) group—plants were treated with herbicide 2,4-D only;
- “Herbicide + antidote” group—plants treated with herbicide 2,4-D and an antidote.

The experiment was conducted on 2.8 m^2^ plots in five replications, with harvesting performed at full seed maturity.

The field antidote effect *A_F_* was determined by the absolute value of the crop yield increase relative to the herbicide reference by Equation (2):

(2) $$A_{F}=\frac{Y_{1}-Y_{2}}{Y_{2}}\;\times \;100\%$$

where *A_F_*—field antidote effect, %; *Y*_1_—crop yield in “herbicide + antidote “ group; *Y*_2_—crop yield in “herbicide” (reference) group.

Statistical analysis was carried out using Student’s *t*-test. The results, summarized in Table 3, indicate that the application of compounds **22a**, **22d**, and **22f** as safeners produced a pronounced protective effect, with yield increases ranging from 46.4% to 57.2%.

## 3. Materials and Methods

^1^H, ^13^C and ^13^C DEPTQ NMR spectra were recorded in solutions of DMSO-d_6_ on a Bruker AVANCE-III HD instrument (Gottingen, Germany) (at 400.40 MHz for ^1^H or 100.61 MHz for ^13^C nuclei) and a JEOL JNM-ECA-400 instrument (JEOL, Tokyo, Japan) (at 399.78 MHz for ^1^H or 100.53 MHz for ^13^C nuclei). ^1^H-^13^C HSQC and HMBC 2D NMR experiments were performed on an Agilent 400/MR (Agilent Technologies, Santa Clara, CA, USA) in DMSO-d_6_. Residual solvent signals were used as internal standards in DMSO-d_6_—2.49 ppm for ^1^H, and 39.50 ppm for ^13^C nuclei. FT-IR spectra were recorded on a Bruker Vertex 70 instrument (Bruker Optik GmbH, Karlsruhe, Baden-Württemberg, Germany) equipped with an ATR sampling module or an Infraspec FSM2201 instrument (Infraspec, Saint-Petersburg, Russia) in Nujol mulls. Single-crystal X-ray diffraction analyses were performed on an Agilent SuperNova, Dual, Cu at home/near, Atlas diffractometer (Agilent Technologies, Santa Clara, CA, USA). See Electronic Supplementary Material File for NMR, FT-IR spectral charts and X-ray analysis data. Elemental analyses were carried out using a Carlo Erba 1106 Elemental Analyzer (Carlo Erba Instruments, Rodano, Milan, Italy). Reaction progress and purity of isolated compounds were controlled by TLC on Sorbfil-A plates (produced by Imid Ltd., Krasnodar, Russia), eluents—acetone:hexane 2:1, HCCl_3_-toluene 2:1, or ethyl acetate–light petroleum. Developed TLC plates were stained with UV-light and iodine vapors. The reagents and solvents were purchased from the commercial vendor (BioInLabs, Rostov-on-Don, Russia) and used as received.

**3-(3-Aminothieno[2,3-b]pyridine-2-yl)-3-oxo-*N*-phenylpropanamides** (**22a**–**h**) were prepared as follows: 2-thioxopyridine-3-carbonitriles **10a**–**h** (0.0025 mol) or thiolate **14** (0.63 g, 0.0025 mol) were suspended in EtOH (10–15 mL). To the resulting suspension, an aqueous 10% KOH (1.3 mL, 0.0025 mol) was added, followed by the addition of γ-bromoacetoacetanilide **8** [63] (0.64 g, 0.0025 mol). Upon addition, precipitation of the alkylation products **20** or **21** may be observed. The mixture was stirred for 30 min. Then, a second equivalent of 10% aqueous KOH (1.3 mL, 0.0025 mol) was added, which caused the intermediate precipitate of **20** to dissolve completely. The reaction mixture was heated to reflux and stirred for an additional 30 min. After cooling to room temperature, the reaction mixture was acidified with glacial AcOH (0.2 mL, 3.5 mmol) and allowed to stand for 12 h. The precipitated solid was collected by filtration, washed with water, and recrystallized from BuOH. The target products were isolated in 51–82% yields.

*3-(3-Amino-4,6-dimethylthieno[2,3-b]pyridin-2-yl)-3-oxo-N-phenylpropanamide* **22a** was obtained as yellow crystals in 433 mg (51%) yield from 2-thioxopyridine **10a** (410 mg, 2.5 mmol) and γ-bromoacetoacetanilide **8** (640 mg, 2.5 mmol). FTIR, ν_max_, cm^−1^: 3429, 3302, 3246, 3196 (N–H); 1676 (amide C=O); 1589 br (keto C=O). ^1^H NMR (400 MHz, DMSO-*d*_6_): 2.51 (s, 3H, C(6)–CH_3_), 2.71 (s, 3H, C(4)–CH_3_), 3.76 (s, 2H, CH_2_), 7.03–7.07 (m, 1H, C(4)H Ph), 7.08 (s, C(5)H), 7.28–7.32 (m, 2H, C(3)H, C(5)H Ph), 7.59 (d, ^3^*J* = 8.4 Hz, 2H, C(2)H, C(6)H Ph), 7.74 (br s, 2H, NH_2_), 10.21 (CONH). ^13^C DEPTQ NMR (101 MHz, DMSO-*d*_6_): 20.0* (C(4)–CH_3_), 24.0* (C(6)–CH_3_), 50.3 (CH_2_), 103.6 (C-2), 119.0* (2C, C-2, C-6 Ph), 121.8 (C-3a), 122.1* (C-5), 123.4* (C-4 Ph), 128.8* (2C, C-3, C-5 Ph), 139.0 (C-1 Ph), 146.1 (C-4), 151.1 (C-3), 160.6 (C-7a), 160.8 (C-6), 164.9 (s, 1H, CONH), 188.0 (C=O). *Negatively phased signals. Elemental Analysis: found, %: C, 63.74; H, 5.13; N, 12.35. C_18_H_17_N_3_O_2_S (M 339.41). Calculated, %: C, 63.70; H, 5.05; N, 12.38.

*X-ray studies for single crystals of* **22a**.

Single crystals of 3-(3-amino-4,6-dimethylthieno[2,3-b]pyridin-2-yl)-3-oxo-*N*-phenylpropanamide **22a** (C_18_H_17_N_3_O_2_S, *M* = 339.40 g/mol) were grown from BuOH. The crystal was kept at 99.9(2) K during data collection. Using Olex2 [115], the structure was solved with the SHELXT [116] structure solution program using Intrinsic Phasing and refined with the SHELXL [117] refinement package using least squares minimisation. The crystals are orthorhombic, space group Pca2_1_ (no. 29), *a* = 21.46758(18) Å, *b* = 5.33292(4) Å, *c* = 13.78236(14) Å, *V* = 1577.87(2) Å^3^, *Z* = 4, *T* = 99.9(2) K, *μ*(CuK_α_) = 1.959 mm^−1^, *D_calc_* = 1.429 g/cm^3^, with 12,902 reflections measured (8.238° ≤ 2*Θ* ≤ 152.524°), 2862 of them unique (*R*_int_ = 0.0258, R_sigma_ = 0.0203), which were used in all calculations. The final *R*_1_ was 0.0224 (*I* > 2σ(*I*)) and *wR*_2_ was 0.0600 (all data). A full set of crystallographic data has been deposited in the Cambridge Crystallographic Data Center (CCDC 2576176).

*3-(3-Amino-5-butyl-4,6-dimethylthieno[2,3-b]pyridin-2-yl)-3-oxo-N-phenylpropanamide* **22b** was obtained as yellow powder in 691 mg (70%) yield from thioxopyridine **10b** (550 mg, 2.5 mmol) and γ-bromoacetoacetanilide **8** (640 mg, 2.5 mmol). FTIR, ν_max_, cm^−1^: 3433, 3294, 3252, 3205 (N–H); 1679 (amide C=O); 1593 br (keto C=O). ^1^H NMR (400 MHz, DMSO-*d*_6_): 0.90–0.96 (m, 3H, CH_3_ Bu), 1.38–1.44 (m, 4H, CH_2_CH_2_), 2.57 (s, 3H, C(6)–CH_3_), 2.69 (s, 3H, C(4)–CH_3_), 2.70–2.72 (m, 2H, CH_2_ Bu), 3.75 (s, 2H, CH_2_), 7.03–7.06 (m, 1H, C(4)H Ph), 7.28–7.32 (m, 2H, C(3)H, C(5)H Ph), 7.59 (d, ^3^*J* = 7.7 Hz, 2H, C(2)H, C(6)H Ph), 7.85 (br s, 2H, NH_2_), 10.22 (s, 1H, CONH). ^13^C DEPTQ NMR (101 MHz, DMSO-*d*_6_): 13.8* (CH_3_ Bu), 15.6* (C(4)–CH_3_), 22.4 (CH_2_ Bu), 23.5* (C(6)–CH_3_), 27.6 (CH_2_ Bu), 31.2 (CH_2_ Bu), 50.3 (CH_2_), 103.7 (C-2), 119.0* (2C, C-2, C-6 Ph), 122.5 (C-3a), 123.3* (C-4 Ph), 128.8* (2C, C-3, C-5 Ph), 131.2 (C-5), 139.1 (C-1 Ph), 143.8 (C-4), 151.7 (C-3), 157.7 (C-7a), 159.8 (C-6), 165.0 (CONH), 188.0 (C=O). *Negatively phased signals. Elemental Analysis: found, %: C, 66.85; H, 6.47; N, 10.54. C_22_H_25_N_3_O_2_S (M 395.52). Calculated, %: C, 66.81; H, 6.37; N, 10.62.

*3-(5-Allyl-3-amino-4,6-dimethylthieno[2,3-b]pyridin-2-yl)-3-oxo-N-phenylpropanamide* **22c** was obtained as yellow powder in 750 mg (79%) yield from thioxopyridine **10c** (510 mg, 2.5 mmol) and γ-bromoacetoacetanilide **8** (640 mg, 2.5 mmol). FTIR, ν_max_, cm^−1^: 3487, 3313 br, 3196 (N–H); 1662 (amide C=O); 1594 br (keto C=O). ^1^H NMR (400 MHz, DMSO-*d*_6_): 2.52 (s, 3H, C(6)–CH_3_), 2.65 (s, 3H, C(4)–CH_3_), 3.49 (br d, ^3^*J* = 4.8 Hz, 2H, CH_2_CH=CH_2_), 3.78 (s, 2H, CH_2_C(O)), 4.78 (dd, ^3^*J* = 17.5 Hz, ^2^*J* = 1.4 Hz, *trans-*CH=CH_2_), 5.03 (dd, ^3^*J* = 9.9 Hz, ^2^*J* = 1.4 Hz, *cis-*CH=CH_2_), 5.90–5.99 (m, CH_2_CH=CH_2_), 7.02–7.06 (m, 1H, C(4)H Ph), 7.27–7.31 (m, 2H, C(3)H, C(5)H Ph), 7.60 (d, ^3^*J* = 7.9 Hz, 2H, C(2)H, C(6)H Ph), 7.85 (br s, 2H, NH_2_), 10.28 (s, 1H, CONH). ^13^C NMR (101 MHz, DMSO-*d*_6_): 15.7 (C(4)–CH_3_), 23.4 (C(6)–CH_3_), 31.8 (CH_2_CH=CH_2_), 50.3 (CH_2_), 103.9 (C-2), 115.5 (=CH_2_), 119.0 (2C, C-2, C-6 Ph), 122.4 (C-3a), 123.3 (C-4 Ph), 127.9 (C-5), 128.7 (2C, C-3, C-5 Ph), 135.0 (CH_2_CH=CH_2_), 139.0 (C-1 Ph), 144.6 (C-4), 151.6 (C-3), 158.2 (C-7a), 160.2 (C-6), 165.0 (CONH), 188.0 (C=O). Elemental Analysis: found, %: C, 66.40; H, 5.65; N, 11.03. C_21_H_21_N_3_O_2_S (M 379.48). Calculated, %: C, 66.47; H, 5.58; N, 11.07.

*3-(3-Amino-5-ethyl-4,6-dimethylthieno[2,3-b]pyridin-2-yl)-3-oxo-N-phenylpropanamide* **22d** was obtained as yellow powder in 753 mg (82%) yield from thioxopyridine **10d** (480 mg, 2.5 mmol) and γ-bromoacetoacetanilide **8** (640 mg, 2.5 mmol). FTIR, ν_max_, cm^−1^: 3491, 3321 br, 3298, 3196 (N–H); 1661 (amide C=O); 1595 br (keto C=O). ^1^H NMR (400 MHz, DMSO-*d*_6_): 1.08 (t, ^3^*J* = 7.3 Hz, CH_2_CH_3_), 2.57 (s, 3H, C(6)–CH_3_), 2.69 (s, 3H, C(4)–CH_3_), 2.73 (q, 2H, ^3^*J* = 7.3 Hz, CH_2_CH_3_, partially overlapped with the singlet at 2.69 ppm), 3.76 (s, 2H, CH_2_C(O)), 7.03–7.06 (m, 1H, C(4)H Ph), 7.28–7.32 (m, 2H, C(3)H, C(5)H Ph), 7.59 (d, ^3^*J* = 7.8 Hz, 2H, C(2)H, C(6)H Ph), 7.86 (br s, 2H, NH_2_), 10.21 (s, 1H, CONH). ^13^C DEPTQ NMR (101 MHz, DMSO-*d*_6_): 13.5* (CH_3_ Et), 15.4* (C(4)–CH_3_), 21.1 (CH_2_ Et), 23.4* (C(6)–CH_3_), 50.3 (CH_2_), 103.7 (C-2), 119.0* (2C, C-2, C-6 Ph), 122.5 (C-3a), 123.3* (C-4 Ph), 128.8* (2C, C-3, C-5 Ph), 132.3 (C-5), 139.0 (C-1 Ph), 143.6 (C-4), 151.7 (C-3), 157.7 (C-7a), 159.7 (C-6), 165.0 (CONH), 187.9 (C=O). *Negatively phased signals. Elemental Analysis: found, %: C, 65.32; H, 5.85; N, 11.40. C_20_H_21_N_3_O_2_S (M 367.47). Calculated, %: C, 65.37; H, 5.76; N, 11.44.

*3-(3-Amino-4,5,6-trimethylthieno[2,3-b]pyridin-2-yl)-3-oxo-N-phenylpropanamide* **22e** was obtained as yellow powder in 716 mg (81%) yield from thioxopyridine **10e** (446 mg, 2.5 mmol) and γ-bromoacetoacetanilide **8** (640 mg, 2.5 mmol). FTIR, ν_max_, cm^−1^: 3425, 3290 br, 3252, 3190 (N–H); 1674 (amide C=O); 1591 br (keto C=O). ^1^H NMR (400 MHz, DMSO-*d*_6_): 2.24 (s, 3H, C(5)–CH_3_), 2.53 (s, 3H, C(6)–CH_3_), 2.65 (s, 3H, C(4)–CH_3_), 3.76 (s, 2H, CH_2_C(O)), 7.02–7.06 (m, 1H, C(4)H Ph), 7.28–7.32 (m, 2H, C(3)H, C(5)H Ph), 7.59 (d, ^3^*J* = 8.3 Hz, 2H, C(2)H, C(6)H Ph), 7.85 (br s, 2H, NH_2_), 10.22 (s, 1H, CONH). ^13^C DEPTQ NMR (101 MHz, DMSO-*d*_6_): 14.4* (C(5)–CH_3_), 16.0* (C(4)–CH_3_), 24.2* (C(6)–CH_3_), 50.3 (CH_2_), 103.8 (C-2), 119.0* (2C, C-2, C-6 Ph), 122.2 (C-3a), 123.3* (C-4 Ph), 126.8 (C-5), 128.8* (2C, C-3, C-5 Ph), 139.1 (C-1 Ph), 143.8 (C-4), 151.6 (C-3), 157.5 (C-7a), 160.0 (C-6), 165.0 (CONH), 187.9 (C=O). *Negatively phased signals. Elemental Analysis: found, %: C, 64.54; H, 5.48; N, 11.83. C_19_H_19_N_3_O_2_S (M 353.44). Calculated, %: C, 64.57; H, 5.42; N, 11.89.

*3-[3-Amino-4,6-dimethyl-5-(2-oxo-2-phenylethyl)thieno[2,3-b]pyridin-2-yl]-3-oxo-N-phenylpropanamide* **22f** was obtained as yellow powder in 938 mg (82%) yield from thioxopyridine **10f** (706 mg, 2.5 mmol) and γ-bromoacetoacetanilide **8** (640 mg, 2.5 mmol). FTIR, ν_max_, cm^−1^: 3487, 3281 br, 3184 (N–H); 1678 (benzoyl C=O); 1651 (amide C=O); 1585 br (keto C=O). ^1^H NMR (400 MHz, DMSO-*d*_6_): 2.44 (s, 3H, C(6)–CH_3_), 2.57 (s, 3H, C(4)–CH_3_), 3.79 (s, 2H, CH_2_C(O)), 4.73 (s, 2H, CH_2_Bz), 7.03–7.07 (m, 1H, C(4)H Ph), 7.29–7.33 (m, 2H, C(3)H, C(5)H Ph), 7.59–7.62 (m, 4H, Ph, Bz), 7.70–7.73 (m, 1H, C(4)H Bz), 7.87 (br s, 2H, NH_2_), 8.16 (d, ^3^*J* = 7.3 Hz, 2H, C(2)H, C(6)H Bz), 10.22 (s, 1H, CONH). ^13^C DEPTQ NMR (101 MHz, DMSO-*d*_6_): 16.3* (C(4)–CH_3_), 23.9* (C(6)–CH_3_), 38.6 (CH_2_Bz), 50.4 (CH_2_), 103.9 (C-2), 119.0* (2C, C-2, C-6 Ph), 122.3 (C-3a), 123.4* (C-4 Ph), 125.7 (C-5), 128.3* (2C, C-3, C-5 Bz), 128.8* (2C, C-3, C-5 Ph), 128.9* (2C, C-2, C-6 Bz), 133.7* (C-4 Bz), 136.3 (C-1 Bz), 139.1 (C-1 Ph), 145.5 (C-4), 151.6 (C-3), 158.6 (C-7a), 160.6 (C-6), 165.0 (CONH), 188.1 (C=O), 196.9 (C=O Bz). *Negatively phased signals. Elemental Analysis: found, %: C, 68.19; H, 5.14; N, 9.14. C_26_H_23_N_3_O_3_S (M 457.55). Calculated, %: C, 68.25; H, 5.07; N, 9.18.

*3-(3-Amino-4,6-dimethyl-5-pentylthieno[2,3-b]pyridin-2-yl)-3-oxo-N-phenylpropanamide* **22g** was obtained as yellow powder in 778 mg (76%) yield from thioxopyridine **10g** (586 mg, 2.5 mmol) and γ-bromoacetoacetanilide **8** (640 mg, 2.5 mmol). FTIR, ν_max_, cm^−1^: 3481, 3319, 3275 (N–H); 1668 (amide C=O); 1601 br (keto C=O). ^1^H NMR (400 MHz, DMSO-*d*_6_): 0.87–0.90 (m, 3H, CH_3_ pentyl), 1.31–1.43 (m, 6H, (CH_2_)_3_), 2.56 (s, 3H, C(6)–CH_3_), 2.65–2.71 (s, 3H, C(4)–CH_3_ and m, 2H, CH_2_ pentyl overlapped), 3.75 (s, 2H, CH_2_), 7.03–7.08 (m, 1H, C(4)H Ph), 7.28–7.32 (m, 2H, C(3)H, C(5)H Ph), 7.59 (d, ^3^*J* = 8.0 Hz, 2H, C(2)H, C(6)H Ph), 7.85 (br s, 2H, NH_2_), 10.21 (s, 1H, CONH). ^13^C DEPTQ NMR (101 MHz, DMSO-*d*_6_): 14.0* (CH_3_ pentyl), 15.6* (C(4)–CH_3_), 21.9 (CH_2_ pentyl), 23.5* (C(6)–CH_3_), 27.8 (CH_2_ pentyl), 28.7 (CH_2_ pentyl), 31.5 (CH_2_ pentyl), 50.3 (CH_2_), 103.7 (C-2), 119.0* (2C, C-2, C-6 Ph), 122.5 (C-3a), 123.3* (C-4 Ph), 128.8* (2C, C-3, C-5 Ph), 131.2 (C-5), 139.1 (C-1 Ph), 143.8 (C-4), 151.7 (C-3), 157.7 (C-7a), 159.8 (C-6), 165.0 (CONH), 187.9 (C=O). *Negatively phased signals. Elemental Analysis: found, %: C, 67.32; H, 6.77; N, 10.17. C_23_H_27_N_3_O_2_S (M 409.55). Calculated, %: C, 67.45; H, 6.65; N, 10.26.

*3-[3-Amino-4-(methoxymethyl)-6-methylthieno[2,3-b]pyridin-2-yl]-3-oxo-N-phenylpropanamide* **22h** was obtained as yellow powder in 711 mg (77%) yield from thioxopyridine **10h** (486 mg, 2.5 mmol) and γ-bromoacetoacetanilide **8** (640 mg, 2.5 mmol). FTIR, ν_max_, cm^−1^: 3389, 3284 br (N–H); 1657 (amide C=O); 1593 br (keto C=O). ^1^H NMR (400 MHz, DMSO-*d*_6_): 2.57 (s, 3H, C(6)–CH_3_), 3.36 (s, 3H, OCH_3_), 3.78 (s, 2H, CH_2_C(O)), 4.83 (s, 2H, CH_2_OMe), 7.03–7.07 (m, 1H, C(4)H Ph), 7.28–7.32 (m, 3H, C(3)H, C(5)H Ph and C(5)H overlapped), 7.59 (d, ^3^*J* = 7.5 Hz, 2H, C(2)H, C(6)H Ph), 7.81 (br s, 2H, NH_2_), 10.22 (CONH). ^13^C DEPTQ NMR (101 MHz, DMSO-*d*_6_): 24.2* (C(6)–CH_3_), 50.3 (CH_2_), 57.7* (OMe), 71.4 (CH_2_OMe), 104.0 (C-2), 119.0* (2C, C-2, C-6 Ph), 120.4* (C-5), 121.4 (C-3a), 123.4* (C-4 Ph), 128.8* (2C, C-3, C-5 Ph), 139.0 (C-1 Ph), 144.5 (C-4), 150.1 (C-3), 160.8 (C-7a), 161.3 (C-6), 164.9 (CONH), 188.0 (C=O). *Negatively phased signals. Elemental Analysis: found, %: C, 61.74; H, 5.23; N, 11.33. C_19_H_19_N_3_O_3_S (M 369.44). Calculated, %: C, 61.77; H, 5.18; N, 11.37.

*3-(3-Amino-4-methyl-6-oxo-6,7-dihydrothieno[2,3-b]pyridin-2-yl)-3-oxo-N-phenylpropanamide* **23** was obtained as yellow powder in 460 mg (54%) yield from thiolate **14** (633 mg, 2.5 mmol) and γ-bromoacetoacetanilide **8** (640 mg, 2.5 mmol). FTIR, ν_max_, cm^−1^: 3416, 3282, 3225 (N–H); 1664 (amide C=O); 1593 br (keto C=O). ^1^H NMR (400 MHz, DMSO-*d*_6_): 2.53 (s, 3H, C(4)–CH_3_), 3.63 (s, 2H, CH_2_C(O)), 6.18 (s, 1H, C(5)H), 7.02–7.06 (m, 1H, C(4)H Ph), 7.28–7.32 (m, 3H, C(3)H, C(5)H Ph), 7.58 (d, ^3^*J* = 8.1 Hz, 2H, C(2)H, C(6)H Ph), 7.67 (br s, 2H, NH_2_), 10.18 (s, 1H, CONHPh), 12.39 (very br s, ~1H, pyridine CONH). ^13^C DEPTQ NMR (101 MHz, DMSO-*d*_6_): 20.4* (C(4)–CH_3_), 50.0 (CH_2_), 100.7 (C-2), 119.0* (3C, C-2, C-6 Ph and C(5)H overlapped), 123.4* (C-4 Ph), 128.8* (2C, C-3, C-5 Ph), 139.1 (C-1 Ph), 148.2 (C-4), 152.3 (C-3), 162.9 (C-7a), 165.1 (CONH), 185.9 (C=O). *Negatively phased signals. Elemental Analysis: found, %: C, 59.75; H, 4.57; N, 12.20. C_17_H_15_N_3_O_3_S (M 341.39). Calculated, %: C, 59.81; H, 4.43; N, 12.31.

*4-[(3-Cyano-5-ethyl-4,6-dimethylpyridin-2-yl)thio]-3-oxo-N-phenylbutanamide* **20d** was prepared as follows: 5-ethyl-4,6-dimethyl-2-thioxo-1,2-dihydropyridine-3-carbonitrile **10d** (480 mg, 0.0025 mol) was suspended in EtOH (5 mL). To the resulting suspension, an aqueous 10% KOH (1.3 mL, 0.0025 mol) was added, followed by the addition of γ-bromoacetoacetanilide **8** (0.64 g, 0.0025 mol). The mixture was stirred for 30 min and diluted with water. The precipitated solid was collected by filtration, washed with water and light petroleum. The intermediate crude S-alkylation product **20d** was isolated as a beige solid in 570 mg (62%) yield. FTIR, ν_max_, cm^−1^: 3290, 3256, 3194 (N–H, C–H); 2222 (C≡N); 1713 (ketone C=O); 1674 (amide C=O). ^1^H NMR (400 MHz, DMSO-*d*_6_): 1.02 (t, ^3^*J* = 7.3 Hz, CH_2_CH_3_), 2.41 (s, 3H, C(6)–CH_3_), 2.46 (s, 3H, C(4)–CH_3_), 2.58 (q, 2H, ^3^*J* = 7.3 Hz, CH_2_CH_3_), 3.77 (s, 2H, CH_2_C(O)), 4.27 (s, 2H, SCH_2_), 7.03–7.08 (m, 1H, C(4)H Ph), 7.27–7.31 (m, 2H, C(3)H, C(5)H Ph), 7.55 (d, ^3^*J* = 7.6 Hz, 2H, C(2)H, C(6)H Ph), 10.11 (s, 1H, CONH). ^13^C DEPTQ NMR (101 MHz, DMSO-*d*_6_): 12.6* (CH_3_ Et), 17.3* (C(4)–CH_3_), 21.1 (CH_2_ Et), 22.5* (C(6)–CH_3_), 39.7 (SCH_2_), 50.8 (CH_2_), 104.7 (C-C≡N), 115.5 (C≡N), 119.0* (2C, C-2, C-6 Ph), 123.4* (C-4 Ph), 128.8* (2C, C-3, C-5 Ph), 132.3 (C-5), 138.8 (C-1 Ph), 150.0 (C-4), 156.0 (C-6), 159.8 (C-2), 164.5 (CONH), 198.5 (C=O). *Negatively phased signals. Elemental Analysis: found, %: C, 65.24; H, 5.93; N, 11.30. C_20_H_21_N_3_O_2_S (M 367.47). Calculated, %: C, 65.37; H, 5.76; N, 11.44.

*4,5,6,7-Tetrahyrdothieno[2,3-b]pyridines* **24a**,**b**. General Procedure. To a suspension of thiolate **17a**,**b** [71,72] (500 mg, 1.37 mmol of **17a** or 1.49 mmol of **17b**) in EtOH (12 mL), an equimolar amount of γ-bromoacetoacetanilide **8** (351 mg, 1.37 mmol for thiolate **17a**; 382 mg, 1.49 mmol for thiolate **17b**) was added. The mixture was stirred at reflux for 30 min, then cooled, and Et_3_N (0.28 mL, 2 mmol) was added. The solution, which acquired a yellow-orange color, was refluxed for an additional 30 min (conversion was monitored by TLC). After cooling, the mixture was allowed to stand at ambient temperature until crystallization occurred. The precipitated product was collected by filtration, washed with aqueous ethanol and petroleum ether. The obtained product was sufficiently pure for analytical purposes.

*3-(3-Amino-4-(2-chlorophenyl)-6-oxo-4,5,6,7-tetrahydrothieno[2,3-b]pyridin-2-yl)-3-oxo-N-phenylpropanamide* **24a** was isolated as an off-white solid in 319 mg (53%) yield. FTIR, ν_max_, cm^−1^: 3460, 3410, 3306 br, 3167 br (N–H); 1686, 1659 (amide C=O); 1597 (ketone C=O). ^1^H NMR (400 MHz, DMSO-*d*_6_): 2.63 (br d—unresolved dd, ^2^*J* = 16.0 Hz, 1H, *cis-*CH_2_CHAr), 3.19 (dd, ^2^*J* = 16.0 Hz, ^3^*J* = 7.9 Hz, 1H, *trans-*CH_2_CHAr), 3.58 (s, 2H, CH_2_C(O)), 4.57–4.59 (m—unresolved dd, 1H, CH_2_CHAr), 6.66 (dd, 1H, ^3^*J* = 7.5 Hz, ^4^*J* = 1.6 Hz, CH Ar), 7.02–7.06 (m, 1H, C(4)H Ph), 7.22–7.32 (m, 4H, C(3)H, C(5)H Ph, 2H Ar), 7.36 (very br s, 2H, NH_2_), 7.50 (dd, ^3^*J* = 7.8 Hz, ^4^*J* = 1.4 Hz, 1H, CH Ar), 7.58 (dd, ^3^*J* = 7.8 Hz, ^4^*J* = 0.9 Hz, 2H, C(2)H, C(6)H Ph), 10.17 (s, 1H, CONHPh), 11.10 (s, 1H, endocyclic CONH). ^13^C NMR (101 MHz, DMSO-*d*_6_): 32.3 (C(5)H_2_), 36.8 (C(4)H), 49.5 (CH_2_C(O)), 100.6 (C-2), 109.0 (C-3a), 119.1 (2C, C-2, C-6 Ph), 123.5 (C-4 Ph), 127.4 (CH Ar), 127.7 (CH Ar), 128.9 (2C, C-3, C-5 Ph), 129.1 (CH Ar), 130.5 (CH Ar), 133.1 (C–Cl), 137.4 (C-1 Ar), 139.1 (C-1 Ph), 147.8 (C-3), 153.5 (C-7a), 165.5 (CONHPh), 168.6 (CONH endocyclic), 184.1 (C=O). Elemental Analysis: found, %: C, 60.11; H, 4.20; N, 9.52. C_22_H_18_ClN_3_O_3_S (M 439.91). Calculated, %: C, 60.07; H, 4.12; N, 9.55.

*3-(3-Amino-4-(5-methylfuran-2-yl)-6-oxo-4,5,6,7-tetrahydrothieno[2,3-b]pyridin-2-yl)-3-oxo-N-phenylpropanamide* **24b** was isolated as a tan/beige-colored powder in 128 mg (21%) yield. FTIR, ν_max_, cm^−1^: 3456, 3333, 3267 br, 3186 (N–H); 1697, 1670 (amide C=O); 1585 br (ketone C=O). ^1^H NMR (400 MHz, DMSO-*d*_6_): 2.17 (br s, 3H, Me), 2.63 (br d—unresolved dd, ^2^*J* = 16.0 Hz, 1H, *cis-*CH_2_CHAr), 3.02 (dd, ^2^*J* = 16.0 Hz, ^3^*J* = 7.5 Hz, 1H, *trans-*CH_2_CHAr), 3.53 (s, 2H, CH_2_C(O)), 4.34–4.36 (m—unresolved dd, 1H, CH_2_CHAr), 5.83 (br d, ^3^*J* = 3.2 Hz, CH furyl), 5.91 (dd, 1H, ^3^*J* = 3.2 Hz, ^4^*J* = 1.1 Hz, CH furyl), 7.02–7.05 (m, 1H, C(4)H Ph), 7.27–7.31 (m, 2H, C(3)H, C(5)H Ph), 7.48 (very br s, 2H, NH_2_), 7.57 (d, ^3^*J* = 7.8 Hz, 2H, C(2)H, C(6)H Ph), 10.13 (s, 1H, CONHPh), 10.94 (s, 1H, endocyclic CONH). ^13^C NMR (101 MHz, DMSO-*d*_6_): 13.5 (Me), 28.8 (C(5)H_2_), 35.3 (C(4)H), 49.4 (CH_2_C(O)), 100.3 (C-2), 106.2 (CH furyl), 106.4 (CH furyl), 108.8 (C-3a), 119.1 (2C, C-2, C-6 Ph), 123.4 (C-4 Ph), 128.6 (2C, C-3, C-5 Ph), 139.1 (C-1 Ph), 146.6 (C-3), 151.2 (C furyl), 152.2 (C furyl), 153.6 (C-7a), 165.5 (CONHPh), 168.9 (CONH endocyclic), 183.9 (C=O). Elemental Analysis: found, %: C, 61.56; H, 4.80; N, 10.27. C_21_H_19_N_3_O_4_S (M 409.46). Calculated, %: C, 61.60; H, 4.68; N, 10.26.

*(E)-2-{2-[2-(2-chlorophenyl)-1-cyanovinyl]thiazol-4-yl}-N-phenylacetamide* **25**. To a suspension of Michael adduct **19a** (1.4 g, 3.0 mmol), prepared in situ starting from cyanothioacetamide **12**, 2-chlorobenzaldehyde, and Meldrum’s acid (2,2-dimethyl-1,3-dioxane-4,6-dione) **18** in the presence of *N*-methylmorpholine [71,72], in EtOH (15 mL) was added γ-bromoacetoacetanilide **8** (770 mg, 3.0 mmol). The mixture was heated at 50 °C with stirring until complete homogenization, after which the resulting red-orange solution was allowed to stand at ambient temperature until crystallization occurred. The precipitated crystals were collected by filtration, washed with chilled ethanol and petroleum ether. The yield was 240 mg (21%), off-white crystals. FTIR, ν_max_, cm^−1^: 3306 (N–H); 2222 (conjugated C≡N); 1655 br (amide C=O). ^1^H NMR (400 MHz, DMSO-*d*_6_): 3.92 (s, 2H, CH_2_CONH), 7.02–7.06 (m, 1H, C(4)H Ph), 7.28–7.32 (m, 2H, C(3)H, C(5)H Ph), 7.53–7.57 (m, 2H, CH Ar), 7.60–7.65 (m, 3H, C(2)H, C(6)H Ph, CH Ar), 7.74 (s, 1H, C(5)H thiazole), 8.05–8.07 (m, 1H, CH Ar), 8.33 (s, 1H, ArCH=), 10.27 (s, 1H, CONHPh). ^13^C DEPTQ NMR (101 MHz, DMSO-*d*_6_): 39.1 (CH_2_), 108.7 (=C(CN)), 115.6 (C≡N), 119.1* (2C, C-2, C-6 Ph), 119.7* (C-5 thiazolyl), 123.4* (C-4 Ph), 127.8* (CH Ar), 128.8* (2C, C-3, C-5 Ph), 129.6* (CH Ar), 130.0* (CH Ar), 130.9 (C Ar), 132.8* (CH Ar), 134.0 (C Ar), 139.1 (C-1 Ph), 140.6* (ArCH=), 152.2 (C-4 thiazolyl), 160.6 (C-2 thiazolyl), 167.4 (CONH). *Negatively phased signals. Elemental Analysis: found, %: C, 63.18; H, 3.83; N, 11.02. C_20_H_14_ClN_3_OS (M 379.86). Calculated, %: C, 63.24; H, 3.72; N, 11.06.

*The reaction of ketoamide* **22a** *with triethyl orthoformate*. A mixture of 3-(3-amino-4,6-dimethylthieno[2,3-b]pyridin-2-yl)-3-oxo-*N*-phenylpropanamide **22a** (250 mg, 0.74 mmol) and a three-fold excess of triethyl orthoformate (0.37 mL, 2.2 mmol) was refluxed for 6 h in glacial acetic acid (6 mL). The crystals (110 mg) that precipitated on cooling were collected by filtration and washed with ethanol and petroleum ether. According to NMR data, the obtained product was a mixture of the starting material **22a** and the intramolecular cyclization product, 4-hydroxy-7,9-dimethylthieno[2,3*-b*:4,5*-b*′]dipyridine-2(1*H*)-one **27**.

*Synthesis of 4-hydroxy-7,9-dimethylthieno[2,3-b:4,5-b′]dipyridine-2(1H)-one* **33**. 3-(3-Amino-4,6-dimethylthieno[2,3-b]pyridin-2-yl)-3-oxo-*N*-phenylpropanamide **22a** (250 mg, 0.74 mmol) was refluxed for 18 h in glacial acetic acid (4 mL). The crystals that precipitated on cooling were collected by filtration and washed with ethanol and petroleum ether. Thienodipyridine **33** was isolated as an off-white powder in 80 mg (44%) yield. A similar reaction but carried out in DMF (reflux for 10 h), followed by precipitation with water and recrystallization from EtOH-acetone, afforded the same product **33** in 100 mg (55%) yield. FTIR, ν_max_, cm^−1^: 3483, 3433, 3261 (O–H, N–H); 1633 br (C=O). ^1^H NMR (400 MHz, DMSO-*d*_6_): 2.54 (s, 3H, C(7)CH_3_), 2.90 (s, 3H, C(9)CH_3_), 6.20 (s, 1H, C(3)H), 7.16 (s, 1H, C(8)H), 10.56 (br s, 1H, OH), 11.55 (br s, 1H, CONH). The observed signals in ^13^C DEPTQ NMR (101 MHz, DMSO-*d*_6_) of **33**: 19.0* (C(9)CH_3_), 23.9* (C(7)CH_3_), 93.2* (C-3), 121.9* (C-8), 123.8 (C-9a), 145.2, 157.6, 160.6, 160.9, 163.8. *Negatively phased signals. Elemental Analysis: found, %: C, 58.44; H, 4.17; N, 11.33. C_12_H_10_N_2_O_2_S (M 246.28). Calculated, %: C, 58.52; H, 4.09; N, 11.37.

*The reaction of β-ketoamide* **22a** *with chloroacetyl chloride.* 3-Aminothieno[2,3-b]pyridine **22a** (250 mg, 0.74 mmol) was suspended in 10 mL of water-free chloroform (dried over P_2_O_5_) and treated with a slight excess (1.1 equiv.) of chloroacetyl chloride (0.65 mL, 0.81 mmol). The resulting heterogeneous mixture was refluxed for 7 h under moisture-free conditions. After cooling, the product (170 mg) was filtered off and washed with petroleum ether. According to NMR spectroscopy data, the product consisted predominantly of starting **22a** (~78 mol%), thienodipyridine **33** (~13 mol%), and approximately 9% of chloroacetamide **34**.

## 4. Conclusions

Thus, we have developed a convenient synthetic route to novel 3-(3-aminothieno[2,3-b]pyridin-2-yl)-3-oxo-*N*-phenylpropanamides starting from readily available 2-thioxopyridine-3-carbonitriles or 3-cyanopyridine-2-thiolates and γ-bromoacetoacetanilide. The key step involves selective S-alkylation followed by the Thorpe-Ziegler cyclization, which proceeds efficiently under basic conditions to afford the target products in moderate to good yields (up to 82%). Using readily available *N*-methylmorpholinium 3-cyano-1,4,5,6-tetrahydropyridine-2-thiolates under analogous conditions but with triethylamine instead of KOH as a weaker base, two new 4,5,6,7-tetrahydrothieno[2,3-b]pyridines were prepared. We have also observed that the Michael adduct derived from cyanothioacetamide, 2-ClC_6_H_4_CHO and Meldrum’s acid, when subjected to the same reaction conditions, does not yield a tetrahydrothienopyridine but instead undergoes a *retro*-Michael fragmentation and subsequent cyclization to give a Hantzsch-type product, (*E*)*-*2-{2-[2-(2-chlorophenyl)-1-cyanovinyl]thiazol-4-yl}-*N*-phenylacetamide. This competing pathway highlights the sensitivity of the reaction to the nature of the starting S-nucleophile. The structure of the representative product, 4-[(3-cyano-4,6-dimethylpyridin-2-yl)thio]-3-oxo-*N*-phenylbutanamide **22a**, was confirmed by single-crystal X-ray diffraction, and detailed spectroscopic characterization including IR, ^1^H and ^13^C DEPTQ NMR, ^1^H–^13^C HSQC and ^1^H–^13^C HMBC 2D NMR experiments were provided for new compounds.

Quantum chemical calculations revealed that the ketone form of β-ketoamide **22a** is thermodynamically preferred over the enol tautomer, with a negligibly small enol fraction in solution, in full agreement with the experimental NMR observations. The use of scaling factors for the calculated vibrational frequencies allowed for an excellent match between theoretical and experimental IR spectra.

Furthermore, we have identified an unexpected intramolecular transamination of β-ketoamide **22a** under thermal conditions in AcOH or DMF with elimination of aniline, leading to 4-hydroxy-7,9-dimethylthieno[2,3*-b*:4,5*-b*′]dipyridin-2(1*H*)-one **33**. This reaction represents a novel and potentially useful method for accessing thieno[2,3*-b*;4,5*-b*]dipyridines.

Agrochemical evaluation demonstrated that thienopyridines **22a-f** and **23** possess significant herbicidal safener activity against 2,4-D, as shown by both laboratory germination tests and field trials on sunflower. Compounds **22a**, **22d** and **22f** were particularly effective, producing yield increases of 46–57% under field conditions. These findings suggest that the newly synthesized thienopyridine β-ketoamides hold promise for the development of effective herbicide antidotes to mitigate the phytotoxic effects of 2,4-D on cultivated crops. Further optimization of the structure and exploration of the mechanism of action are currently underway.

## Figures and Tables

**Table 1:** Comparison of experimental vibrational frequencies with the results of quantum-chemical calculation for the thienopyridine ketoamide **22a’s** most stable conformer **K1**.

Vibrations	Experimental Bands, cm^−1^	Calculated Vibrational Frequencies, cm^−1^	
		No Correction Factors	With Correction Factors *
ν_as_ NH_2_	3429	3732	3612
ν_s_ NH_2_	3302	3481	3369
ν NH	3250	3475	3363
ν C(Ar)-H	3076 3051	3186 3173	3084 3071
ν CH_3_	2972	3110	3010
ν C=O (amide I)	1674	1743	1687
δ_plane_ NH_2_ + δ _plane_ NH (amide II) + keto C=O	1589 (br)	1642 1625 1617	1589 1573 1565
ν C-C(Ar) + ν C-X(Ar) (skeletal)	1541 (br)	1587 1578	1536 1527
δ_as_ CH_3_	1467	1502	1454
ν C-NH_2_ (group vibrations)	1429 (br)	1467	1420
skeletal	1367	1376	1332
ω CH_2_	1313	1309	1267
skeletal	1230	1284	1243
skeletal	1190	1229	1190
ρ NH_2_	1088	1141	1104
skeletal	993	1033	1000
δ_out-of-plane_ C(Ar)-H	756	764	772
δ_out-of-plane_ NH (amide V)	739 (shoulder)	742	749
skeletal	690	684	691
ρ CH_2_	525	545	550
MAPE **, %	–	3.02	1.40

* The correction factors were 0.9679 for high frequency vibrations (>1800 cm^−1^) and 1.0100 for low frequency vibrations (<1800 cm^−1^) according to [104]. ** MAPE stands for mean absolute percentage error.

**Table 2:** Laboratory assay results for the antidote effect of thienopyridines **22a**–**f** and **23** against 2,4-D on sunflower seedlings.

N	Compound	Organ	Antidote Effect *A* at Different Concentrations, % ^1^			
			10^−2^	10^−3^	10^−4^	10^−5^
1	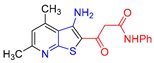 **22a**	roots	149	153	153	156
		hypocotyls	149	140	146	149
2	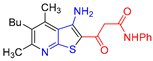 **22b**	roots	127	133	135	137
		hypocotyls	118	129	126	129
3	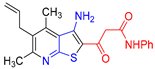 **22c**	roots	119	130	126	127
		hypocotyls	110	138	128	127
4	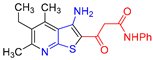 **22d**	roots	124	140	138	140
		hypocotyls	117	126	140	143
5	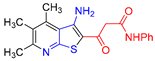 **22e**	roots	139	137	142	136
		hypocotyls	139	139	140	129
6	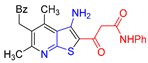 **22f**	roots	133	142	144	140
		hypocotyls	131	140	134	137
7	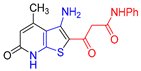 **23**	roots	130	139	144	142
		hypocotyls	133	137	137	137

^1^ The differences are statistically significant at *p* = 0.95.

**Table 3:** Results of field tests on the antidote activity of thienopyridines **22a**,**d**,**f** against the herbicide 2,4-D on sunflower cv. Master.

N	Compound	Field Test Variants			
		“Herbicide” (Reference) Group (2,4-D Only)	“Herbicide + Antidote” Group		
		Crop Yield, Quintals per Hectare	Crop Yield, Quintals per Hectare	Antidote Activity	
				Quintals per Hectare	*A_F_*, %
1	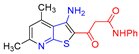 **22a**	13.8	20.2	6.4	46.4 ^1^
2	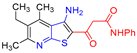 **22d**	13.8	20.3	6.5	47.1 ^1^
3	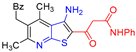 **22f**	13.8	21.7	7.9	57.2 ^1^

^1^ The differences are statistically significant at *p* = 0.90.

## Data Availability

File Electronic Supplementary Material.pdf containing X ray data, ^1^H and ^13^C DEPTQ NMR, FTIR spectral charts.

## Supplementary Materials

The following supporting information can be downloaded at: https://www.mdpi.com/article/10.3390/molecules31172975/s1.

## References

[1] Bakhite E.A.G. (2003). Recent trends in the chemistry of thienopyridines. Phosphorus Sulfur Silicon Relat. Elem..

[2] Litvinov V.P., Dotsenko V.V., Krivokolysko S.G. (2005). Thienopyridines: Synthesis, properties, and biological activity. Russ. Chem. Bull..

[3] Litvinov V.P., Dotsenko V.V., Krivokolysko S.G. (2007). The chemistry of thienopyridines. Adv. Heterocycl. Chem..

[4] Sajadikhah S.S., Marandi G. (2019). Recent approaches to the synthesis of thieno[2,3*-b*]pyridines (microreview). Chem. Heterocycl. Compd..

[5] Dotsenko V.V., Buryi D.S., Lukina D.Y., Krivokolysko S.G. (2020). Recent advances in the chemistry of thieno[2,3-b]pyridines 1. methods of synthesis of thieno[2,3*-b*]pyridines. Russ. Chem. Bull..

[6] Shaw R., Tewari R., Yadav M., Pandey E., Tripathi K., Rani J., Althagafi I., Pratap R. (2024). Recent advancements in the synthesis of fused thienopyridines and their therapeutic applications. Eur. J. Med. Chem. Rep..

[7] Stoikov I.I., Antipin I.S., Burilov V.A., Kurbangalieva A.R., Rostovskii N.V., Pankova A.S., Balova I.A., Remizov Y.O., Pevzner L.M., Petrov M.L. (2024). Organic chemistry in Russian universities. Achievements of recent years. Russ. J. Org. Chem..

[8] Charushin V.N., Verbitskiy E.V., Chupakhin O.N., Vorobyeva D.V., Gribanov P.S., Osipov S.N., Ivanov A.V., Martynovskaya S.V., Sagitova E.F., Dyachenko V.D. (2024). The chemistry of heterocycles in the 21st century. Russ. Chem. Rev..

[9] Mabrouk B.K.A., Zaki M.E.A., Abdallah M.A., Gomha S.M. (2025). Synthetic approaches and reactivity of 3-aminothieno[2,3-*b*]pyridine derivatives—A Review. Curr. Org. Chem..

[10] Salem M.A., Abu-Hashem A.A., Abdelgawad A.A.M., Gouda M.A. (2021). Synthesis and reactivity of thieno[2,3*-b*]quinoline derivatives (part II). J. Heterocycl. Chem..

[11] Stroganova T.A., Vasilin V.K., Dotsenko V.V., Aksenov N.A., Morozov P.G., Vassiliev P.M., Volynkin V.A., Krapivin G.D. (2021). Unusual oxidative dimerization in the 3-aminothieno[2,3*-b*]pyridine-2-carboxamide series. ACS Omega.

[12] Stroganova T.A., Vasilin V.K., Dotsenko V.V., Aksenov N.A., Krapivin G.D. (2019). Reaction of thieno[2,3*-b*]pyridines with sodium hypochlorite: An unusual and stereoselective one-pot approach to dimeric pyrrolo[2′,3′:4,5]thieno[2,3*-b*]pyridines. Tetrahedron Lett..

[13] Mohamed S.K., Siddique S.A., Sarfraz M., Bakhite E.A., Abuelhassan S., Marae I.S., Soliman A.A.E., Khamies E., Smerat A., Balboul B.A.A. (2026). Synthesis, structural, electronic, and medicinal characterization of a thiophene-bearing polycyclic compound: An approach to discover potential lead compounds for precision cancer therapy. ChemistrySelect.

[14] Al-Waleedy S.A.H., Orabi E.A., Bakhite E.A., Sharmoukh W., Younis O. (2026). From solvent polarity to molecular packing: Photophysical insights into newly synthesized pyridothienopyrimidinone derivatives. J. Photochem. Photobiol. A Chem..

[15] Barker J.M. (1977). The Thienopyridines. Adv. Heterocycl. Chem..

[16] Paronikyan E.G., Noravyan A.S., Vartanyan S.A. (1987). Synthesis, transformations, and pharmacological properties of thienopyridines (review). Pharm. Chem. J..

[17] Belyakova Y.Y., Yaremenko I.A., Terent’ev A.O., Nenajdenko V.G., Shambalova V.E., Aldoshin A.S., Krasnovskaya O.O., Beloglazkina E.K., Spektor D.V., Machulkin A.E. (2026). Organic chemistry in the creation of molecules with practically useful properties. Russ. J. Gen. Chem..

[18] Teja C., Nawaz Khan F.R. (2020). Recent advances in the synthesis of thienoquinolines (Quinoline-fused heterocycle). Asian J. Org. Chem..

[19] El-Sayed H.A. (2014). Heterocyclization of ethyl 3-amino-4,6-dimethylthieno[2,3*-b*]pyridine-2-carboxylate (review). J. Iran. Chem. Soc..

[20] Pinheiro L.C.S., Abreu P.A., Afonso I.F., Leal B., Corrêa L.C.D., Borges J.C., Marques I.P., Lourenço A.L., Sathler P., dos Santos A.L. (2008). Identification of a potential lead structure for designing new antimicrobials to treat infections caused by *Staphylococcus epidermidis*-resistant strains. Curr. Microbiol..

[21] Pinheiro L.C.S. (2012). Searching for new antileishmanial lead drug candidates: Synthesis, biological and theoretical evaluations of promising thieno[2,3*-b*]pyridine derivatives. J. Microbiol. Antimicrob..

[22] Amorim R., de Meneses M.D.F., Borges J.C., da Silva Pinheiro L.C., Caldas L.A., Cirne-Santos C.C., de Mello M.V.P., de Souza A.M.T., Castro H.C., de Palmer Paixão I.C.N. (2017). Thieno[2,3*-b*]pyridine derivatives: A new class of antiviral drugs against *Mayaro* virus. Arch. Virol..

[23] Boschelli D.H., Wu B., Barrios Sosa A.C., Chen J., Asselin M., Cole D.C., Lee J., Yang X., Chaudhary D. (2008). Synthesis and PKCθ inhibitory activity of a series of 4-(indol-5-ylamino)thieno[2,3*-b*]pyridine-5-carbonitriles. Bioorg. Med. Chem. Lett..

[24] Tumey L.N., Boschelli D.H., Lee J., Chaudhary D. (2008). 2-Alkenylthieno[2,3-*b*]pyridine-5-carbonitriles: Potent and selective inhibitors of PKCθ. Bioorg. Med. Chem. Lett..

[25] Wu B., Boschelli D.H., Lee J., Yang X., Chaudhary D. (2009). Second generation 4-(4-methyl-1H-indol-5-ylamino)- 2-phenylthieno[2,3*-b*]pyridine-5-carbonitrile PKCθ inhibitors. Bioorg. Med. Chem. Lett..

[26] Dotsenko V.V., Krivokolysko S.G., Chernega A.N., Litvinov V.P. (2002). Anilinomethylidene derivatives of cyclic 1,3-dicarbonyl compounds in the synthesis of new sulfur-containing pyridines and quinolines. Russ. Chem. Bull..

[27] Reynisson J., Court W., O’Neill C., Day J., Patterson L., McDonald E., Workman P., Katan M., Eccles S.A. (2009). The identification of novel PLC-γ inhibitors using virtual high throughput screening. Bioorg. Med. Chem..

[28] Leung E., Hung J.M., Barker D., Reynisson J. (2013). The effect of a thieno[2,3*-b*]pyridine PLC-γ inhibitor on the proliferation, morphology, migration and cell cycle of breast cancer cells. Med. Chem. Commun..

[29] Reynisson J., Jaiswal J.K., Barker D., D’mello S.A.N., Denny W.A., Baguley B.C., Leung E.Y. (2016). Evidence that phospholipase C is involved in the antitumour action of NSC768313, a new thieno[2,3*-b*]pyridine derivative. Cancer Cell Int..

[30] van Rensburg M., Leung E., Haverkate N.A., Eurtivong C., Pilkington L.I., Reynisson J., Barker D. (2017). Synthesis and antiproliferative activity of 2-chlorophenyl carboxamide thienopyridines. Bioorg. Med. Chem. Lett..

[31] Zafar A., Sari S., Leung E., Pilkington L., Van Rensburg M., Barker D., Reynisson J. (2017). GPCR modulation of thieno[2,3*-b*]pyridine anti-proliferative agents. Molecules.

[32] Binsaleh N.K., Wigley C.A., Whitehead K.A., van Rensburg M., Reynisson J., Pilkington L.I., Barker D., Jones S., Dempsey-Hibbert N.C. (2018). Thieno[2,3*-b*]pyridine derivatives are potent anti-platelet drugs, inhibiting platelet activation, aggregation and showing synergy with aspirin. Eur. J. Med. Chem..

[33] Schweda S.I., Alder A., Gilberger T., Kunick C. (2020). 4-Arylthieno[2,3*-b*]pyridine-2-carboxamides are a new class of antiplasmodial agents. Molecules.

[34] Masch A., Nasereddin A., Alder A., Bird M.J., Schweda S.I., Preu L., Doerig C., Dzikowski R., Gilberger T.W., Kunick C. (2019). Structure–activity relationships in a series of antiplasmodial thieno[2,3*-b*]pyridines. Malar. J..

[35] Masch A., Kunick C. (2015). Selective inhibitors of *Plasmodium falciparum* glycogen synthase-3 (PfGSK-3): New antimalarial agents?. Biochim. Biophys. Acta (BBA)-Proteins Proteom..

[36] Fugel W., Oberholzer A.E., Gschloessl B., Dzikowski R., Pressburger N., Preu L., Pearl L.H., Baratte B., Ratin M., Okun I. (2013). 3,6-Diamino-4-(2-halophenyl)-2-benzoylthieno[2,3*-b*]pyridine-5-carbonitriles are selective inhibitors of *Plasmodium falciparum* glycogen synthase kinase-3. J. Med. Chem..

[37] Bonafoux D., Lee W.C. (2009). Strategies for TGF-β modulation: A review of recent patents. Expert Opin. Ther. Pat..

[38] Gilmour R., Foster J.E., Sheng Q., McClain J.R., Riley A., Sun P.M., Ng W.L., Yan D., Nicas T.I., Henry K. (2005). New class of competitive inhibitor of bacterial histidine kinases. J. Bacteriol..

[39] Anighoro A., Pinzi L., Marverti G., Bajorath J., Rastelli G. (2017). Heat shock protein 90 and serine/threonine kinase B-raf inhibitors have overlapping chemical space. RSC Adv..

[40] May B.C.H., Zorn J.A., Witkop J., Sherrill J., Wallace A.C., Legname G., Prusiner S.B., Cohen F.E. (2007). Structure−activity relationship study of prion inhibition by 2-aminopyridine-3,5-dicarbonitrile-based compounds:  parallel synthesis, bioactivity, and in vitro pharmacokinetics. J. Med. Chem..

[41] Saito K., Nakao A., Shinozuka T., Shimada K., Matsui S., Oizumi K., Yano K., Ohata K., Nakai D., Nagai Y. (2013). Discovery and structure–activity relationship of thienopyridine derivatives as bone anabolic agents. Bioorg. Med. Chem..

[42] Saito K., Shinozuka T., Nakao A., Kiho T., Kunikata T., Shiiki T., Nagai Y., Naito S. (2019). Synthesis and structure-activity relationship of 4-alkoxy-thieno[2,3*-b*]pyridine derivatives as potent alkaline phosphatase enhancers for osteoporosis treatment. Bioorg. Med. Chem. Lett..

[43] Antczak M.I., Zhang Y., Wang C., Doran J., Naidoo J., Voruganti S., Williams N.S., Markowitz S.D., Ready J.M. (2017). Inhibitors of 15-prostaglandin dehydrogenase to potentiate tissue repair. J. Med. Chem..

[44] Nagarajan S., Doddareddy M., Choo H., Cho Y.S., Oh K.S., Lee B.H., Pae A.N. (2009). IKKβ inhibitors identification part I: Homology model assisted structure based virtual screening. Bioorg. Med. Chem..

[45] Mermerian A.H., Case A., Stein R.L., Cuny G.D. (2007). Structure–activity relationship, kinetic mechanism, and selectivity for a new class of ubiquitin C-terminal hydrolase-L1 (UCH-L1) inhibitors. Bioorg. Med. Chem. Lett..

[46] Dayam R., Al-Mawsawi L.Q., Zawahir Z., Witvrouw M., Debyser Z., Neamati N. (2008). Quinolone 3-carboxylic acid pharmacophore: Design of second generation HIV-1 integrase inhibitors. J. Med. Chem..

[47] Bakhite E.A., Gad M.A., Khamies E., Thagfan F.A., Mohamed R.A.E.H., Bakry M.M.S. (2025). Exploration of some thieno[2,3-*b*]pyridines, thieno[3,2*-d*]pyrimidinones, and thieno[3,2*-d*][1,2,3]triazinones as insecticidal agents against *Aonidiella aurantii*. Russ. J. Bioorg. Chem..

[48] Gad M.A., Bakry M.M.S., Tolba E.F.M., Alkhaibari A.M., Mashlawi A.M., Thabet M.A., Al-Taifi E.A., Bakhite E.A. (2024). Exploration of some heterocyclic compounds containing trifluoromethylpyridine scaffold as insecticides toward *Aphis gossypii* insects. Chem. Biodivers..

[49] Khamies E., El-Emary T.I., Said A.I., Gad M.A., Abdel-Hafez S.H., Marae I.S., Soliman A.A.E., Bakhite E.A. (2025). Pyridine derivatives as Insecticides. part 7. Synthesis, characterization and insecticidal activity of some new 1-amino-n-substituted-6,7,8,9-tetrahydrothieno[2,3*-c*]isoquinoline-2-carboxamides and their 1-(1-pyrrolyl) analogues. J. Agric. Food Chem..

[50] Andrianova O.A., Buryi D.S., Gluzmin N.O., Daus E.S., Dotsenko V.V., Kindop V.K., Kindop V.K., Kosenko D.D., Strelkov V.D., Tsymbal T.L. (2025). Synthesis and biological activity of new phenothiazine-based heterodimers. Russ. Chem. Bull..

[51] Kindop V.K., Bespalov A.V., Dotsenko V.V., Strelkov V.D., Lukina D.Y., Baichurin R.I., Paronikyan E.G., Harutyunyan A.S., Ovcharov S.N., Aksenov N.A. (2025). Synthesis and structural characterization of 2-iminothiazoline/quinoline and 2-iminothiazoline/thieno[2,3*-b*]quinoline molecular hybrids with herbicide safening properties. Tetrahedron.

[52] Dotsenko V.V., Muraviev V.S., Lukina D.Y., Strelkov V.D., Aksenov N.A., Aksenova I.V., Krapivin G.D., Dyadyuchenko L.V. (2020). Reaction of 3-Amino-4,6-diarylthieno[2,3*-b*]pyridine-2-carboxamides with Ninhydrin. Russ. J. Gen. Chem..

[53] Dotsenko V.V., Buryi D.S., Lukina D.Y., Stolyarova A.N., Aksenov N.A., Aksenova I.V., Strelkov V.D., Dyadyuchenko L.V. (2019). Substituted *N*-(thieno[2,3*-b*]pyridine-3-yl)acetamides: Synthesis, reactions, and biological activity. Monatsh Chem..

[54] Kyarov R.V., Popov P.A., Levchenko A.G., Dahno P.G., Tsymbal T.L., Daus E.S., Hagur T.R., Khomyakova A.A., Abramova A.E., Katyshkov N.A. (2026). Synthesis and properties of 3-amino-4-(4-methoxyphenyl)-6-oxo-4,5,6,7- tetrahydrothieno[2,3*-b*]pyridines. Russ. J. Gen. Chem..

[55] Dotsenko V.V., Rudenko S.V., Lukina D.Y., Smirnova A.K., Krivokolysko S.G., Temerdashev A.Z., Harutyunyan A.S., Paronikyan E.G., Aksenov N.A., Aksenova I.V. (2025). Synthesis of 2,2-dimethyl-2,3-dihydropyrido[3′,2′:4,5]thieno[3,2*- d*]pyrimidin-4(1*H*)-ones by reaction of 3-aminothieno[2,3*-b*]pyridine-2-carboxamides with acetone. Russ. J. Gen. Chem..

[56] Buryi D.S., Dotsenko V.V., Aksenov N.A., Aksenova I.V., Krivokolysko S.G., Dyadyuchenko L.V. (2019). Synthesis and properties of 4,6-dimethyl-5-pentyl-2-thioxo-1,2-dihydropyridine-3-carbonitrile and 3-amino-4,6-dimethyl-5-pentylthieno[2,3*- b*]pyridines. Russ. J. Gen. Chem..

[57] Bakhite E.A., Ibrahim O.F., Khamies E., Abdel-Hafez S.H., Qahtan M.Q.M., Marae I.S. (2024). Synthesis, characterization and biological activity of some new dihydroisoquinolines and dihydrothieno[2,3*-c*]isoquinolines. Arkivoc.

[58] Wu X., Li W. (2022). The applications of β-keto amides for heterocycle synthesis. J. Heterocycl. Chem..

[59] Li W., Zheng Y., Qu E., Bai J., Deng Q. (2021). β-Keto amides: A jack-of-all-trades building block in organic chemistry. Eur. J. Org. Chem..

[60] Fadda A.A., Abdel-Galil E., Elattar K.M. (2015). Chemistry of 3-oxo-N-(pyridin-2-yl)butanamide and related compounds. Synth. Commun..

[61] Hynes M.J., Clarke E.M. (1998). Reactions of metal ions with β-ketoamides. J. Chem. Soc. Perkin Trans. 2.

[62] Zayed M.A. (2023). Studies on the formation and composition of copper(II)-α- and γ-halo-1-oxobutanilides complexes. J. Transit. Met. Complexes.

[63] Göhmann P., Schröder L., Zschunke A. (1985). Bromierung von Acetessigsäureaniliden und Folgereaktionen. Z. Chem..

[64] Dyachenko V.D., Dyachenko I.V., Nenajdenko V.G. (2018). Cyanothioacetamide: A polyfunctional reagent with broad synthetic utility. Russ. Chem. Rev..

[65] Frolova N.G., Zav’yalova V.K., Litvinov V.P. (1996). Synthesis of 4,5,6-trisubstituted 3-cyanopyridine-2(1*H*)-thiones based on α-substituted β-diketones. Russ. Chem. Bull..

[66] Dakhno P.G., Kindop V.K., Gordeev K.V., Zimmer I.A., Dotsenko V.V., Temerdashev A.Z., Vasilin V.K., Aksenov N.A., Aksenova I.V. (2023). Oxidation of 4,6-dimethyl-2-thioxo-1,2-dihydropyridine-3-carbonitriles with potassium ferricyanide: Synthesis and molecular docking of bis(pyrid-2-yl)disulfides. Russ. J. Gen. Chem..

[67] Narushyavichus É.V., Garalene V.N., Krauze A.A., Dubur G.Y. (1989). Cardiotropic activity of pyridin-2(1*H*)-ones. Pharm. Chem. J..

[68] Wagner G., Vieweg H., Leistner S., Böhm N., Krasselt U., Hanfeld V., Prantz J., Grupe R. (1990). Synthese neuer prim., sek. und tert. 3-Amino-thieno[2,3*-b*]pyridin-2-carbonsäureamide auf verschiedenen Wegen. Pharmazie.

[69] Kaigorodova E.A., Konyushkin L.D., Niyazymbetov M.E., Kvak S.N., Zaplishny V.N., Litvinov V.P. (1994). Electrochemical synthesis and studies of substituted 2-thiopyridines. Russ. Chem. Bull..

[70] Dotsenko V.V., Chigorina E.A., Papaianina E.S., Frolov K.A., Krivokolysko S.G. (2015). The Mannich-type Synthesis of macroheterocycles with pyrido[2,1*-b*][1,3,5]thiadiazine unit. Macroheterocycles.

[71] Nesterov V.N., Krivokolysko S.G., Dotsenko V.V., Litvinov V.P. (1997). Synthesis, properties, and structures of ammonium 4-aryl-5-cyano-2-oxo-1,2,3,4-tetrahydropyridine-6-thiolates. Russ. Chem. Bull..

[72] Dotsenko V.V., Frolov K.A., Pekhtereva T.M., Papaianina O.S., Suykov S.Y., Krivokolysko S.G. (2014). Design and synthesis of pyrido[2,1*-b*][1,3,5]thiadiazine library via uncatalyzed Mannich-type Reaction. ACS Comb. Sci..

[73] Sangshetti J., Zambare A., Khan F., Gonjari I., Zaheer Z. (2014). Synthesis and biological activity of substituted-4,5,6,7-tetrahydrothieno pyridines: A Review. Mini-Rev. Med. Chem..

[74] Dou N., Ma H., Zhang P., Lu R., Hang J., Sun J. (2025). Advances in the clinical use of Clopidogrel: A review of individualized treatment strategies and monitoring optimization based on genetic polymorphisms. Pharmacogenomics Pers. Med..

[75] Patti G., Micieli G., Cimminiello C., Bolognese L. (2020). The role of clopidogrel in 2020: A Reappraisal. Cardiovasc. Ther..

[76] Plosker G.L., Lyseng-Williamson K.A. (2007). Clopidogrel. Drugs.

[77] Zhao X., Ma S., Kang Y., Tang C., Liu B., Jiang H., Zheng M., Tang Y., Sun H., Liu Y. (2022). Antiplatelet effect, safety, and pharmacokinetics of vicagrel in patients with coronary artery disease undergoing percutaneous coronary intervention. Eur. Heart J.-Cardiovasc. Pharmacother..

[78] Freeman M.K. (2010). Thienopyridine antiplatelet agents: Focus on Prasugrel. Consult. Pharm..

[79] da Cruz R.M.D., Mendonça-Junior F.J.B., de Mélo N.B., Scotti L., de Araújo R.S.A., de Almeida R.N., de Moura R.O. (2021). Thiophene-based compounds with potential anti-inflammatory activity. Pharmaceuticals.

[80] Kazuhiro G., Masao H., Hiroshi I. (1977). Effects of tinoridine on stability of rat liver and kidney lysosomes, and liver parenchymal cells. Biochem. Pharmacol..

[81] Bernardo V.G., de Araújo R.S.A., de Mélo N.B., Coutinho R.E.P., de Sousa J.M.S., da Silva Sousa M.G.G., de Sousa N.F., dos Santos Silva W.F., da Silva Santos-Júnior P.F., da Silva T.G. (2026). Drug design and synthesis of new N-substituted-thienopyridine based on 2-aminothiophene derivatives as antileishmanial agents. Bioorg. Med. Chem..

[82] Dotsenko V.V., Lukina D.Y., Buryi D.S., Strelkov V.D., Aksenov N.A., Aksenova I.V. (2021). Synthesis of new polycyclic compounds containing thieno[2′,3′:5,6]pyrimido[2,1*-a*]isoindole fragment. Russ. J. Gen. Chem..

[83] Dotsenko V.V., Krivokolysko S.G., Chernega A.N., Litvinov V.P. (2003). Fused sulfur-containing pyridine systems. 1. Synthesis and structures of tetrahydropyridothienopyridinone and tetrahydropyridothiopyranopyridinone derivatives. Russ. Chem. Bull..

[84] Dotsenko V.V., Krivokolysko S.G., Litvinov V.P., Chernega A.N. (2002). First multicomponent synthesis of tricyclic hydrogenated pyridines. Russ. Chem. Bull..

[85] Dyachenko V.D., Krivokolysko S.G., Litvinov V.P. (1997). A new method for the synthesis of *N*-methylmorpholinium 4-aryl-5-cyano-2-oxo-1,2,3,4-tetrahydropyridine-6-thiolates and their properties. Russ. Chem. Bull..

[86] Dotsenko V.V., Krivokolysko S.G., Litvinov V.P. (2003). Synthesis of hexa- and octahydropyrido[3′,2′:4,5]thieno[3,2*-d*]pyrimidines. Chem. Heterocycl. Compd..

[87] Dotsenko V.V., Krivokolysko S.G., Litvinov V.P. (2004). A novel approach to the synthesis of partially hydrogenated dipyridothiophenes. Mendeleev Commun..

[88] Wang Y., Duraiswami C., Madauss K.P., Tran T.B., Williams S.P., Deng S.J., Graybill T.L., Hammond M., Jones D.G., Grygielko E.T. (2009). 2-Amino-9-aryl-3-cyano-4-methyl-7-oxo-6,7,8,9-tetrahydropyrido[2′,3′: 4,5]thieno[2,3*-b*]pyridine derivatives as selective progesterone receptor agonists. Bioorg. Med. Chem. Lett..

[89] Wang W., Lv D., Qiu N., Zhang L., Hu C., Hu Y. (2013). Design, synthesis and biological evaluation of novel 3,4,5-trisubstituted aminothiophenes as inhibitors of p53–MDM2 interaction. part 2. Bioorg. Med. Chem..

[90] Angell R.M., Atkinson F.L., Brown M.J., Chuang T.T., Christopher J.A., Cichy-Knight M., Dunn A.K., Hightower K.E., Malkakorpi S., Musgrave J.R. (2007). N-(3-Cyano-4,5,6,7-tetrahydro-1-benzothien-2-yl)amides as potent, selective, inhibitors of JNK2 and JNK3. Bioorg. Med. Chem. Lett..

[91] Kaminka M., Vorob’eva V.Y., Nikitin V.B., Mikhlina E.E., Zhirnikova M.L., Yakhontov L.N. (1987). Synthesis and properties of 2-amino-3-ethycarbonylthieno[2,3*-b*]quinuclidines. Pharm. Chem. J..

[92] Rodinovskaya L.A., Shestopalov A.M. (2000). Synthesis of substituted 4-hydroxy-1*H*-thieno[2,3*-b*;4,5*-b*′]dipyridin-2-ones. Russ. Chem. Bull..

[93] Shestopalov A.M., Nikishin K.G., Gromova A.V., Rodinovskaya L.A. (2003). One-pot synthesis of 4,6-diaryl-3-cyanopyridine- 2(1*H*)-thiones and their transformation to substituted thieno[2,3*-b*;4,5*-b*]dipyridines and pyrido[3”,2”:4,5]thieno[3,2*-d*]pyrimidines. Russ. Chem. Bull..

[94] Shestopalov A.A., Gromova A.V., Rodinovskaya L.A., Nikishin K.G., Litvinov V.P., Shestopalov A.M. (2004). One-step synthesis of 3-cyano-6-methyl-4-thienyl-5,6,7,8-tetrahydro[1,6]naphthyridine-2(1*H*)-thiones and annelated heterocyclic systems on their basis. Russ. Chem. Bull..

[95] Rodinovskaya L., Shestopalov A., Gromova A., Shestopalov A. (2006). Substituted 4-(3-cyanopyridin-2-ylthio)acetoacetates: New convenient reagents for the synthesis of heterocycles. Synthesis.

[96] Rodinovskaya L.A., Fedorov A.E., Shestopalov A.M., Belyakov P.A., Nikishin K.G. (2013). Synthesis of annulated heterocyclic systems based on 4-CF_3_- or 4-CHF_2_-3-cyano-(1*H*)-pyridine-2-thiones. Russ. Chem. Bull..

[97] Shestopalov A.M., Rodinovskaya L.A., Zubarev A.A., Nesterov V.N., Ugrak B.I., Dutova T.Y. (2020). Synthesis and domino reactions of polymethylene-3-cyanopyridine-2(1*H*)-thiones. J. Heterocycl. Chem..

[98] Neese F. (2012). The ORCA program system. WIREs Comput. Mol. Sci..

[99] Neese F. (2022). Software update: The ORCA program system—Version 5.0. WIREs Comput. Mol. Sci..

[100] Becke A.D. (2002). Density-functional exchange-energy approximation with correct asymptotic behavior. Phys. Rev. A.

[101] Lee C., Yang W., Parr R.G. (2002). Development of the Colle-Salvetti correlation-energy formula into a functional of the electron density. Phys. Rev. B.

[102] Grimme S., Ehrlich S., Goerigk L. (2011). Effect of the damping function in dispersion corrected density functional theory. J. Comput. Chem..

[103] Tomasi J., Mennucci B., Cammi R. (2005). Quantum mechanical continuum solvation models. Chem. Rev..

[104] Andersson M.P., Uvdal P. (2005). New scale factors for harmonic vibrational frequencies using the B3LYP density functional method with the Triple-ζ basis Set 6-311+G(d,p). J. Phys. Chem. A.

[105] Peterson M.A., McMaster S.A., Riechers D.E., Skelton J., Stahlman P.W. (2016). 2,4-D past, present, and future: A review. Weed Technol..

[106] Hoffmann O.L. (1953). Inhibition of auxin effects by 2,4,6-trichlorophenoxyacetic acid. Plant Physiol..

[107] Chkanikov N.D., Spiridonov Y.Y., Khalikov S.S., Muzafarova A.M. (2019). Antidotes for reduction of phytotoxicity of the residues of sulfonylurea herbicides. INEOS OPEN.

[108] Deng X. (2022). Current advances in the action mechanisms of safeners. Agronomy.

[109] Jia L., Jin X.Y., Zhao L.X., Fu Y., Ye F. (2022). Research progress in the design and synthesis of herbicide safeners: A review. J. Agric. Food Chem..

[110] Deng X. (2022). A mini review on natural safeners: Chemistry, uses, modes of action, and limitations. Plants.

[111] Zhao Y., Ye F., Fu Y. (2024). Herbicide Safeners: From molecular structure design to safener activity. J. Agric. Food Chem..

[112] Leng X.Y., Zhao L.X., Gao S., Ye F., Fu Y. (2023). Review on the discovery of novel natural herbicide safeners. J. Agric. Food Chem..

[113] Deng X., Wu Y., Bai L. (2026). Advancing herbicide safeners: Integrating structure-guided design, pathway mechanism elucidation, and smart delivery. Adv. Agrochem.

[114] Dotsenko V.V., Sinotsko A.E., Strelkov V.D., Varzieva E.A., Russkikh A.A., Levchenko A.G., Temerdashev A.Z., Aksenov N.A., Aksenova I.V. (2023). Alkyl 4-aryl-6-amino-7-phenyl-3-(phenylimino)-4,7-dihydro-3*H*-[1,2]dithiolo[3,4*-b*]pyridine- 5-carboxylates: Synthesis and agrochemical Studies. Molecules.

[115] Dolomanov O.V., Bourhis L.J., Gildea R.J., Howard J.A.K., Puschmann H. (2009). OLEX2: A complete structure solution, refinement and analysis program. J. Appl. Crystallogr..

[116] Sheldrick G.M. (2015). SHELXT–integrated space-group and crystal-structure determination. Acta Crystallogr. Sect. A.

[117] Sheldrick G.M. (2015). Crystal structure refinement with SHELXL. Acta Crystallogr. Sect. C.

