# Supplementary material for: β-Ketoamides in Thieno[2,3-b]pyridine Series: Synthesis, Quantum Chemical Studies and 2,4-D Herbicide Safening Effects
Source: Molecules. 2026 Aug 25;31(17):2975. doi: 10.3390/molecules31172975 (PMC13567366; doi:10.3390/molecules31172975)

Supporting Information File

β-Ketoamides in thieno[2,3-*b*]pyridine series: synthesis, quantum chemical studies and 2,4-D herbicide safening effects

***Artem A. Amirkhanyan*[1], *Victor V. Dotsenko*[1,2]*, *Alexander V. Bespalov*[1], *Tatyana R. Hagur*[1], *Eva S. Daus*[1], *Igor V. Yudaev*[3], *Yulia V. Daus*[3], *Darya Yu. Lukina*[1], *Vladimir K. Vasilin*[4], *Nicolai A. Aksenov*[2], *Inna V. Aksenova*[2]**

[1]Department of Organic Chemistry and Technologies, Kuban State University, 149 Stavropolskaya St., 350040 Krasnodar, Russia E-mail: victor_dotsenko_@mail.ru
[2]Department of Chemistry, North Caucasus Federal University, 1a Pushkin St., 355017 Stavropol, Russia
[3]Faculty of Energetics, Kuban State Agrarian University, 13 Kalinina St., 350044 Krasnodar, Russia
[4]Department of Bioorganic Chemistry, Kuban State Technological University, 2 Moskovskaya St., 350072 Krasnodar, Russia

**Correspondence: victor_dotsenko_@mail.ru (V.V.D.).*

Contents. Selected spectral data

FT-IR spectrum (ATR mode) of thienopyridine 22a 4

^{1}H NMR spectrum (400 MHz, DMSO-d_6) of thienopyridine 22a 4

^{13}C DEPTQ NMR spectrum (101 MHz, DMSO-d_6) of thienopyridine 22a 5

X-ray data for 4-[(3-cyano-4,6-dimethylpyridin-2-yl)thio]-3-oxo-N- phenylbutanamide 22a 5

Table S1. Crystal data and structure refinement for 4-[(3-cyano-4,6-dimethylpyridin-2-yl)thio]-3-oxo-*N*-phenylbutanamide 22a 6

Table S2. Fractional atomic coordinates ($\times10^4$) and equivalent isotropic displacement parameters ($Å^2\times10^3$) for 22a 7

Table S3. Anisotropic displacement parameters ($Å^2\times10^3$) for thienopyridine 22a 8

Table S4. Bond lengths for thienopyridine 22a 9

Table S5. Bond angles for thienopyridine 22a 9

Table S6. Torsion angles for thienopyridine 22a 10

Table S7. Hydrogen atom coordinates ($Å\times10^4$) and isotropic displacement parameters ($Å^2\times10^3$) for thienopyridine 22a. 10

Table S8. Atomic occupancy for thienopyridine 22a 11

Experimental FT-IR spectrum (ATR mode) of thienopyridine 22a 12

IR spectrum of the most stable conformer K1 of thienopyridine 22a calculated at the B3LYP-D3BJ/6-311+G(2d,p) level of theory 12

FT-IR spectrum (ATR mode) of thienopyridine 22b 13

^{1}H NMR spectrum (400 MHz, DMSO-d_6) of thienopyridine 22b 13

^{13}C DEPTQ NMR spectrum (101 MHz, DMSO-d_6) of thienopyridine 22b 14

FT-IR spectrum (ATR mode) of thienopyridine 22c 14

^{1}H NMR spectrum (400 MHz, DMSO-d_6) of thienopyridine 22c 15

^{13}C NMR spectrum (101 MHz, DMSO-d_6) of thienopyridine 22c 15

^{1}H–^{13}C HSQC NMR spectrum (400/101 MHz, DMSO-d_6) of thienopyridine 22c 16

^{1}H–^{13}C HMBC NMR spectrum (400/101 MHz, DMSO-d_6) of thienopyridine 22c 18

Table S9. The observed correlations in the ^{1}H-^{13}C HSQC and ^{1}H-^{13}C HMBC 2D NMR spectra (400/101 MHz, DMSO-d_6) of thienopyridine 22c 20

FT-IR spectrum (ATR mode) of thienopyridine 22d 21

^{1}H NMR spectrum (400 MHz, DMSO-d_6) of thienopyridine 22d 21

^{13}C DEPTQ NMR spectrum (101 MHz, DMSO-d_6) of thienopyridine 22d 22

FT-IR spectrum (ATR mode) of thienopyridine 22e 22

^{1}H NMR spectrum (400 MHz, DMSO-d_6) of thienopyridine 22e 23

^{13}C DEPTQ NMR spectrum (101 MHz, DMSO-d_6) of thienopyridine 22e 23

FT-IR spectrum (ATR mode) of thienopyridine 22f 24

^{1}H NMR spectrum (400 MHz, DMSO-d_6) of thienopyridine 22f 24

^{13}C DEPTQ NMR spectrum (101 MHz, DMSO-d_6) of thienopyridine 22f 25

^{1}H–^{13}C HMBC NMR spectrum (400/101 MHz, DMSO-d_6) of thienopyridine 22f 25

Table S10. The observed correlations in the ^{1}H-^{13}C HMBC 2D NMR spectrum (400/101 MHz, DMSO-d_6) of thienopyridine 22f 27
^{1}H NMR spectrum (400 MHz, DMSO-d_6) of thienopyridine 22g 28
^{13}C DEPTQ NMR spectrum (101 MHz, DMSO-d_6) of thienopyridine 22g 28
FT-IR spectrum (ATR mode) of thienopyridine 22h 29
^{1}H NMR spectrum (400 MHz, DMSO-d_6) of thienopyridine 22h 29
^{13}C DEPTQ NMR spectrum (101 MHz, DMSO-d_6) of thienopyridine 22h 30
FT-IR spectrum (ATR mode) of thienopyridine 23 30
^{1}H NMR spectrum (400 MHz, DMSO-d_6) of thienopyridine 23 31
^{13}C DEPTQ NMR spectrum (101 MHz, DMSO-d_6) of thienopyridine 23 31
FT-IR spectrum (Nujol mulls) of the crude ketoamide intermediate 20d isolated 32
^{1}H NMR spectrum (400 MHz, DMSO-d_6) of the crude ketoamide intermediate 20d isolated 32
^{13}C DEPTQ NMR spectrum (101 MHz, DMSO-d_6) of the crude intermediate 20d isolated 33
FT-IR spectrum (Nujol mulls) of 4,5,6,7-tetrahyrdothieno[2,3-b]pyridine 24a 33
^{1}H NMR spectrum (400 MHz, DMSO-d_6) of 4,5,6,7-tetrahyrdothieno[2,3-b]pyridine 24a 34
^{13}C NMR spectrum (101 MHz, DMSO-d_6) of 4,5,6,7-tetrahyrdothieno[2,3-b]pyridine 24a 34
FT-IR spectrum (Nujol mulls) of 4,5,6,7-tetrahyrdothieno[2,3-b]pyridine 24b 35
^{1}H NMR spectrum (400 MHz, DMSO-d_6) of 4,5,6,7-tetrahyrdothieno[2,3-b]pyridine 24b 35
^{13}C NMR spectrum (101 MHz, DMSO-d_6) of 4,5,6,7-tetrahyrdothieno[2,3-b]pyridine 24b 36
FT-IR spectrum (Nujol mulls) of thiazolyl acetanilide 25 36
^{1}H NMR spectrum (400 MHz, DMSO-d_6) of thiazolyl acetanilide 25 37
^{13}C DEPTQ NMR spectrum (101 MHz, DMSO-d_6) of thiazolyl acetanilide 25 37
^{1}H NMR spectrum (400 MHz, DMSO-d_6) of the mixture product prepared by reaction of thienopyridine β-ketoamide 22a with triethyl orthoformate. Signals of dipyridothiophene 33 are highlighted with red circles; signals of the starting 22a are marked with green diamonds 38
^{1}H NMR spectrum (400 MHz, DMSO-d_6) of 4-hydroxy-7,9-dimethylthieno[2,3-*b*:4,5-*b*']dipyridine-2(1*H*)-one 33 38
^{13}C DEPTQ NMR spectrum (101 MHz, DMSO-d_6) of 4-hydroxy-7,9-dimethylthieno[2,3-*b*:4,5-*b*']dipyridine-2(1*H*)-one 33 39
FT-IR spectrum (ATR mode) of 4-hydroxy-7,9-dimethylthieno[2,3-*b*:4,5-*b*']dipyridine-2(1*H*)-one 33 39

FT-IR spectrum (ATR mode) of thienopyridine 22a

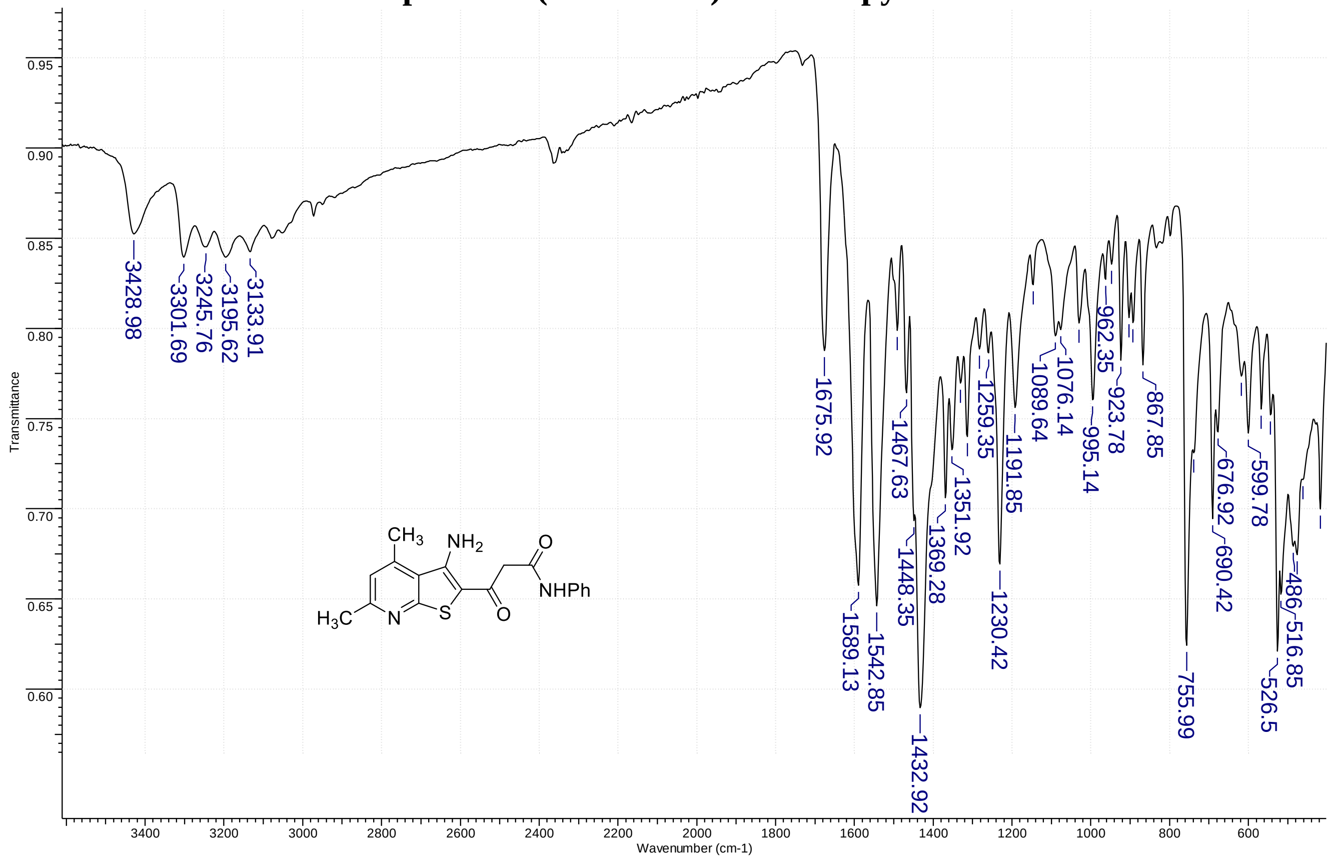

^{1}H NMR spectrum (400 MHz, DMSO-d$_6$) of thienopyridine 22a

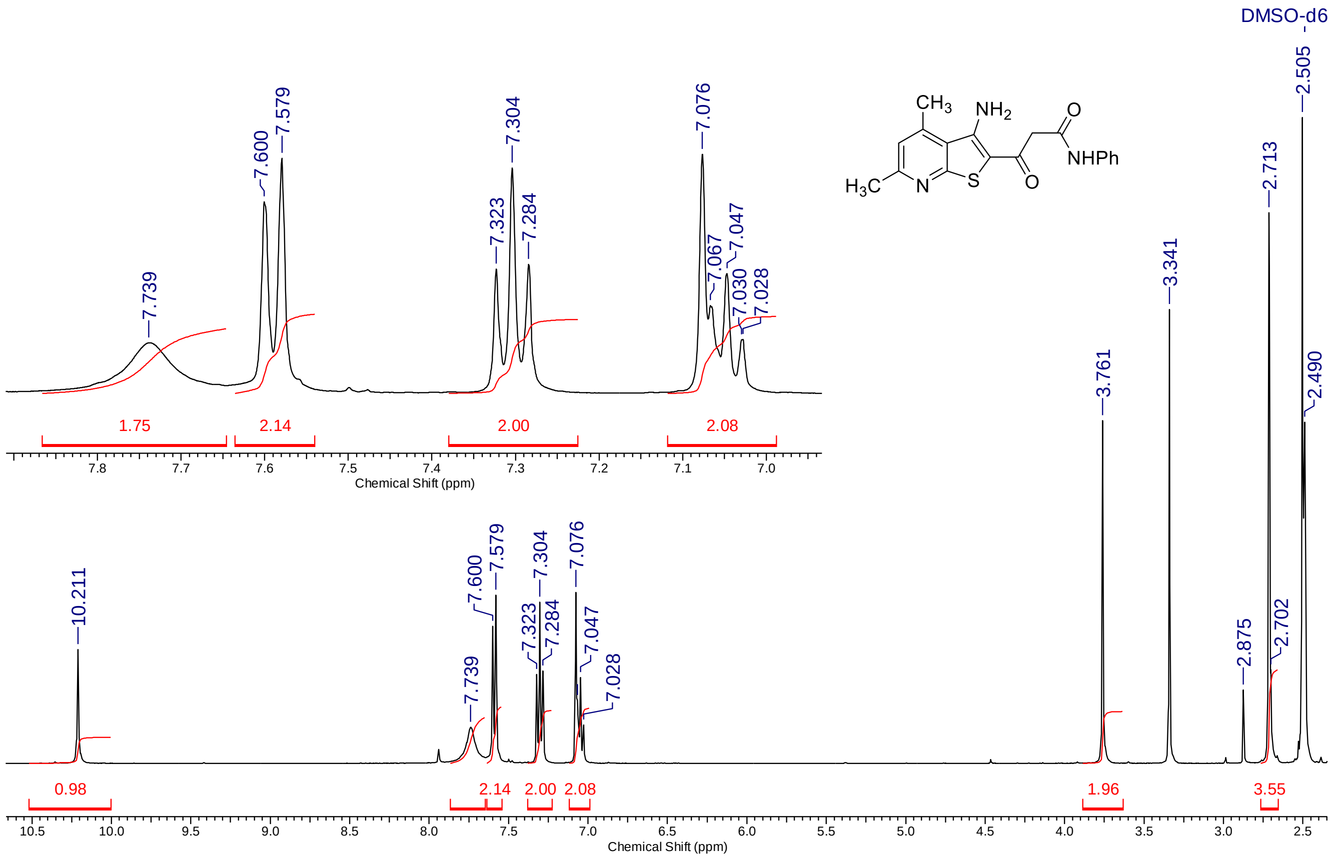

^{13}C DEPTQ NMR spectrum (101 MHz, DMSO-d_6) of thienopyridine 22a

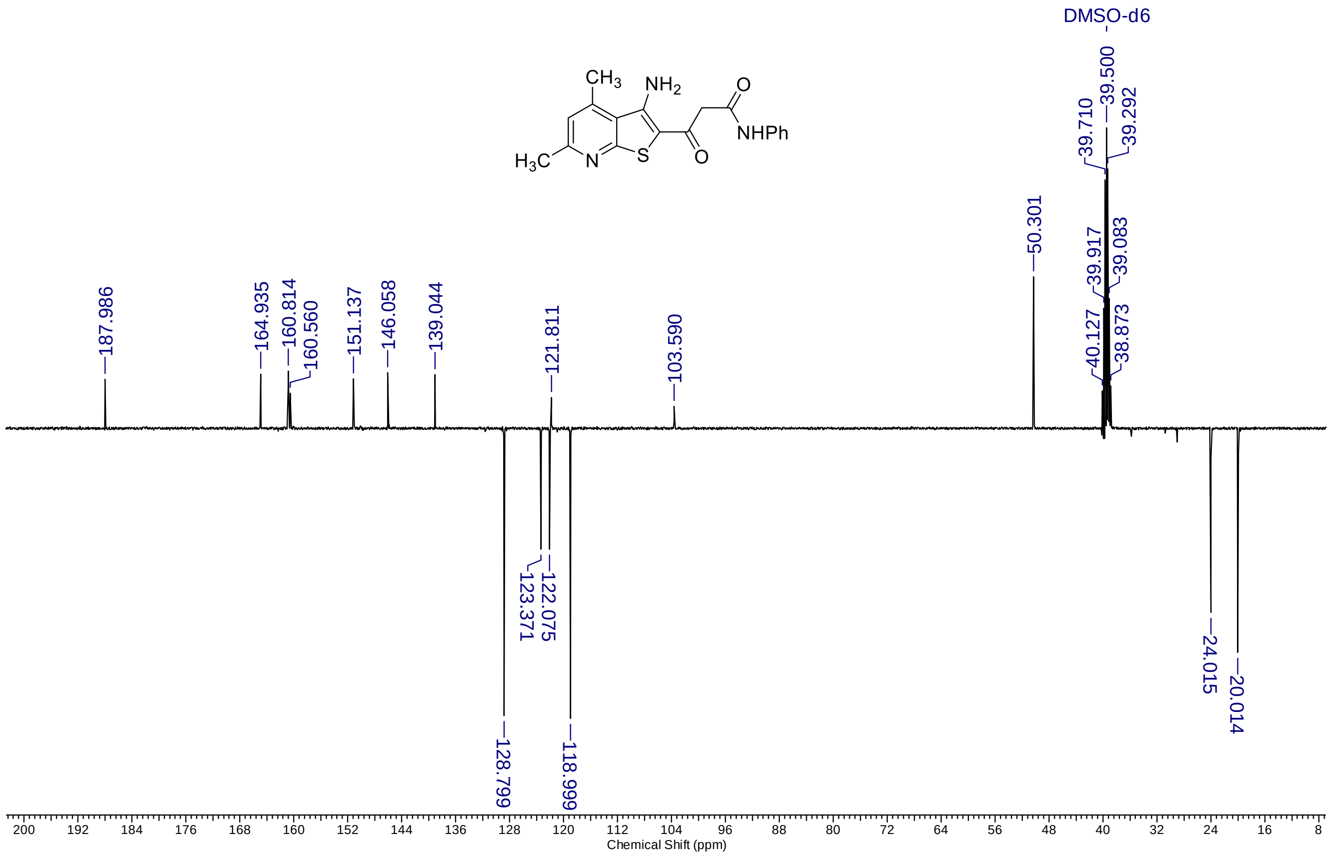

X-ray data for 4-[(3-cyano-4,6-dimethylpyridin-2-yl)thio]-3-oxo-N- phenylbutanamide 22a

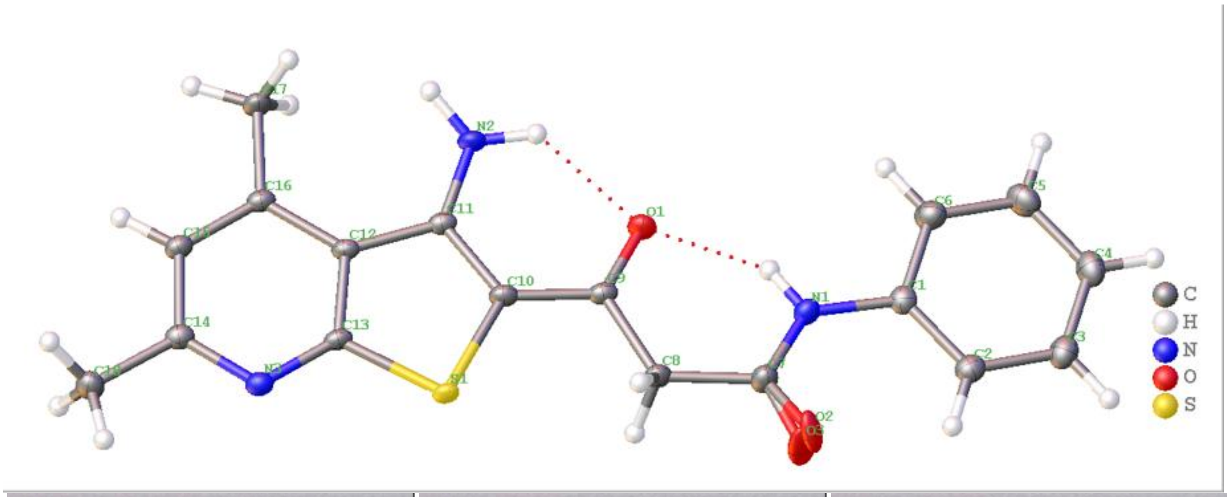

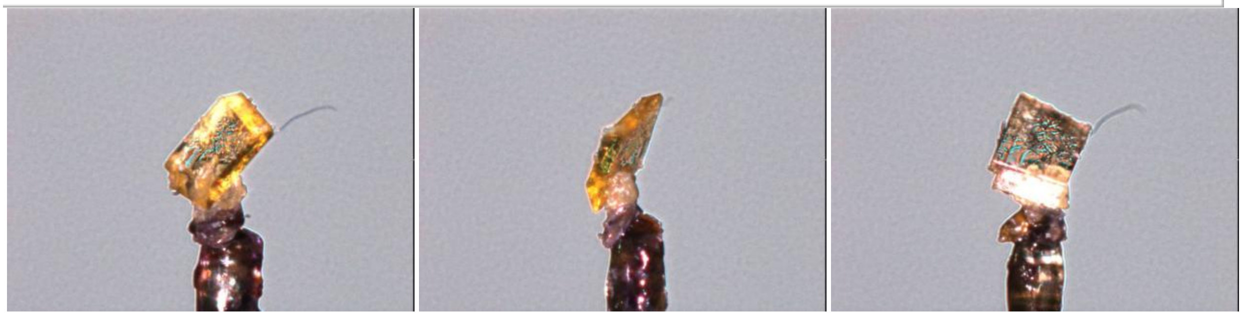

Table S1. Crystal data and structure refinement for 4-[(3-cyano-4,6-dimethylpyridin-2-yl)thio]-3-oxo-*N*-phenylbutanamide 22a.

Identification code	**22a** (ANNA_aaa98_2)
Empirical formula	$C_{18}H_{17}N_3O_2S$
Formula weight	339.40
Temperature/K	99.9(2)
Crystal system	orthorhombic
Space group	$Pca2_1$
a/Å	21.46758(18)
b/Å	5.33292(4)
c/Å	13.78236(14)
α/°	90
β/°	90
γ/°	90
Volume/Å^3	1577.87(2)
Z	4
ρ_{calc}g/cm^3	1.429
μ/mm^{-1}	1.959
F(000)	712.0
Crystal size/mm^3	0.34 × 0.24 × 0.06
Radiation	Cu Kα (λ = 1.54184)
2Θ range for data collection/°	8.238 to 152.524
Index ranges	$-27 \leq h \leq 27$, $-6 \leq k \leq 6$, $-17 \leq l \leq 17$
Reflections collected	12902
Independent reflections	2862 [R_{int} = 0.0258, R_{sigma} = 0.0203]
Data/restraints/parameters	2862/1/238
Goodness-of-fit on F^2	1.057
Final R indexes [I>=2σ (I)]	R_1 = 0.0224, wR_2 = 0.0597
Final R indexes [all data]	R_1 = 0.0227, wR_2 = 0.0600
Largest diff. peak/hole / e Å^{-3}	0.17/-0.20
Flack parameter	-0.001(7)

Table S2. Fractional atomic coordinates (×10^4) and equivalent isotropic displacement parameters (Å^2×10^3) for 22a. U_{eq} is defined as 1/3 of of the trace of the orthogonalised U_{IJ} tensor.

Atom	*x*	*y*	*z*	U(eq)
S1	7283.3(2)	5567.3(7)	3618.5(4)	14.22(11)
O1	6360.5(6)	2693(2)	5823.4(10)	18.4(3)
N1	5662.6(7)	-1074(3)	5143.8(12)	16.5(3)
N3	8127.1(7)	9217(3)	3448.8(11)	14.9(3)
N2	7011.9(7)	6805(3)	6402.8(12)	16.6(3)
O2	5490(17)	-1180(60)	3510(20)	26(4)
C11	7200.3(8)	6798(3)	5467.8(13)	13.6(3)
C1	5243.2(8)	-2943(3)	5455.7(15)	16.2(3)
C16	8024.8(8)	10400(3)	5466.0(14)	14.4(3)
C14	8468.4(8)	11054(3)	3852.8(13)	15.3(4)
C15	8430.0(8)	11646(3)	4847.8(14)	15.2(3)
C13	7744.8(8)	8001(3)	4050.3(14)	13.7(3)
C12	7658.4(8)	8470(3)	5047.7(13)	13.4(3)
C9	6535.0(8)	3132(3)	4979.0(14)	14.4(3)
C18	8907.8(9)	12432(4)	3200.2(14)	19.9(4)
C7	5816.6(9)	-433(3)	4230.3(15)	19.5(4)
C10	6970.7(8)	5097(3)	4781.1(13)	15.2(3)
C8	6305.3(8)	1620(3)	4112.7(13)	15.7(3)
C2	5006.6(8)	-4806(3)	4847.8(15)	18.3(4)
C4	4463.9(9)	-6662(3)	6209.5(17)	23.4(4)
C3	4617.5(9)	-6656(3)	5232.8(16)	21.4(4)
C17	7992.1(9)	11111(4)	6521.1(14)	18.6(4)
C6	5079.4(9)	-2927(3)	6434.9(15)	21.6(4)
C5	4696.2(10)	-4789(4)	6808.8(17)	24.8(4)
O3	5645(18)	-1460(40)	3497(7)	41(3)

Table S3. Anisotropic displacement parameters (Å^2×10^3) for thienopyridine 22a. The Anisotropic displacement factor exponent takes the form: -2π^2[h^{2}a*^{2}U$_{11}$+2hka*b*U$_{12}$+…].

**Atom**	**U$_{11}$**	U$_{22}$	U$_{33}$	U$_{23}$	U$_{13}$	U$_{12}$
S1	19.17(19)	15.43(18)	8.06(18)	-2.93(16)	0.54(16)	-1.65(13)
O1	23.3(6)	21.8(6)	10.0(6)	-1.4(5)	1.3(5)	-4.2(5)
N1	19.7(7)	16.5(6)	13.3(7)	-1.3(6)	-1.9(6)	-2.9(5)
N3	17.8(6)	15.4(6)	11.6(8)	-0.7(5)	-0.6(6)	1.0(5)
N2	21.2(7)	18.5(7)	9.9(7)	-3.3(6)	2.0(6)	-1.2(6)
O2	17(9)	41(6)	21(6)	8(7)	-5(5)	-17(6)
C11	16.5(7)	14.0(7)	10.2(8)	-0.8(6)	-2.0(7)	3.0(6)
C1	16.5(7)	14.8(7)	17.4(9)	2.4(7)	-1.4(7)	1.4(6)
C16	16.8(8)	15.2(7)	11.1(8)	-2.0(6)	-1.8(7)	2.5(6)
C14	17.5(8)	16.0(7)	12.5(9)	0.0(6)	-2.0(7)	2.5(6)
C15	17.6(7)	15.9(8)	12.3(8)	-1.9(6)	-2.0(7)	0.5(6)
C13	16.9(8)	14.5(7)	9.7(8)	-2.5(6)	-2.4(6)	2.5(6)
C12	16.6(8)	14.0(8)	9.6(8)	-1.3(7)	-0.3(6)	2.5(6)
C9	17.0(7)	15.6(7)	10.5(8)	-1.6(6)	-1.7(7)	2.7(6)
C18	22.7(8)	22.6(9)	14.3(8)	0.3(7)	0.9(7)	-4.1(7)
C7	23.5(9)	20.9(9)	14.1(9)	-1.2(7)	-0.4(8)	-5.4(7)
C10	18.2(7)	17.6(8)	9.7(8)	-1.6(7)	-0.6(7)	1.5(6)
C8	18.0(8)	19.1(8)	10.2(8)	-2.9(6)	-0.9(6)	-1.2(6)
C2	18.8(8)	17.5(7)	18.7(9)	-0.5(7)	-1.6(7)	2.3(6)
C4	23.2(8)	18.9(9)	28.1(11)	6.4(8)	2.3(8)	-0.6(6)
C3	21.7(9)	17.1(8)	25.6(10)	1.0(7)	-2.3(8)	-0.2(6)
C17	23.4(8)	21.6(8)	11.0(9)	-4.2(7)	0.6(7)	-4.3(7)
C6	27.4(9)	19.5(8)	17.9(10)	0.0(7)	0.9(8)	-0.3(7)
C5	29.0(9)	25.3(9)	20.1(11)	5.7(8)	4.4(8)	0.8(8)
O3	52(8)	58(4)	12.9(17)	-9(2)	0(3)	-39(5)

Table S4. Bond lengths for thienopyridine 22a.

Atom	Atom	Length/Å	Atom	Atom	Length/Å
S1	C13	1.7378(17)	C16	C12	1.418(2)
S1	C10	1.7551(18)	C16	C17	1.505(3)
O1	C9	1.245(2)	C14	C15	1.410(3)
N1	C1	1.410(2)	C14	C18	1.496(2)
N1	C7	1.346(3)	C13	C12	1.410(2)
N3	C14	1.344(2)	C9	C10	1.431(2)
N3	C13	1.335(2)	C9	C8	1.523(2)
N2	C11	1.351(2)	C7	C8	1.525(2)
O2	C7	1.28(3)	C7	O3	1.207(10)
C11	C12	1.448(2)	C2	C3	1.397(3)
C11	C10	1.401(2)	C4	C3	1.386(3)
C1	C2	1.395(3)	C4	C5	1.389(3)
C1	C6	1.395(3)	C6	C5	1.389(3)
C16	C15	1.387(3)			

Table S5. Bond angles for thienopyridine 22a.

Atom	Atom	Atom	Angle/°	Atom	Atom	Atom	Angle/°
C13	S1	C10	90.70(9)	C13	C12	C11	111.71(15)
C7	N1	C1	128.43(16)	C13	C12	C16	116.90(16)
C13	N3	C14	115.60(16)	O1	C9	C10	120.84(17)
N2	C11	C12	125.67(16)	O1	C9	C8	122.42(16)
N2	C11	C10	122.75(16)	C10	C9	C8	116.73(16)
C10	C11	C12	111.57(16)	N1	C7	C8	116.79(17)
C2	C1	N1	123.55(18)	O2	C7	N1	120.9(16)
C6	C1	N1	116.84(17)	O2	C7	C8	121.1(15)
C6	C1	C2	119.58(17)	O3	C7	N1	126.3(6)
C15	C16	C12	116.47(17)	O3	C7	C8	116.6(7)
C15	C16	C17	120.15(16)	C11	C10	S1	112.93(14)
C12	C16	C17	123.37(17)	C11	C10	C9	125.14(17)
N3	C14	C15	122.30(16)	C9	C10	S1	121.92(14)
N3	C14	C18	116.91(16)	C9	C8	C7	121.30(16)
C15	C14	C18	120.78(16)	C1	C2	C3	119.49(19)
C16	C15	C14	121.81(16)	C3	C4	C5	119.38(18)
N3	C13	S1	120.06(14)	C4	C3	C2	120.84(18)
N3	C13	C12	126.89(16)	C5	C6	C1	120.27(19)
C12	C13	S1	113.05(13)	C6	C5	C4	120.4(2)
C16	C12	C11	131.38(17)				

Table S6. Torsion angles for thienopyridine 22a.

A	B	C	D	Angle/°	A	B	C	D	Angle/°
S1	C13	C12	C11	-0.22(18)	C13	S1	C10	C11	1.69(14)
S1	C13	C12	C16	178.94(12)	C13	S1	C10	C9	-177.41(15)
O1	C9	C10	S1	173.72(13)	C13	N3	C14	C15	0.5(2)
O1	C9	C10	C11	-5.3(3)	C13	N3	C14	C18	179.24(15)
O1	C9	C8	C7	3.5(3)	C12	C11	C10	S1	-2.12(19)
N1	C1	C2	C3	176.81(17)	C12	C11	C10	C9	176.96(15)
N1	C1	C6	C5	-176.43(17)	C12	C16	C15	C14	1.0(2)
N1	C7	C8	C9	-8.8(3)	C18	C14	C15	C16	179.78(16)
N3	C14	C15	C16	-1.6(3)	C7	N1	C1	C2	13.5(3)
N3	C13	C12	C11	179.33(15)	C7	N1	C1	C6	-168.51(19)
N3	C13	C12	C16	-1.5(3)	C10	S1	C13	N3	179.60(14)
N2	C11	C12	C16	1.8(3)	C10	S1	C13	C12	-0.81(13)
N2	C11	C12	C13	-179.23(16)	C10	C11	C12	C16	-177.51(17)
N2	C11	C10	S1	178.58(13)	C10	C11	C12	C13	1.5(2)
N2	C11	C10	C9	-2.3(3)	C10	C9	C8	C7	-176.97(15)
O2	C7	C8	C9	159(2)	C8	C9	C10	S1	-5.8(2)
C1	N1	C7	O2	14.4(18)	C8	C9	C10	C11	175.20(16)
C1	N1	C7	C8	-177.93(16)	C2	C1	C6	C5	1.7(3)
C1	N1	C7	O3	-4(3)	C3	C4	C5	C6	-0.1(3)
C1	C2	C3	C4	0.0(3)	C17	C16	C15	C14	-179.21(16)
C1	C6	C5	C4	-1.1(3)	C17	C16	C12	C11	-0.4(3)
C14	N3	C13	S1	-179.45(12)	C17	C16	C12	C13	-179.38(16)
C14	N3	C13	C12	1.0(2)	C6	C1	C2	C3	-1.1(3)
C15	C16	C12	C11	179.36(17)	C5	C4	C3	C2	0.6(3)
C15	C16	C12	C13	0.4(2)	O3	C7	C8	C9	177(2)

Table S7. Hydrogen atom coordinates (Å×10^4) and isotropic displacement parameters (Å^2×10^3) for thienopyridine 22a.

Atom	*x*	*y*	*z*	U(eq)
H1	5847.52	-215.16	5606.61	20
H15	8688.7	12933.08	5101.87	18
H18A	9336.09	11909.25	3340.36	30
H18B	8866.77	14239.47	3310.45	30
H18C	8808.23	12048.59	2522.22	30
H8A	6675.67	833.27	3812.05	19
H8B	6139.11	2834.04	3634.73	19
H2	5109.19	-4816.94	4177.35	22

Table S7. Hydrogen atom coordinates (Å×10^4) and isotropic displacement parameters (Å^2×10^3) for thienopyridine 22a.

Atom	*x*	*y*	*z*	U(eq)
H4	4202.18	-7934.96	6466.6	28
H3	4456.17	-7925.43	4819.82	26
H17A	8297.56	12431.56	6656.07	28
H17B	8084.34	9639.3	6921.83	28
H17C	7573.06	11726.14	6672.74	28
H6	5230.65	-1636.71	6847.76	26
H5	4592.29	-4781.15	7478.83	30
H2A	6693(12)	5700(50)	6550(20)	24(6)
H2B	7097(14)	8230(60)	6770(30)	39(8)

Table S8. Atomic occupancy for thienopyridine 22a.

Atom	*Occupancy*	Atom	*Occupancy*	Atom	*Occupancy*
O2	0.24(11)	O3	0.76(11)		

Experimental

Single crystals of $C_{18}H_{17}N_3O_2S$ **22a** were grown from BuOH. A suitable crystal was selected and mounted on the glass stick by acrylic glue on a SuperNova, Dual, Cu at home/near, AtlasS2 diffractometer. The crystal was kept at 99.9(2) K during data collection. Using Olex2 [1], the structure was solved with the SHELXT [2] structure solution program using Intrinsic Phasing and refined with the SHELXL [3] refinement package using Least Squares minimisation.

1. Dolomanov, O.V., Bourhis, L.J., Gildea, R.J, Howard, J.A.K. & Puschmann, H. (2009), J. Appl. Cryst. 42, 339-341.
2. Sheldrick, G.M. (2015). Acta Cryst. A71, 3-8.
3. Sheldrick, G.M. (2015). Acta Cryst. C71, 3-8.

Crystal structure determination of 22a

Crystal Data for $C_{18}H_{17}N_3O_2S$ (M =339.40 g/mol): orthorhombic, space group Pca2$_1$ (no. 29), a = 21.46758(18) Å, b = 5.33292(4) Å, c = 13.78236(14) Å, V = 1577.87(2) Å^3, Z = 4, T = 99.9(2) K, μ(Cu Kα) = 1.959 mm^{-1}, *Dcalc* = 1.429 g/cm^3, 12902 reflections measured (8.238° ≤ 2Θ ≤ 152.524°), 2862 unique (R_{int} = 0.0258, R_{sigma} = 0.0203) which were used in all calculations. The final R_1 was 0.0224 (I > 2σ(I)) and wR_2 was 0.0600 (all data).

Experimental FT-IR spectrum (ATR mode) of thienopyridine 22a

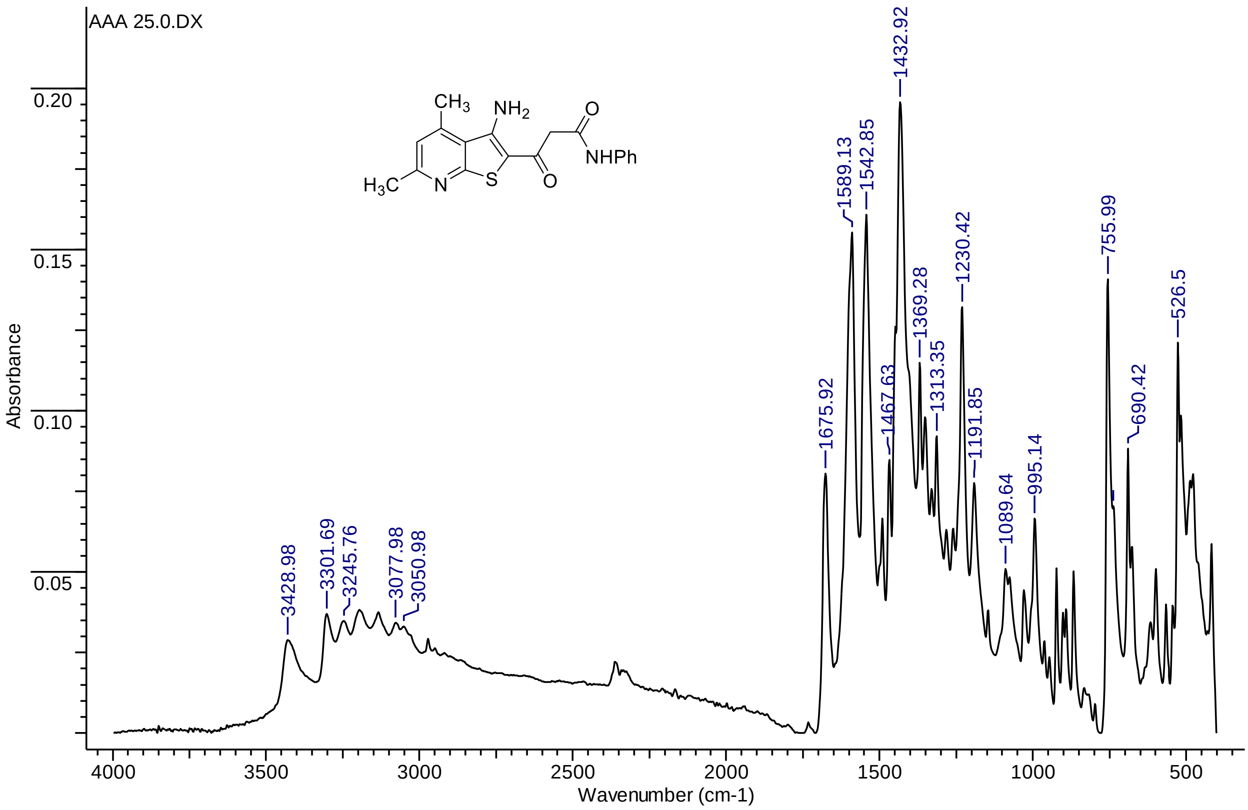

IR spectrum of the most stable conformer K1 of thienopyridine 22a calculated at the B3LYP-D3BJ/6-311+G(2d,p) level of theory

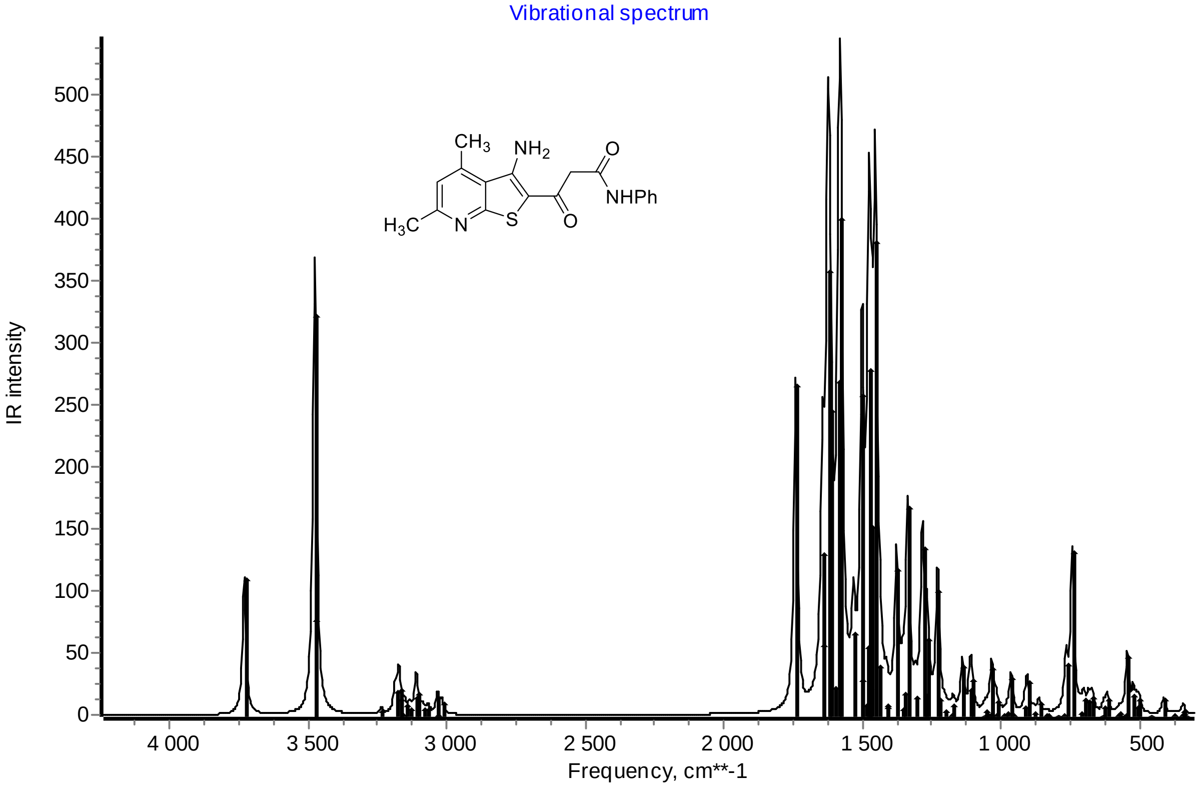

FT-IR spectrum (ATR mode) of thienopyridine 22b

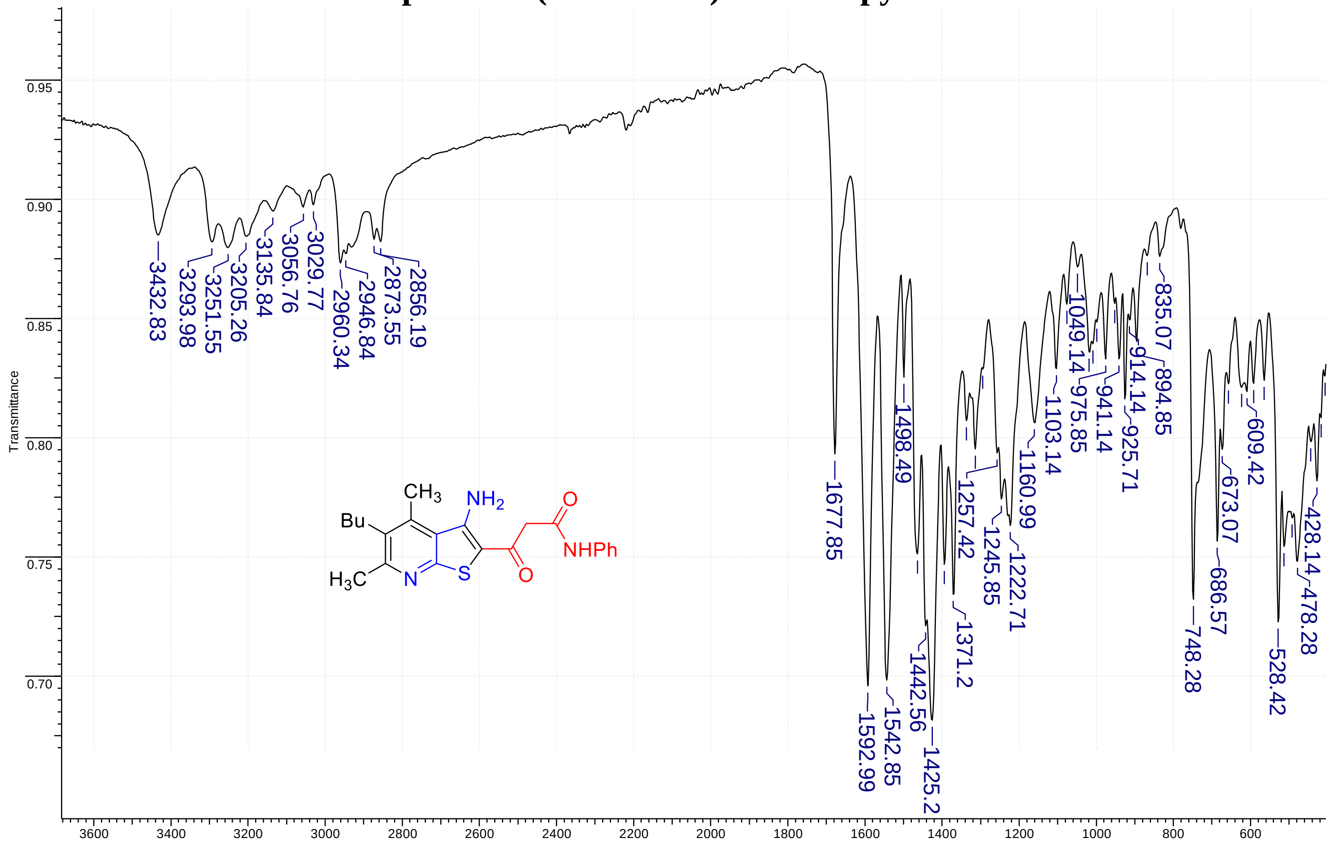

^{1}H NMR spectrum (400 MHz, DMSO-d$_6$) of thienopyridine 22b

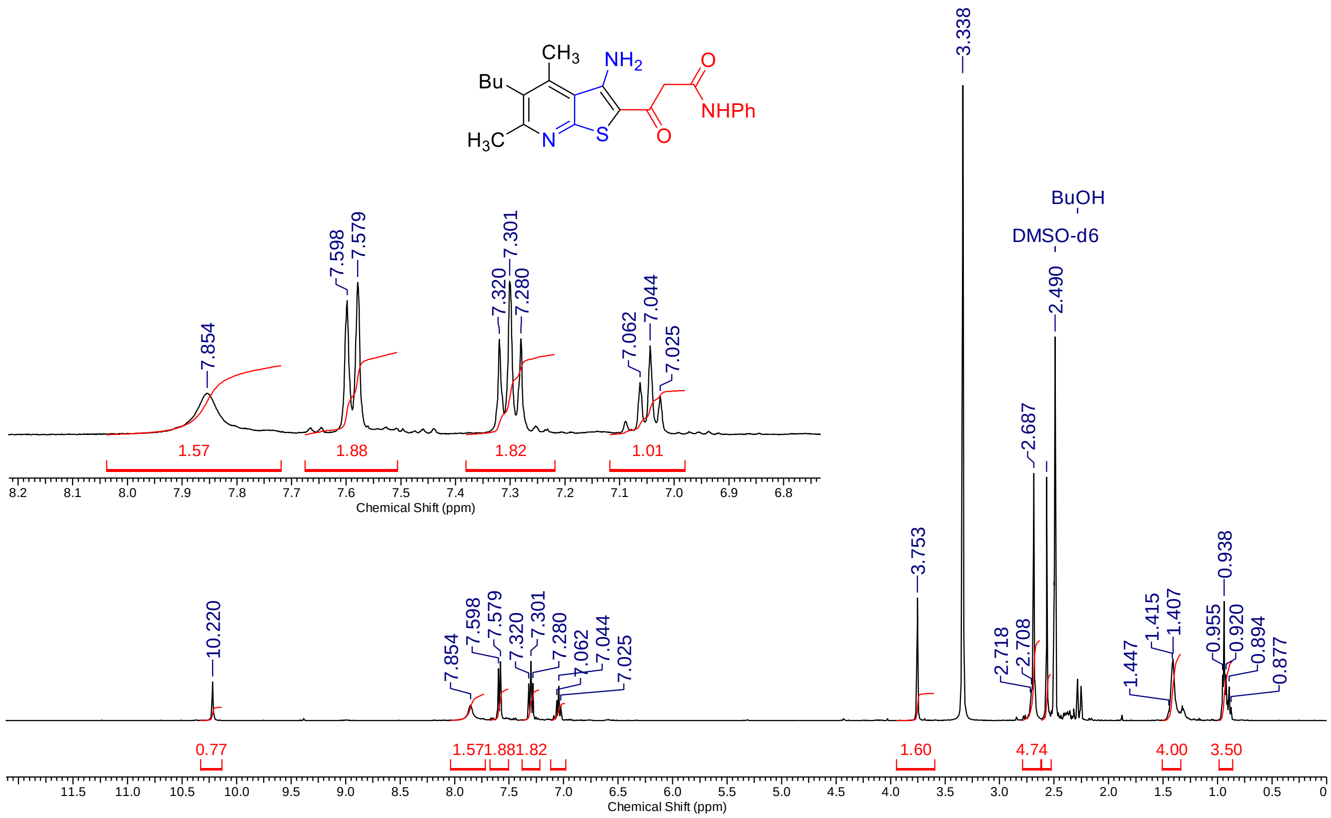

^{13}C DEPTQ NMR spectrum (101 MHz, DMSO-d$_6$) of thienopyridine 22b

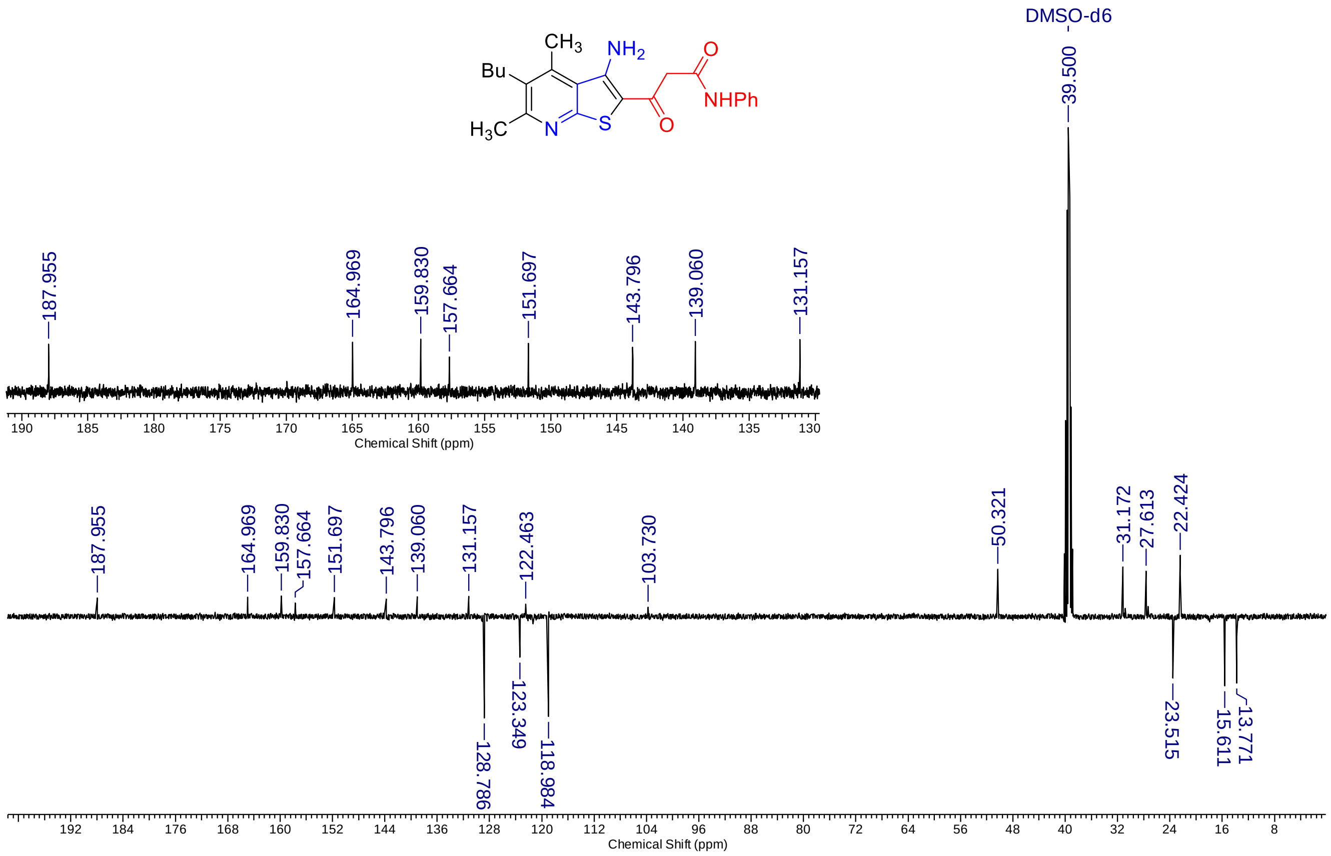

FT-IR spectrum (ATR mode) of thienopyridine 22c

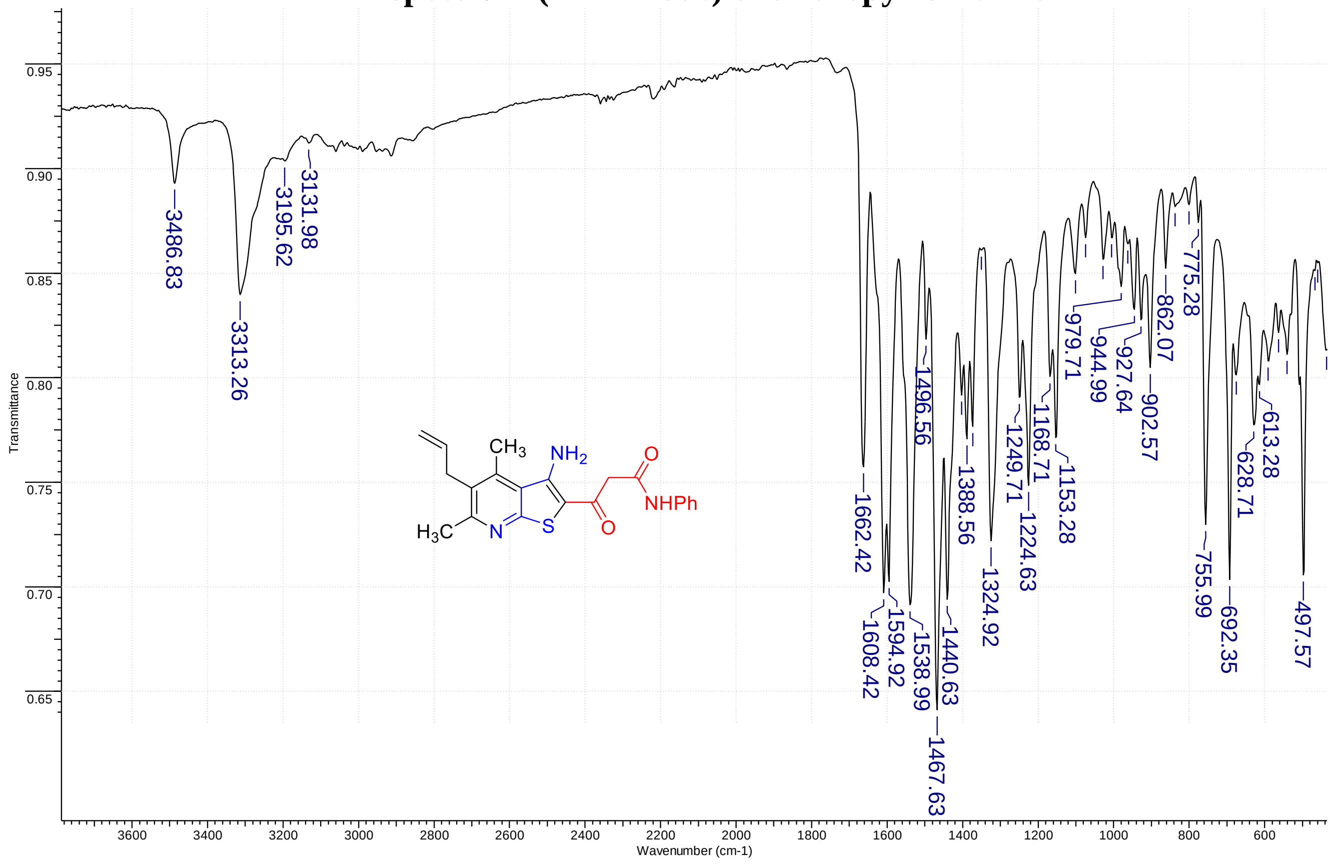

^{1}H NMR spectrum (400 MHz, DMSO-d$_6$) of thienopyridine 22c

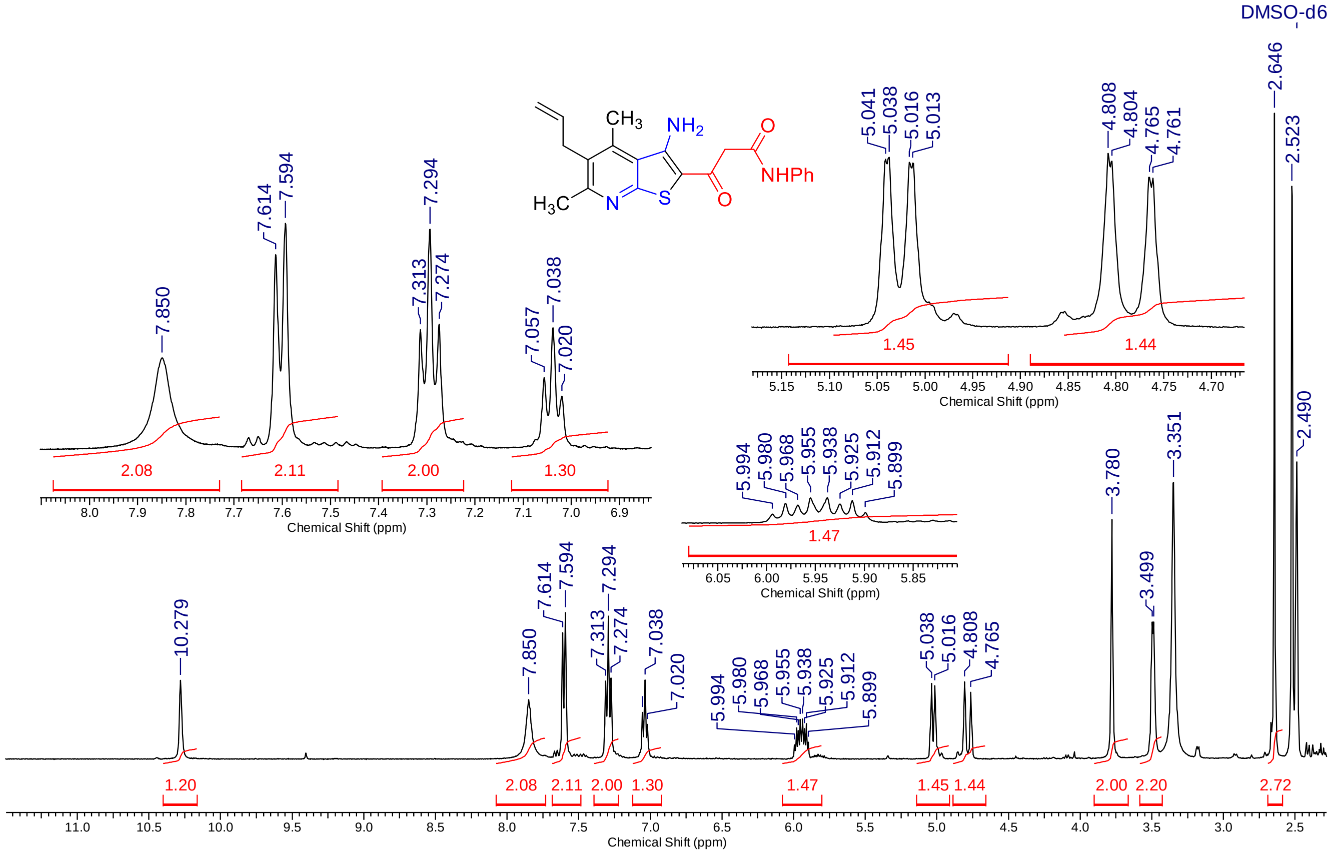

^{13}C NMR spectrum (101 MHz, DMSO-d$_6$) of thienopyridine 22c

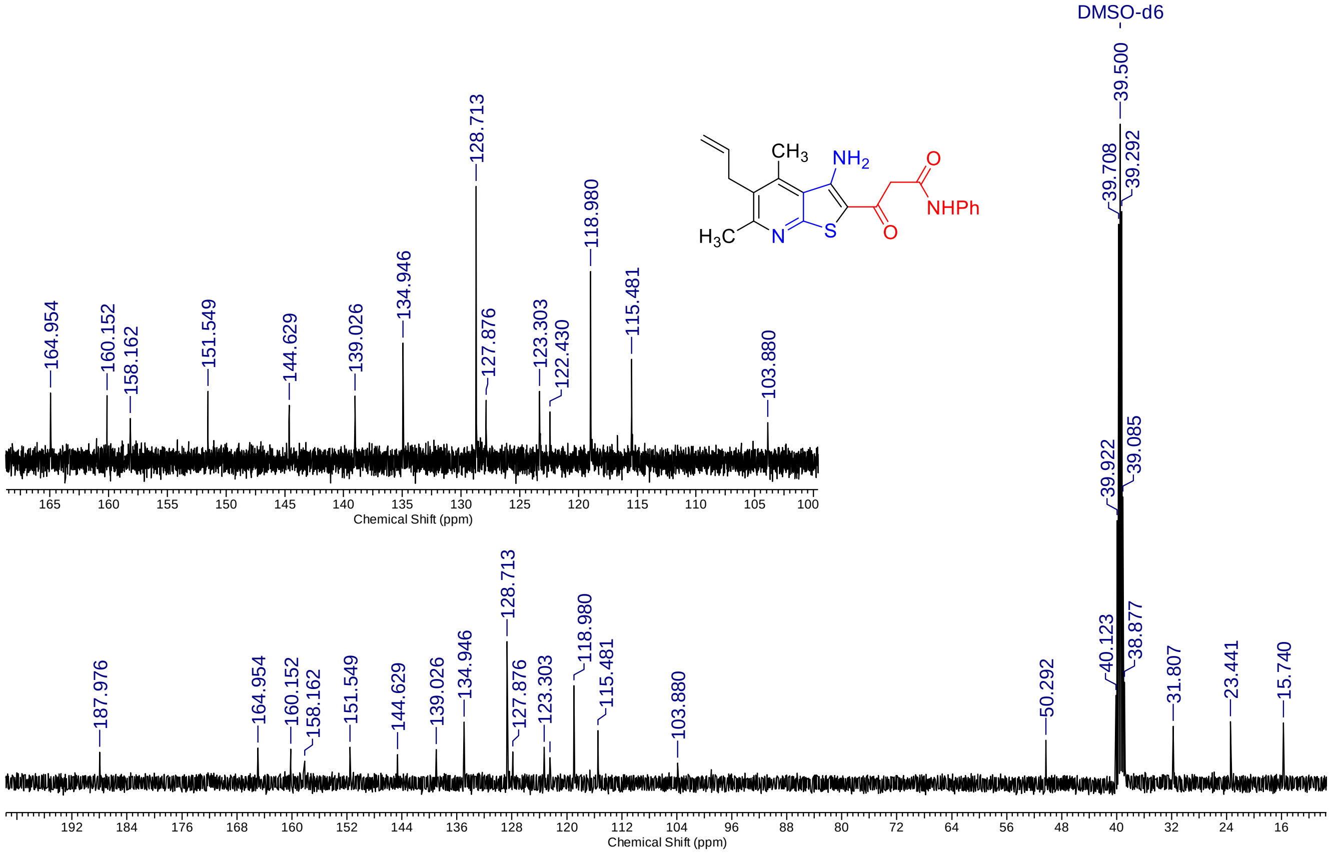

^{1}H–^{13}C HSQC NMR spectrum (400/101 MHz, DMSO-d_6) of thienopyridine 22c

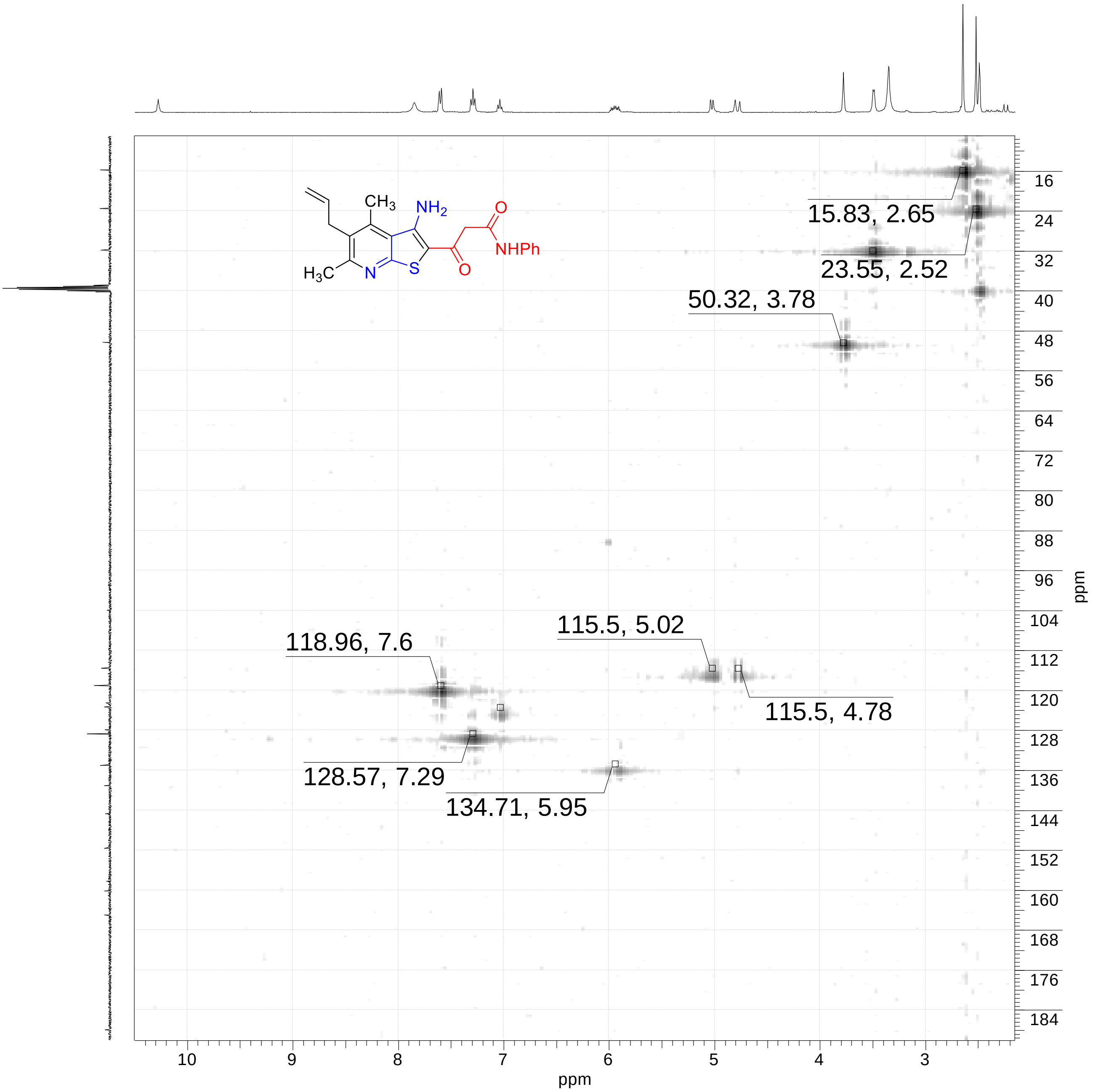

^{1}H–^{13}C HSQC NMR spectrum (400/101 MHz, DMSO-d$_6$) of thienopyridine 22c (*fragment*)

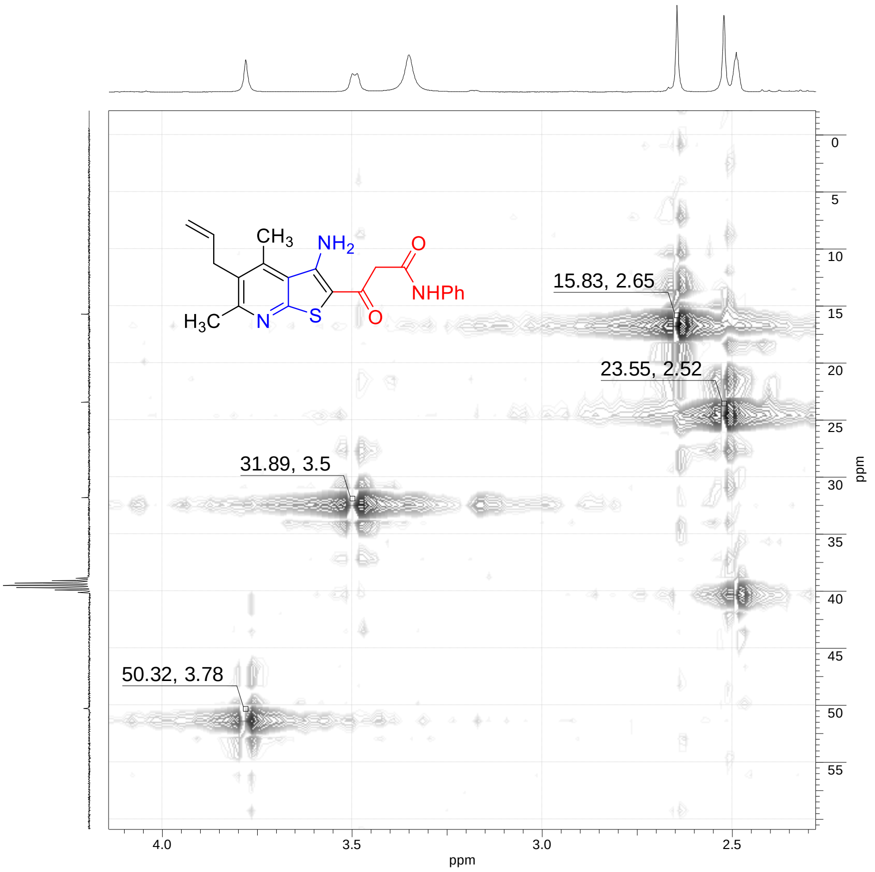

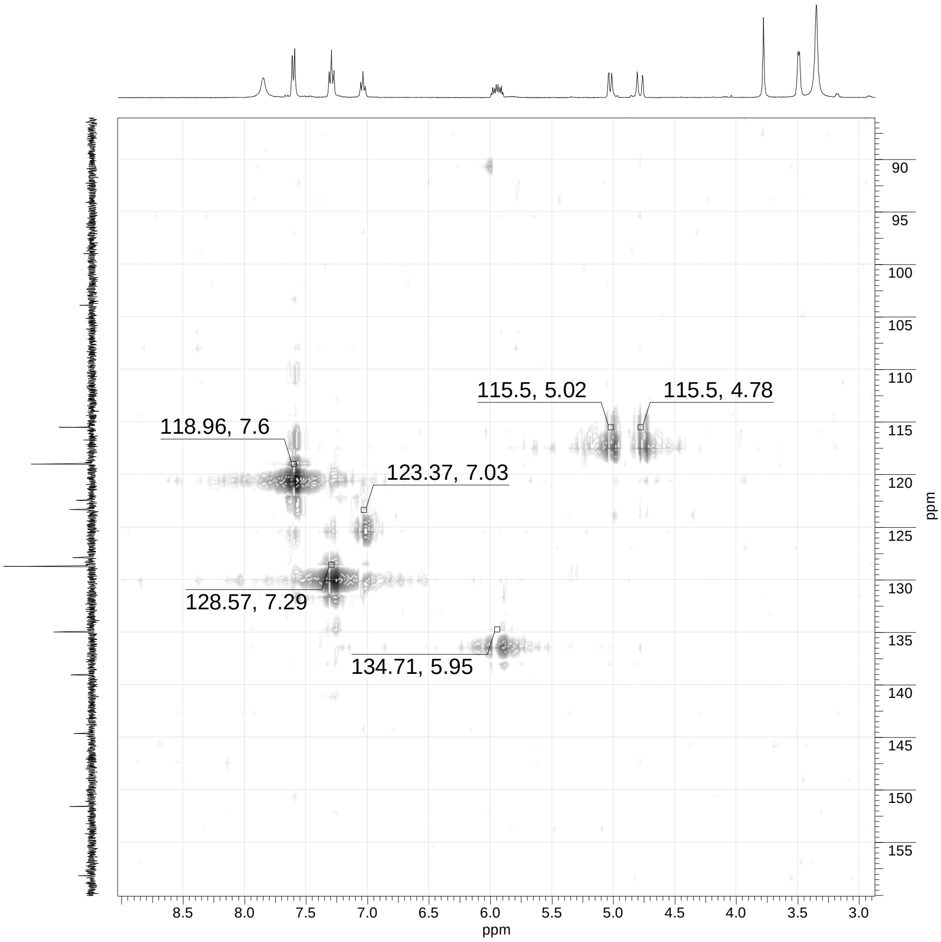

^{1}H–^{13}C HMBC NMR spectrum (400/101 MHz, DMSO-d$_6$) of thienopyridine 22c

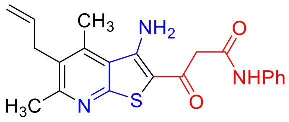

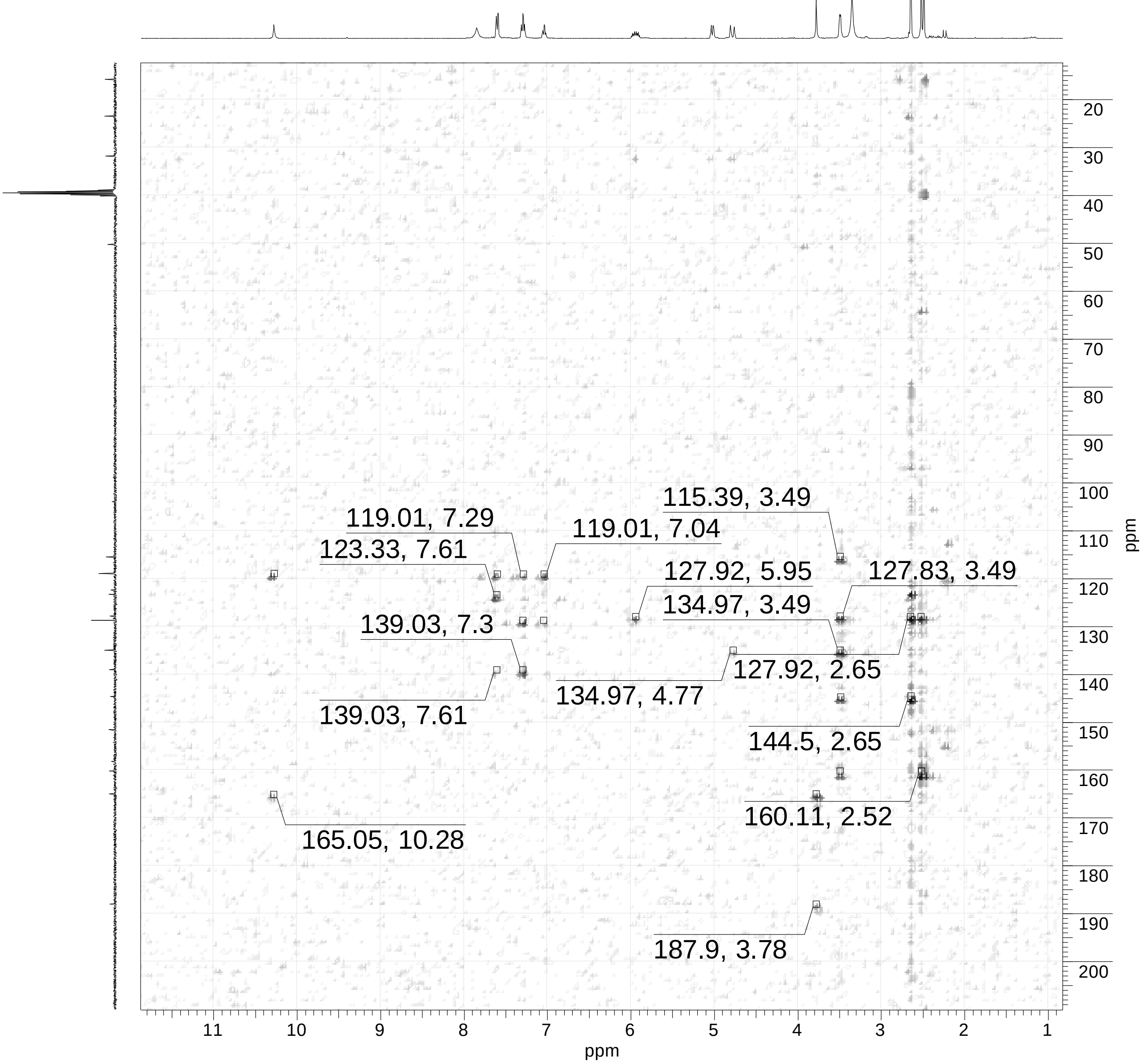

^{1}H–^{13}C HMBC NMR spectrum (400/101 MHz, DMSO-d_6) of thienopyridine 22c

fragments

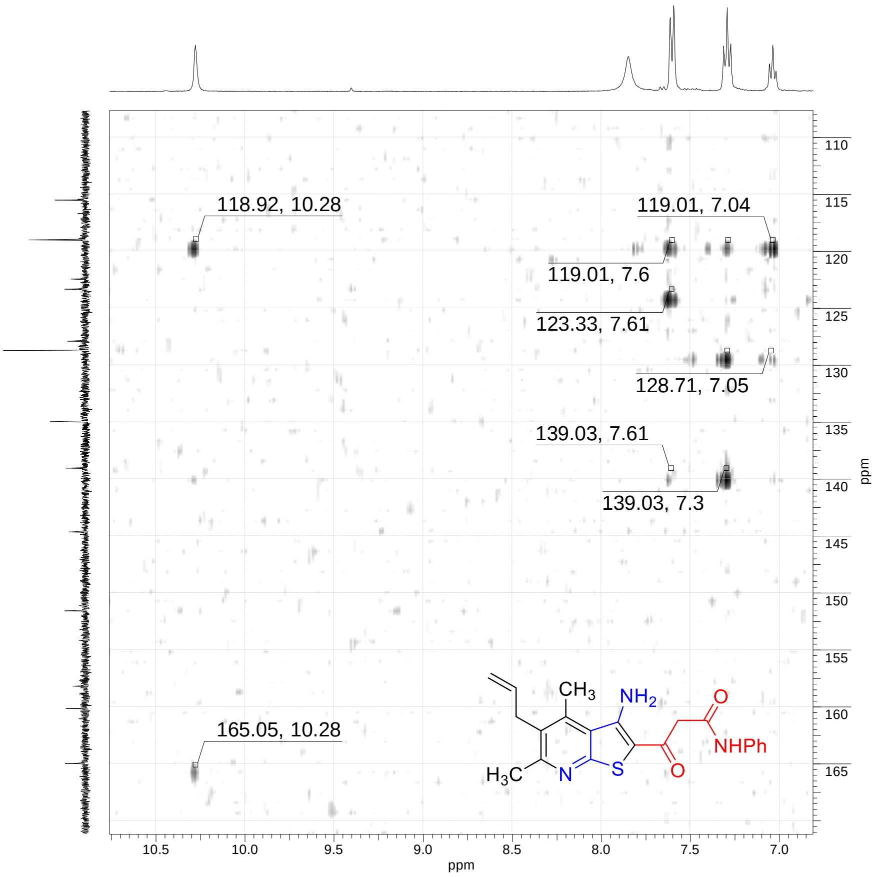

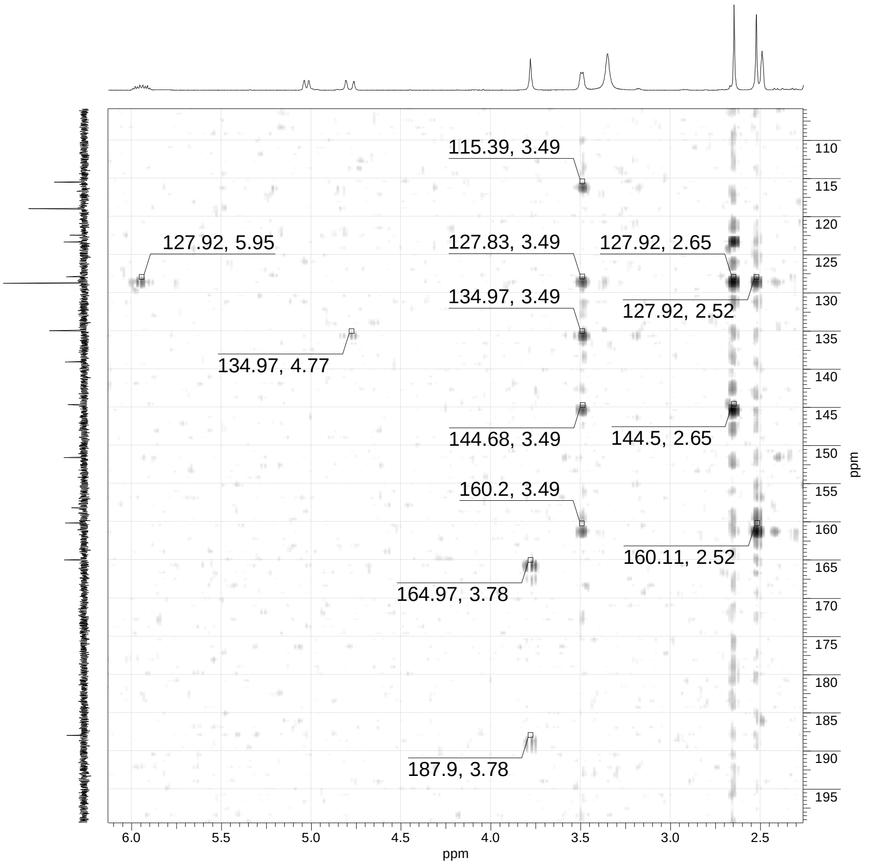

Table S9. The observed correlations in the ^{1}H-^{13}C HSQC and ^{1}H-^{13}C HMBC 2D NMR spectra (400/101 MHz, DMSO-d_6) of thienopyridine 22c

^{13}C chemical shifts are given in **red**, ^{1}H shifts – in **blue**

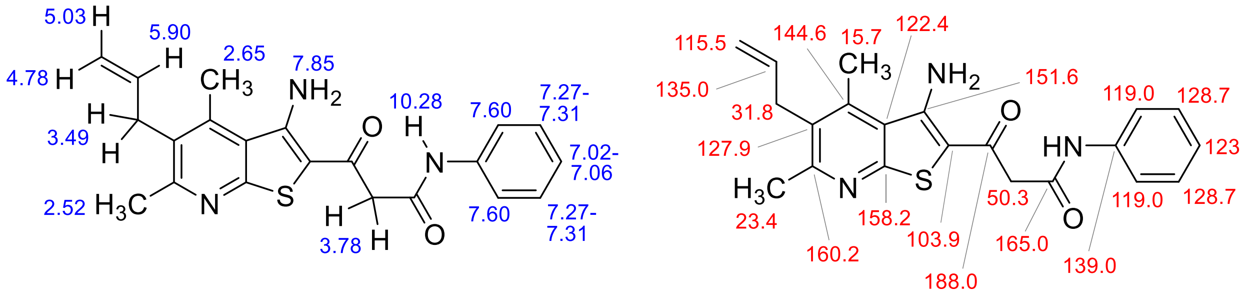

^{1}H NMR shifts, δ, ppm	Correlations in HSQC spectrum, δ, ppm	Correlations in HMBC spectrum, δ, ppm
2.52 (s, 3H, C(6)–CH_3)	23.4 (C(6)–$\underline{C}H_3$)	127.9 (C-5), 160.2 (C-6)
2.65 (s, 3H, C(4)–CH_3)	15.7 (C(4)–$\underline{C}H_3$)	122.4 (C-3a), 127.9 (C-5), 144.6 (C-4)
3.49 (br d, ^{3}J = 4.8 Hz, 2H, $C\underline{H}_2CH=CH_2$)	31.8 ($\underline{C}H_2CH=CH_2$)	115.5 (=CH_2), 127.9 (C-5), 135.0 ($CH_2\underline{C}H=CH_2$), 144.6 (C-4), 160.2 (C-6)
3.78 (s, 2H, CH_2C(O))	50.3 (C(O)CH_2)	165.0 (CONH), 188.0 (C=O)
4.78 (dd, ^{3}J = 17.5 Hz, ^{2}J = 1.4 Hz, *trans*-CH=$C\underline{H}_2$)	115.5 (=CH_2)	135.0 ($CH_2\underline{C}H=CH_2$)
5.03 (dd, ^{3}J = 9.9 Hz, ^{2}J = 1.4 Hz, *cis*-CH=$C\underline{H}_2$)	115.5 (=CH_2)	–
5.90-5.99 (m, $CH_2C\underline{H}=CH_2$)	135.0 ($CH_2\underline{C}H=CH_2$)	127.9 (C-5)
7.02-7.06 (m, 1H, C(4)H Ph)	123.3 (C-4 Ph)	119.0 (2C, C-2, C-6 Ph), 128.7 (2C, C-3, C-5 Ph)
7.27-7.31 (m, 2H, C(3)H, C(5)H Ph)	128.7 (2C, C-3, C-5 Ph)	119.0 (2C, C-2, C-6 Ph), 128.7 (2C, C-3, C-5 Ph), 139.0 (C-1 Ph)
7.60 (d, ^{3}J = 7.9 Hz, 2H, C(2)H, C(6)H Ph)	119.0 (2C, C-2, C-6 Ph)	119.0 (2C, C-2, C-6 Ph), 123.3 (C-4 Ph), 139.0 (C-1 Ph)
7.85 (br.s, 2H, NH_2)	–	–
10.28 (CONH)	–	119.0 (2C, C-2, C-6 Ph), 165.0 (CONH)

FT-IR spectrum (ATR mode) of thienopyridine 22d

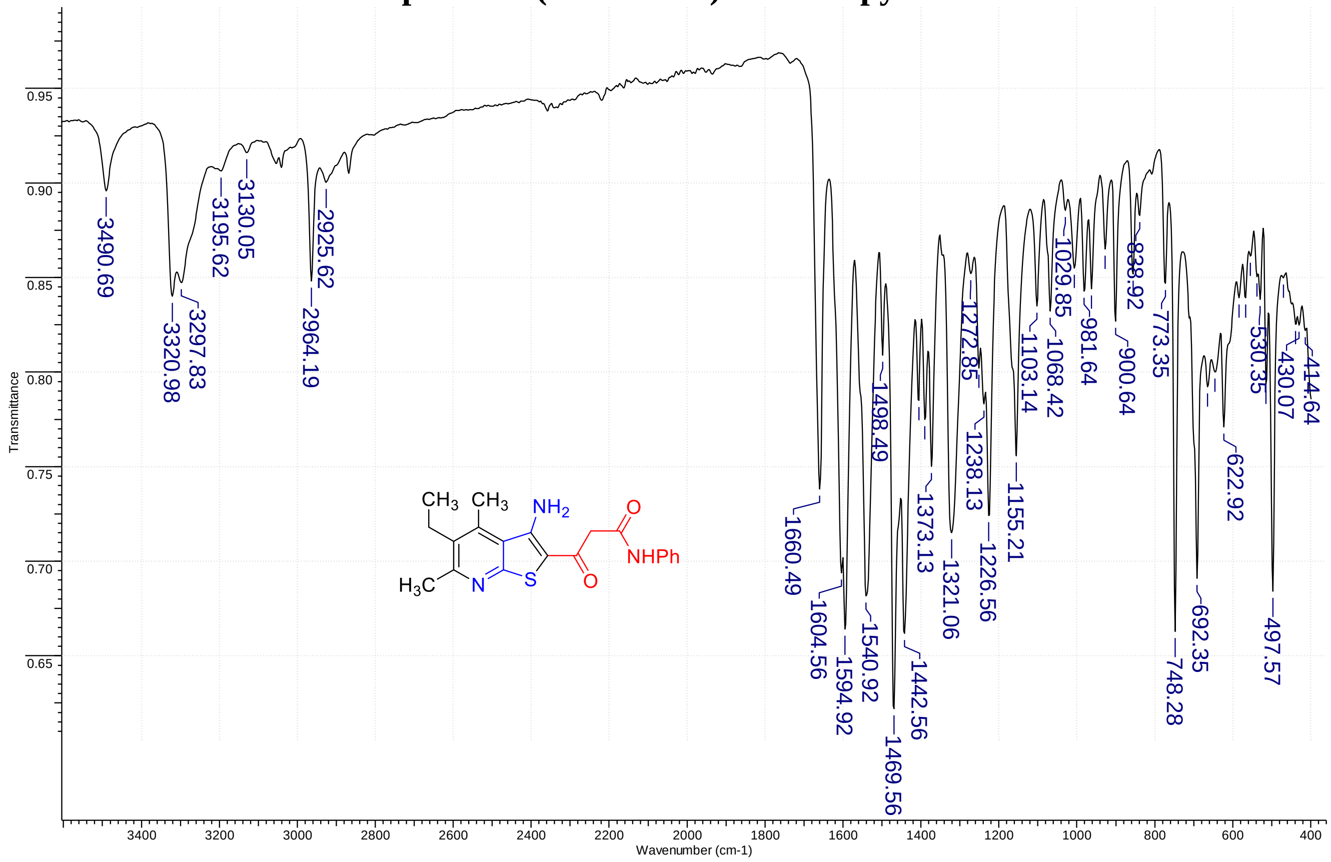

^{1}H NMR spectrum (400 MHz, DMSO-d_6) of thienopyridine 22d

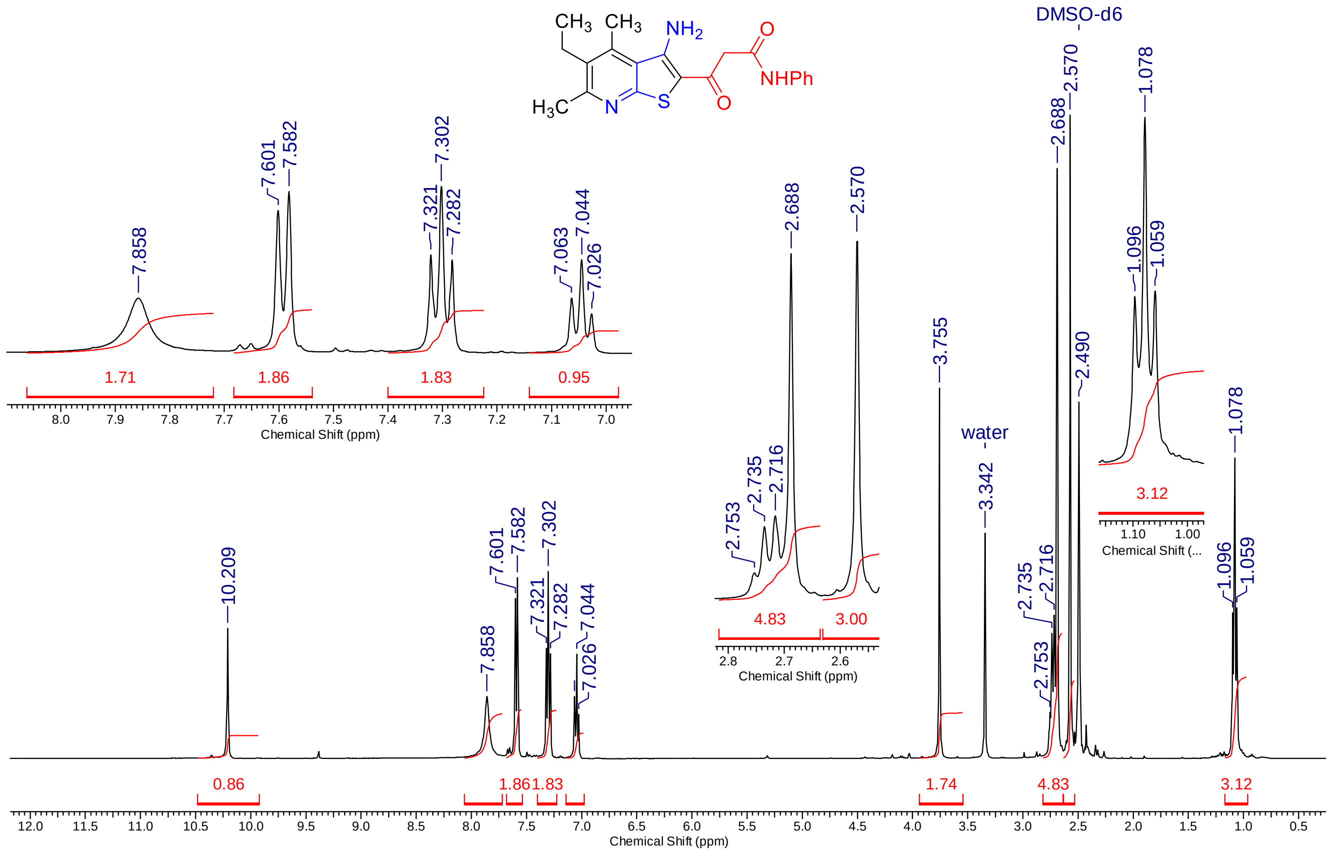

^{13}C DEPTQ NMR spectrum (101 MHz, DMSO-d6) of thienopyridine 22d

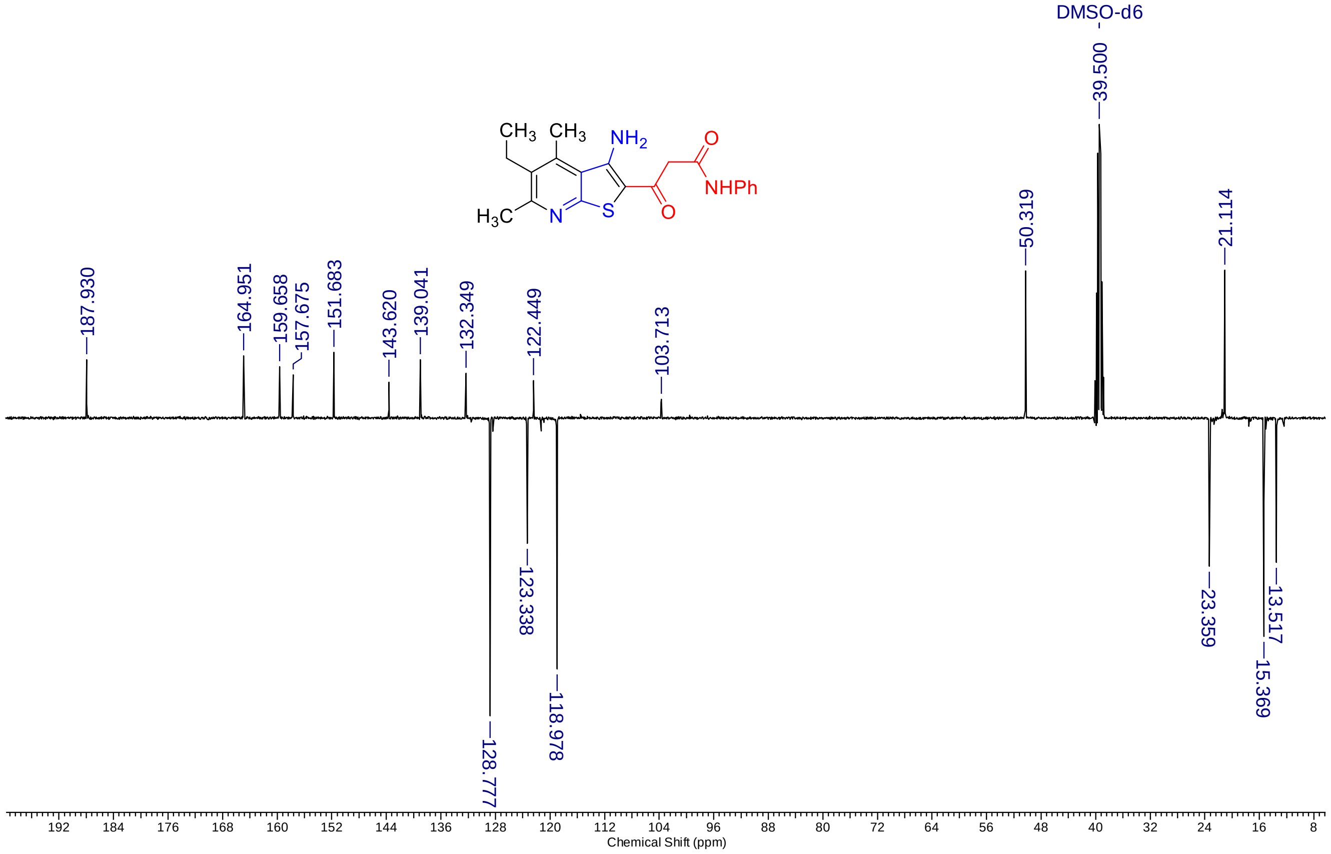

FT-IR spectrum (ATR mode) of thienopyridine 22e

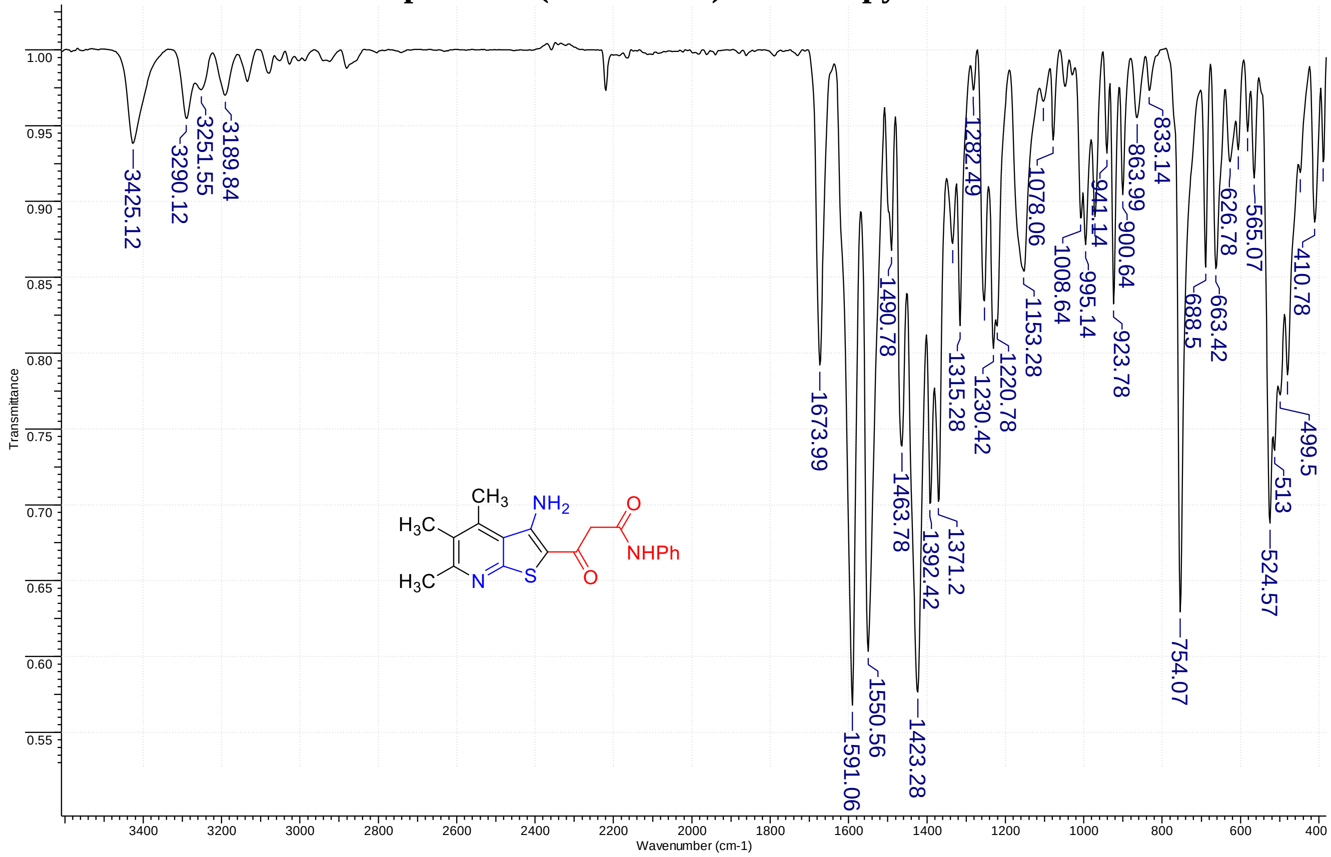

^{1}H NMR spectrum (400 MHz, DMSO-d$_6$) of thienopyridine 22e

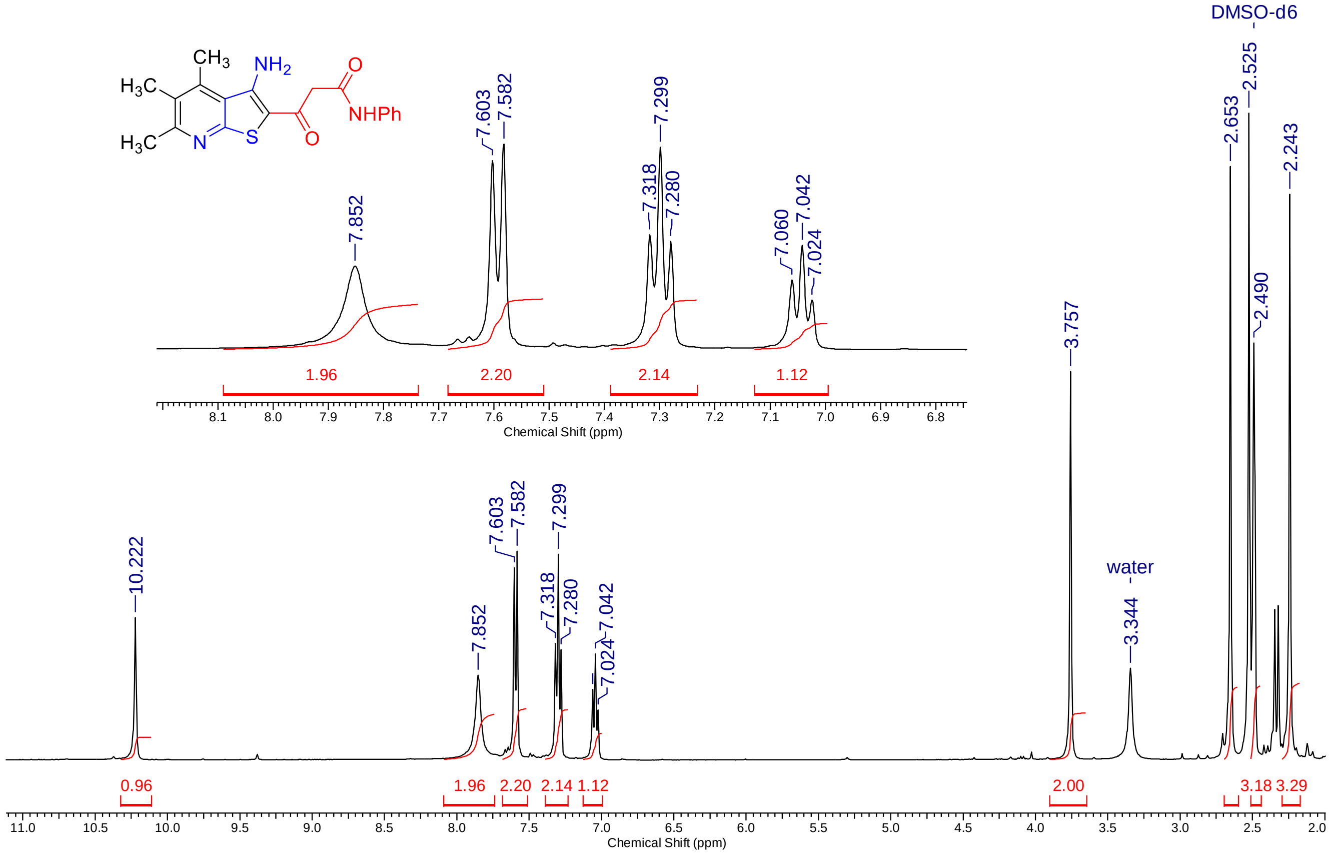

^{13}C DEPTQ NMR spectrum (101 MHz, DMSO-d$_6$) of thienopyridine 22e

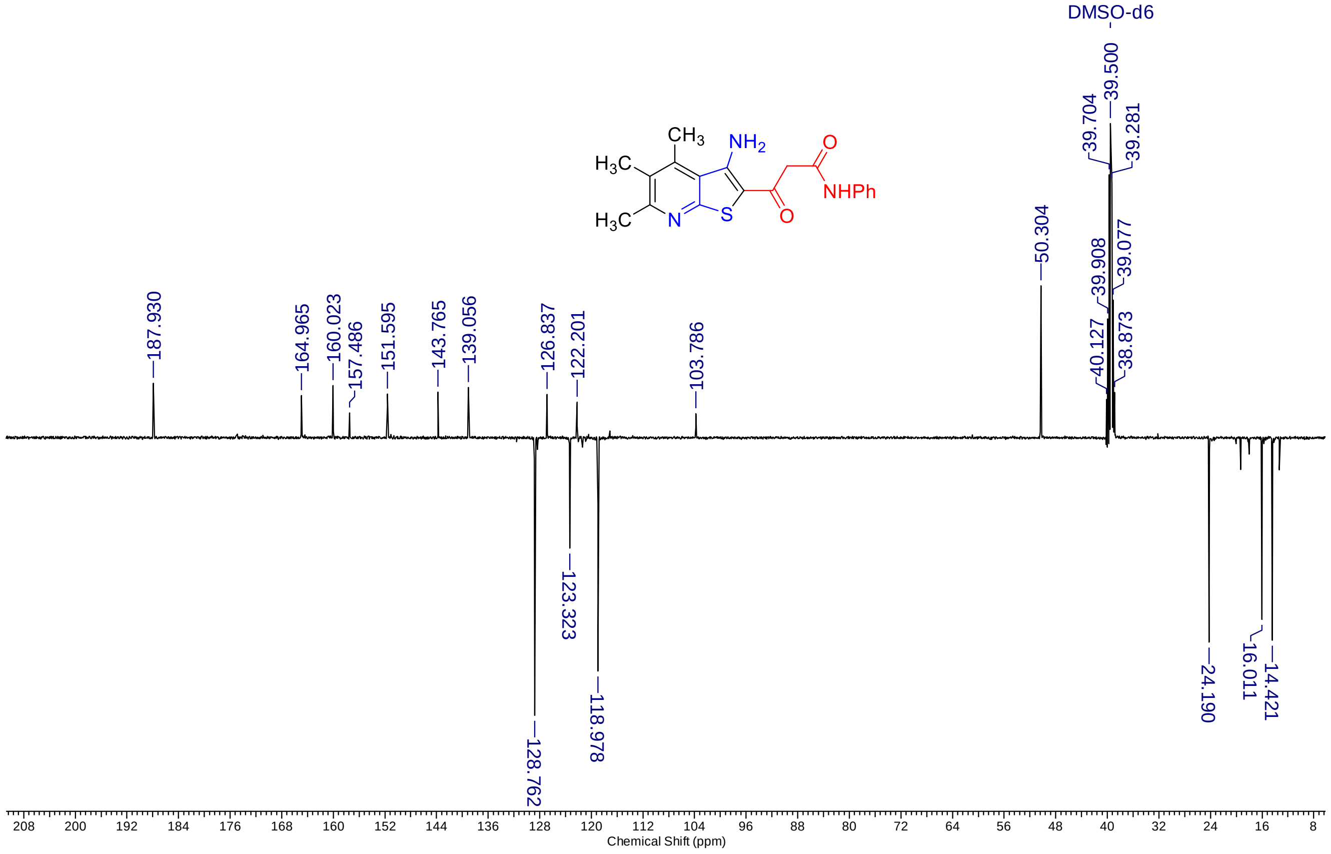

FT-IR spectrum (ATR mode) of thienopyridine 22f

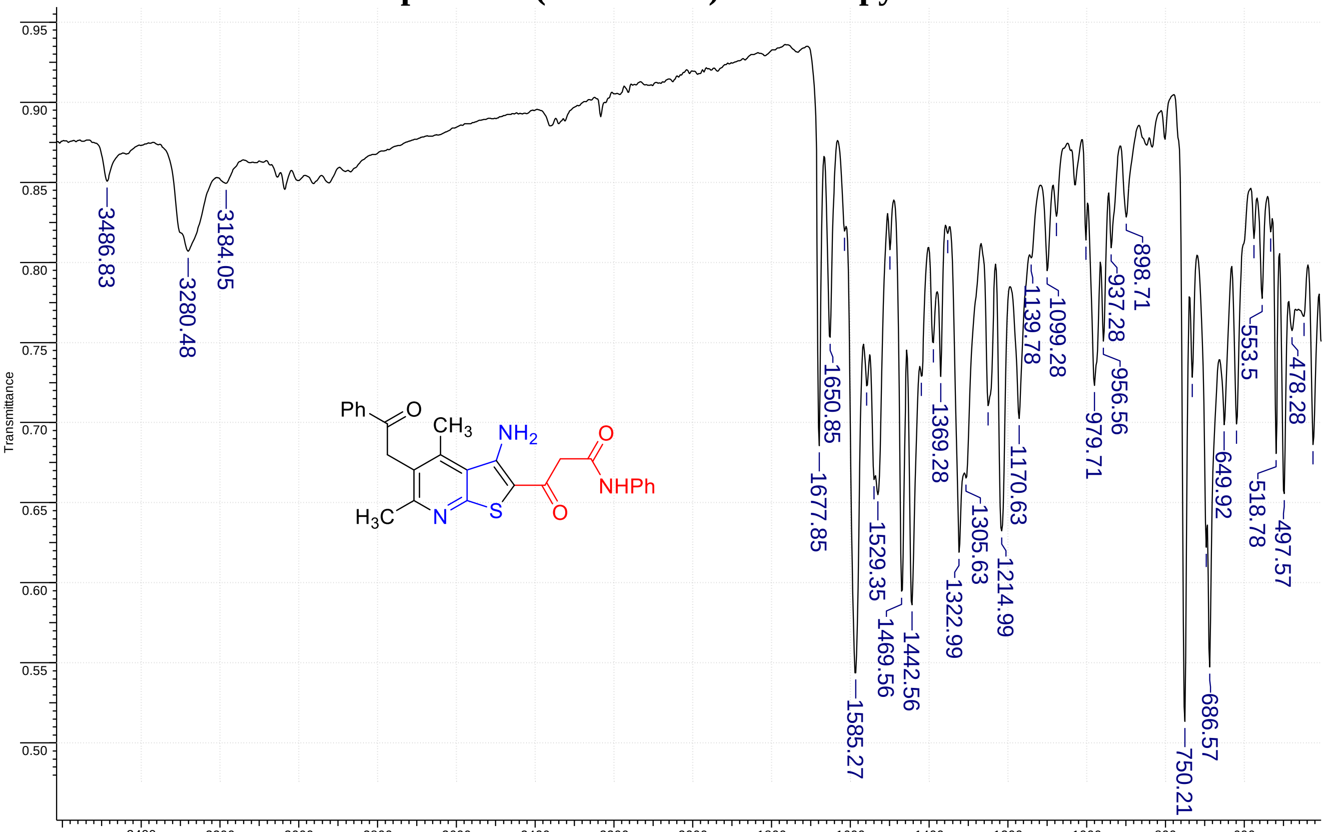

^{1}H NMR spectrum (400 MHz, DMSO-d$_6$) of thienopyridine 22f

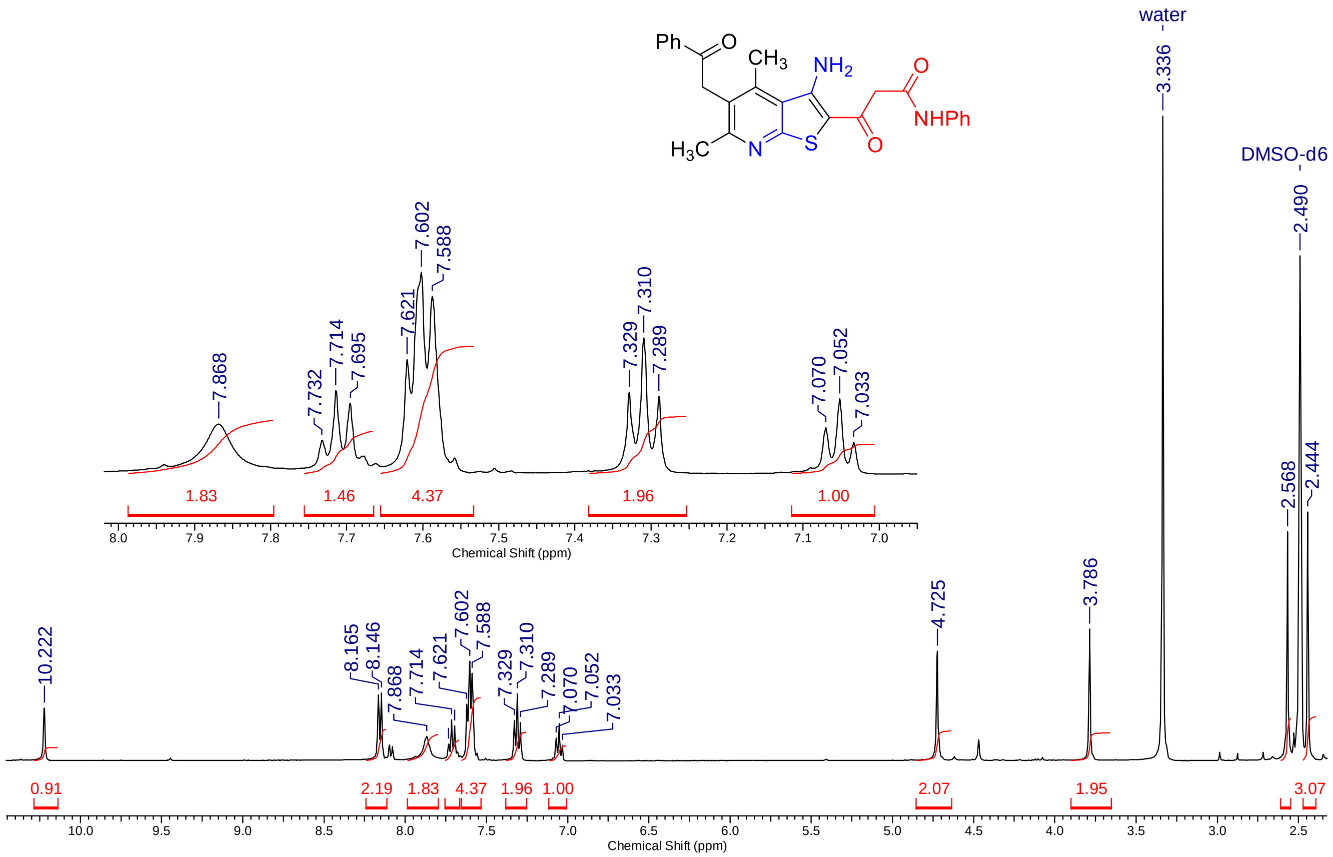

^{13}C DEPTQ NMR spectrum (101 MHz, DMSO-d_6) of thienopyridine 22f

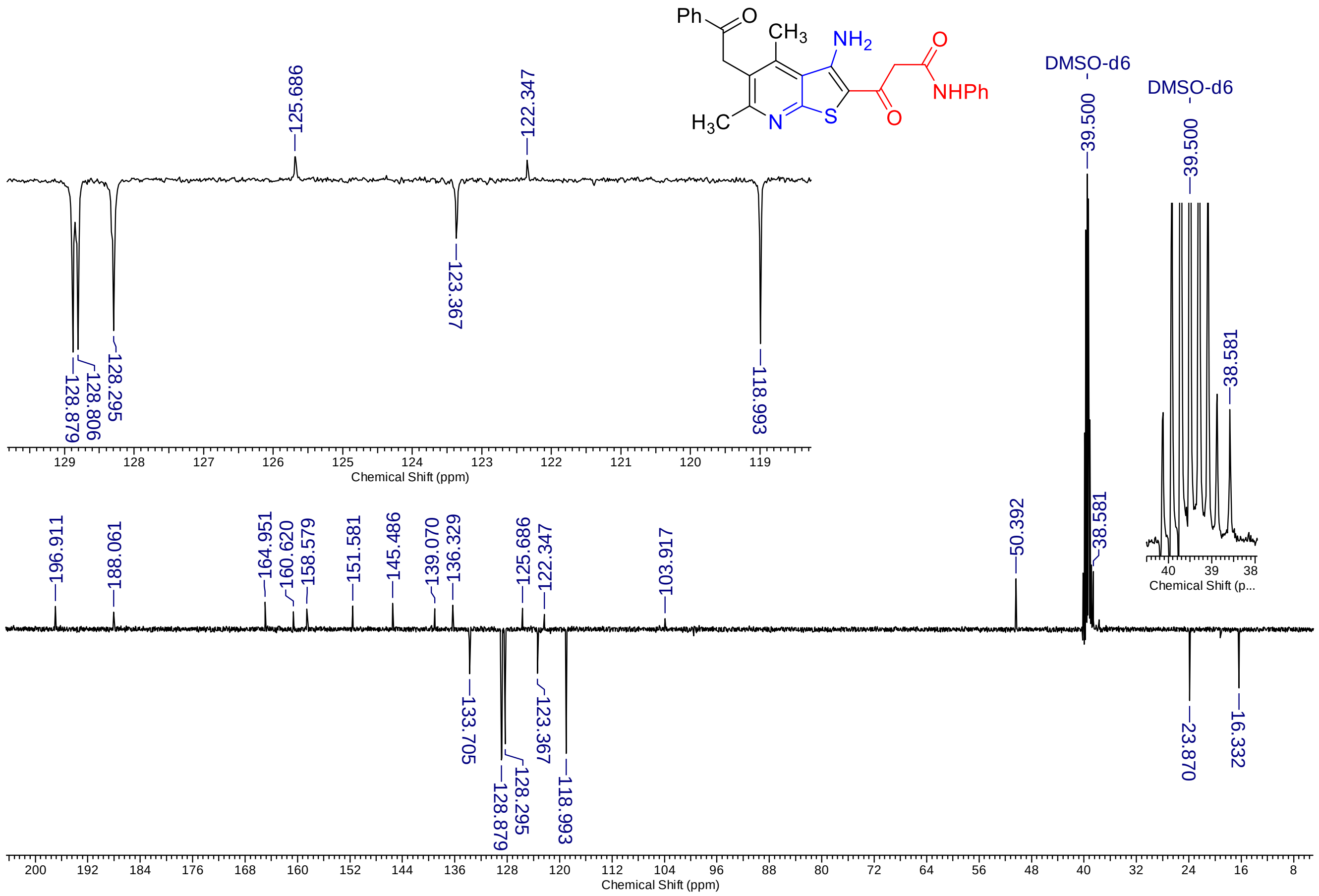

^1H–^{13}C HMBC NMR spectrum (400/101 MHz, DMSO-d_6) of thienopyridine 22f

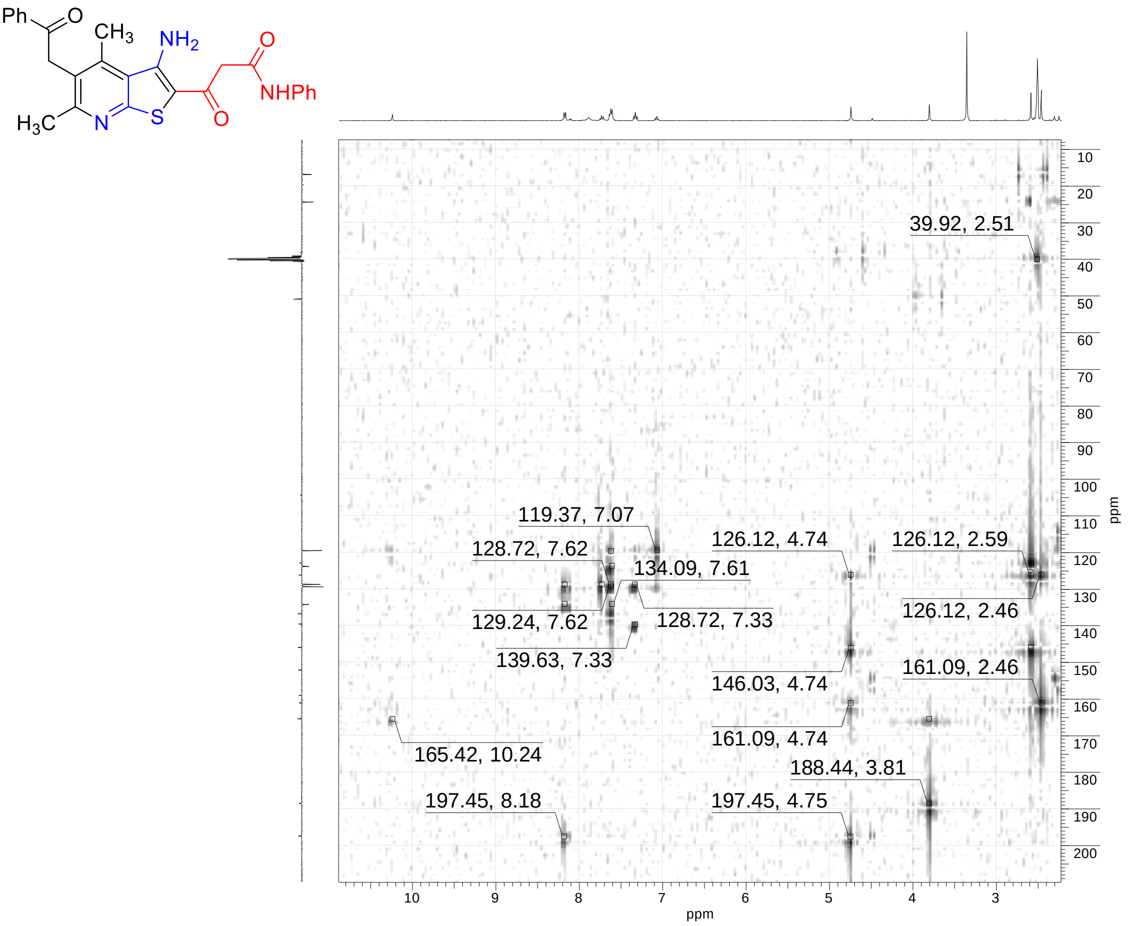

^{1}H–^{13}C HMBC NMR spectrum (400/101 MHz, DMSO-d_6) of thienopyridine 22f

fragments

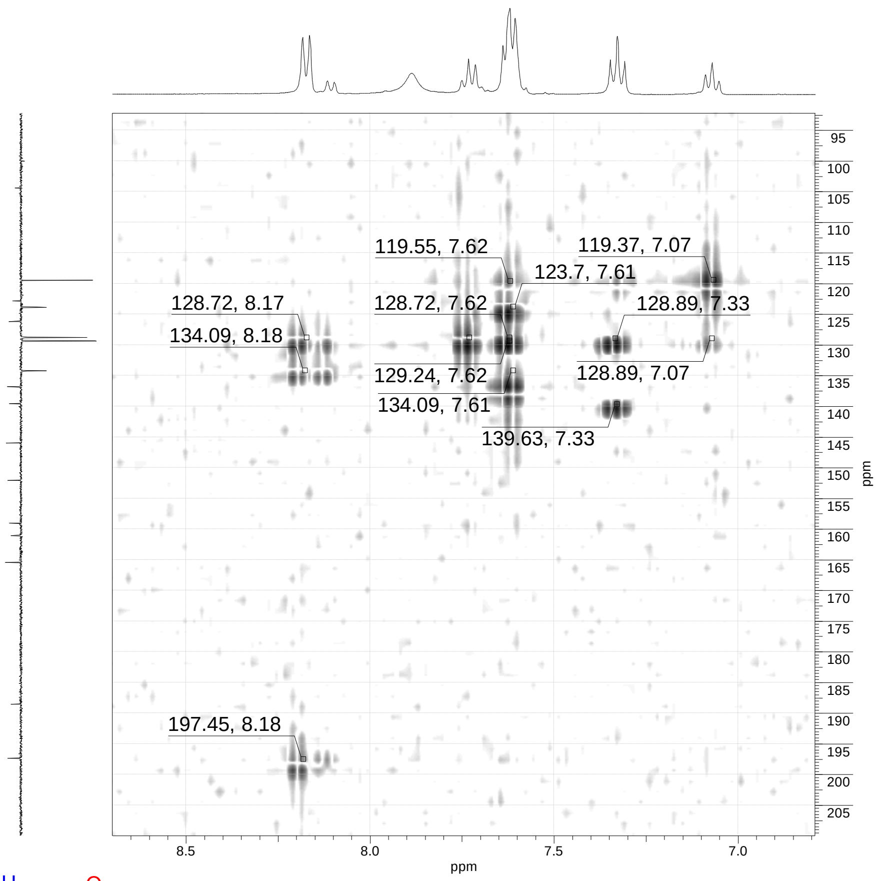

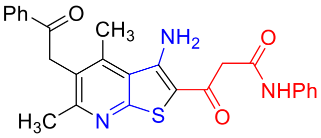

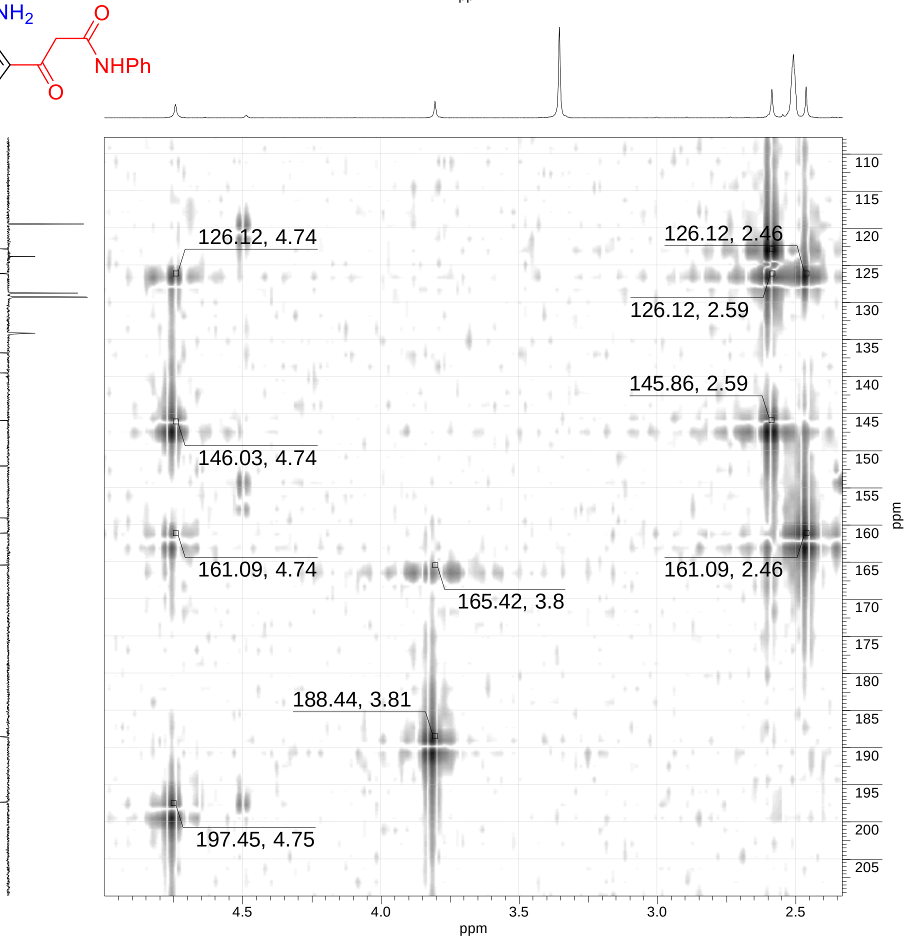

Table S10. The observed correlations in the ^{1}H-^{13}C HMBC 2D NMR spectrum (400/101 MHz, DMSO-d$_6$) of thienopyridine 22f

^{13}C chemical shifts are given in **red**, ^{1}H shifts – in **blue**

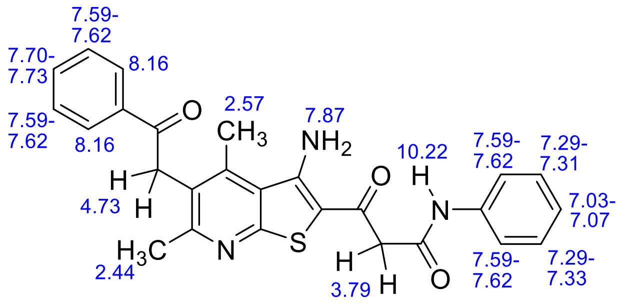

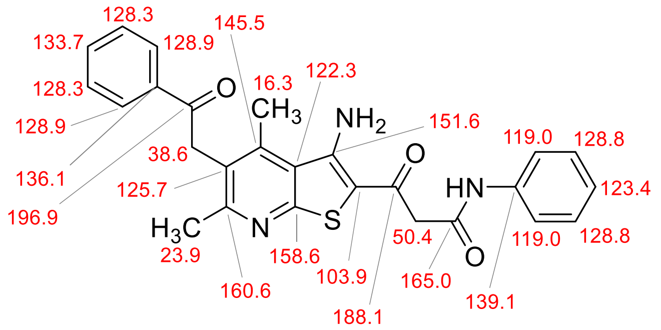

^{1}H NMR shifts, δ, ppm	Correlations in HMBC spectrum, δ, ppm
2.44 (s, 3H, C(6)–CH_3)	125.7 (C-5), 160.6 (C-6)
2.57 (s, 3H, C(4)–CH_3)	122.3 (C-3a), 125.7 (C-5), 145.5 (C-4)
3.79 (s, 2H, $CH_2C(O)$)	165.0 (CONH), 188.1 (C=O)
4.73 (s, 2H, CH_2Bz)	125.7 (C-5), 145.5 (C-4), 160.6 (C-6), 196.9 (C=O Bz)
7.03-7.07 (m, 1H, C(4)H Ph)	119.0* (2C, C-2, C-6 Ph), 128.8* (2C, C-3, C-5 Ph)
7.29-7.33 (m, 2H, C(3)H, C(5)H Ph)	128.8* (2C, C-3, C-5 Ph), 139.1 (C-1 Ph)
7.59-7.62 (m, 4H, Ph, Bz)	119.0* (2C, C-2, C-6 Ph), 123.4* (C-4 Ph), 128.3* (2C, C-3, C-5 Bz), 128.9* (2C, C-2, C-6 Bz), 133.7* (C-4 Bz), 136.3 (C-1 Bz)
7.70-7.73 (m, 1H, C(4)H Bz)	128.3* (2C, C-3, C-5 Bz), 128.9* (2C, C-2, C-6 Bz)
7.87 (br.s, 2H, NH_2)	–
8.16 (d, ^{3}J = 7.3 Hz, 2H, C(2)H, C(6)H Bz)	128.3* (2C, C-3, C-5 Bz), 128.9* (2C, C-2, C-6 Bz), 133.7* (C-4 Bz), 196.9 (C=O Bz)
10.22 (CONH)	119.0* (2C, C-2, C-6 Ph) weak, 165.0 (CONH)

^{1}H NMR spectrum (400 MHz, DMSO-d_6) of thienopyridine 22g

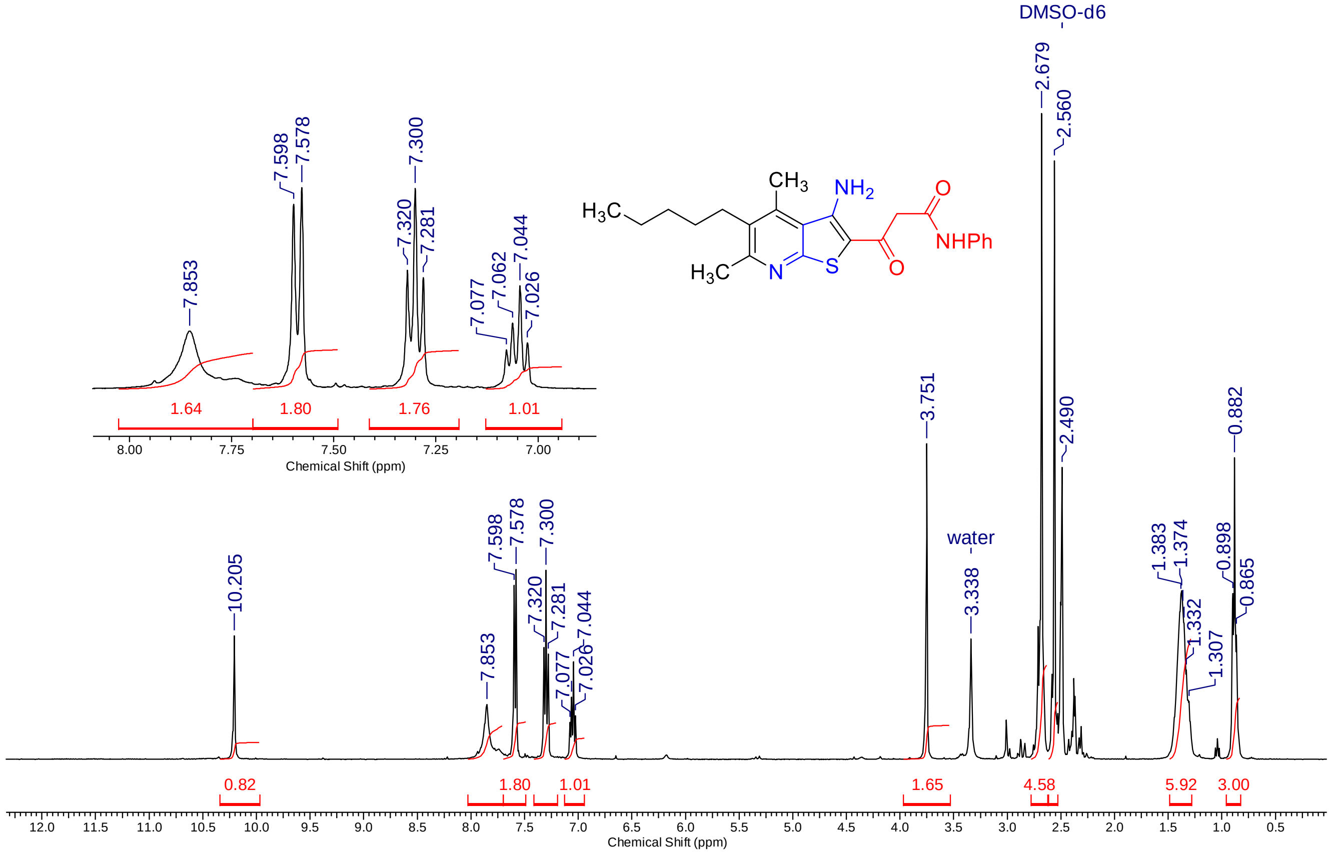

^{13}C DEPTQ NMR spectrum (101 MHz, DMSO-d_6) of thienopyridine 22g

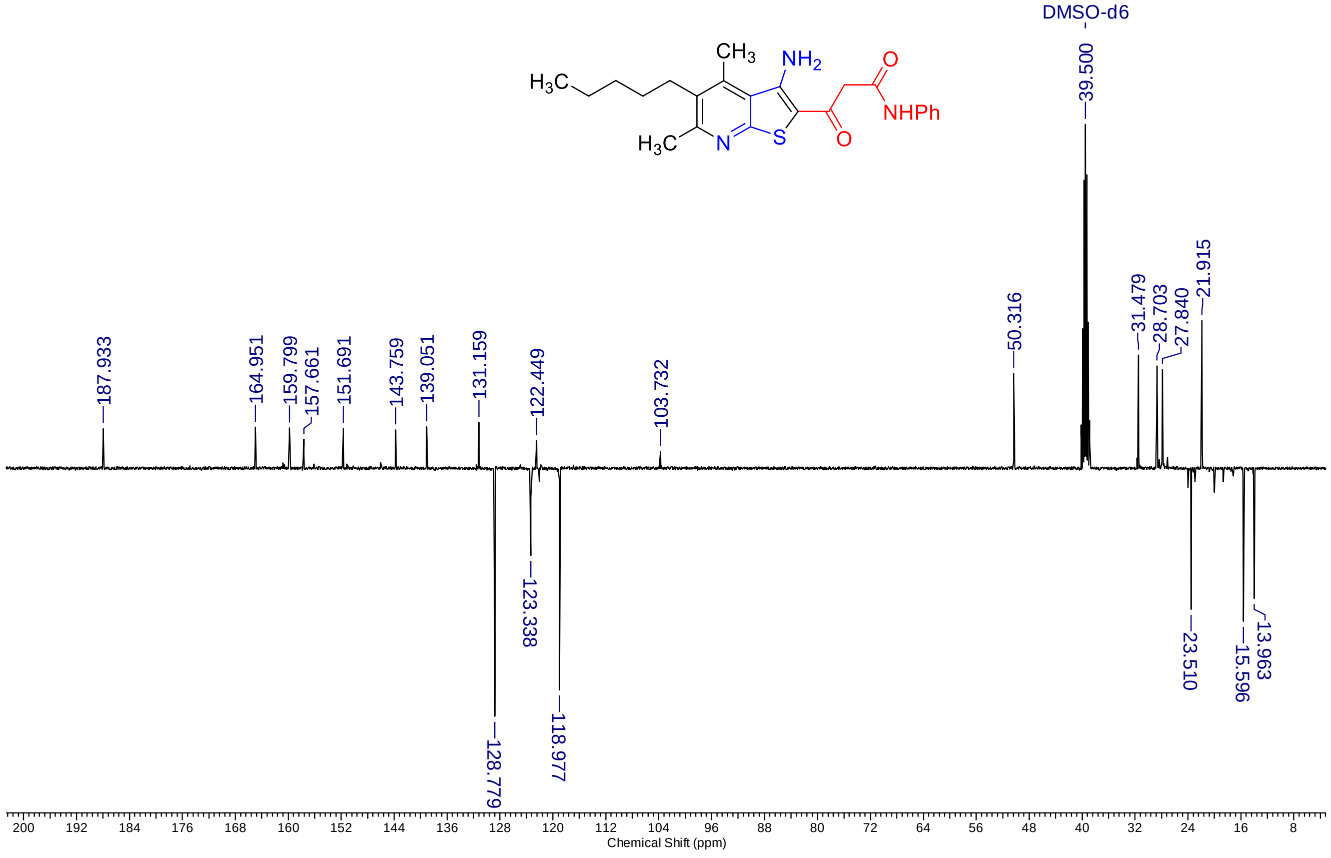

FT-IR spectrum (ATR mode) of thienopyridine 22h

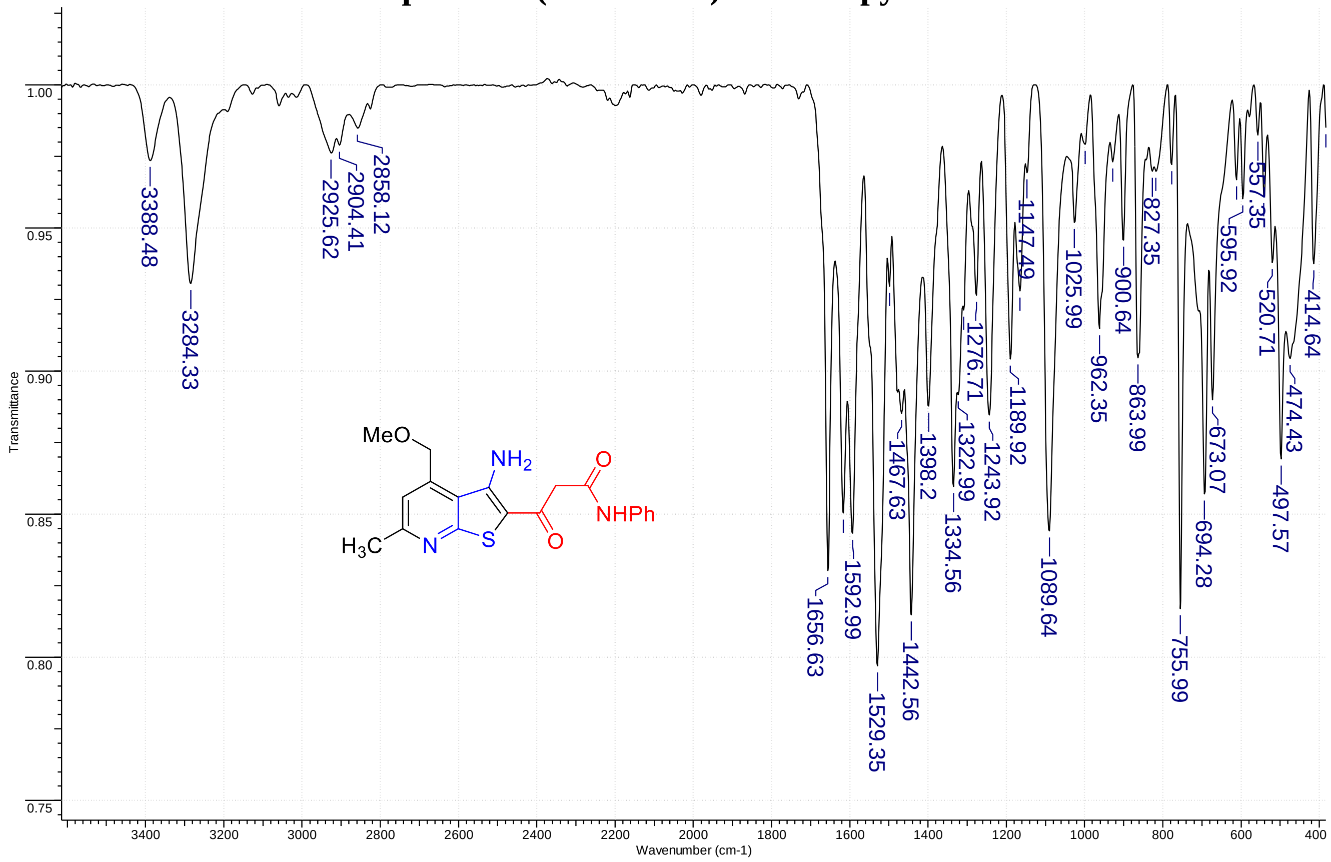

^{1}H NMR spectrum (400 MHz, DMSO-d$_6$) of thienopyridine 22h

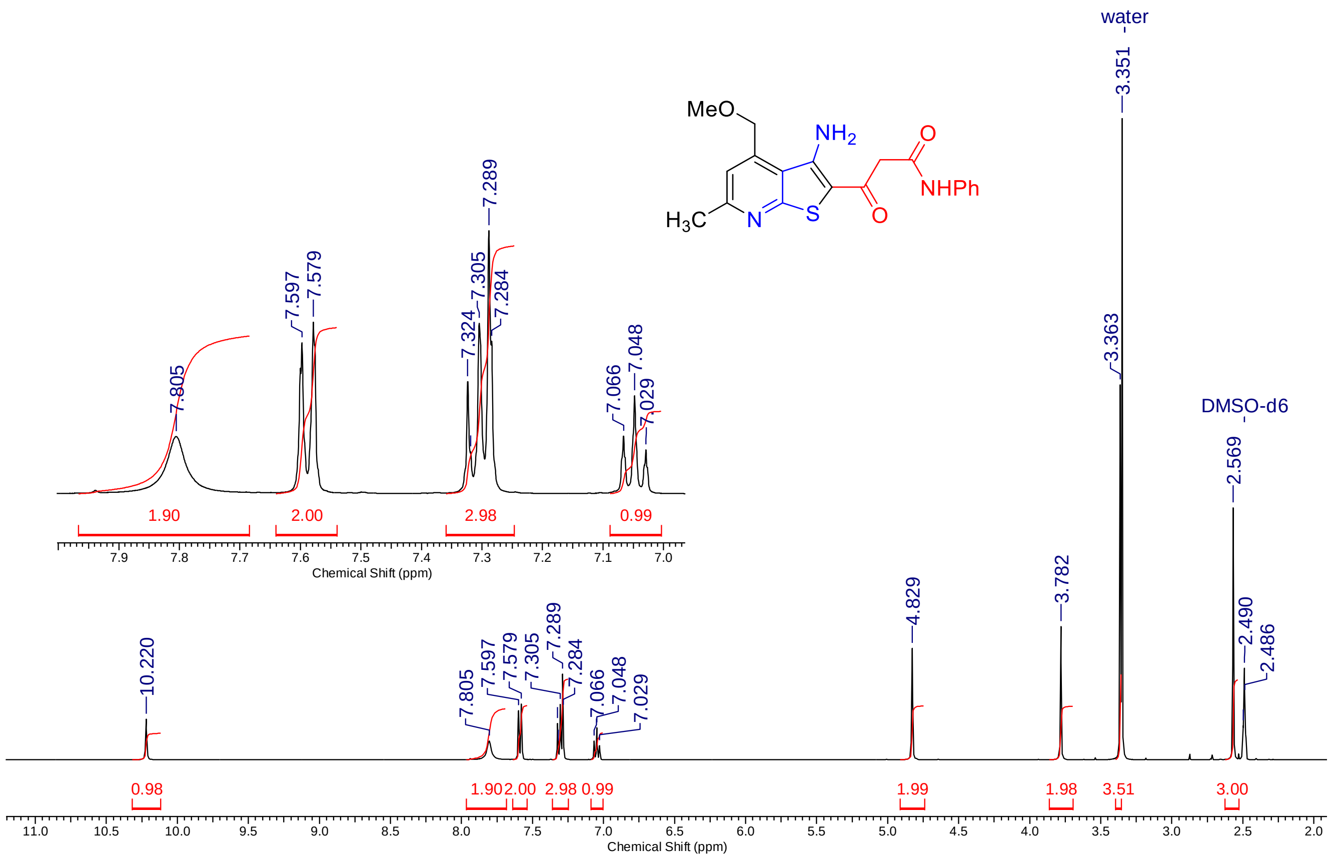

^{13}C DEPTQ NMR spectrum (101 MHz, DMSO-d$_6$) of thienopyridine 22h

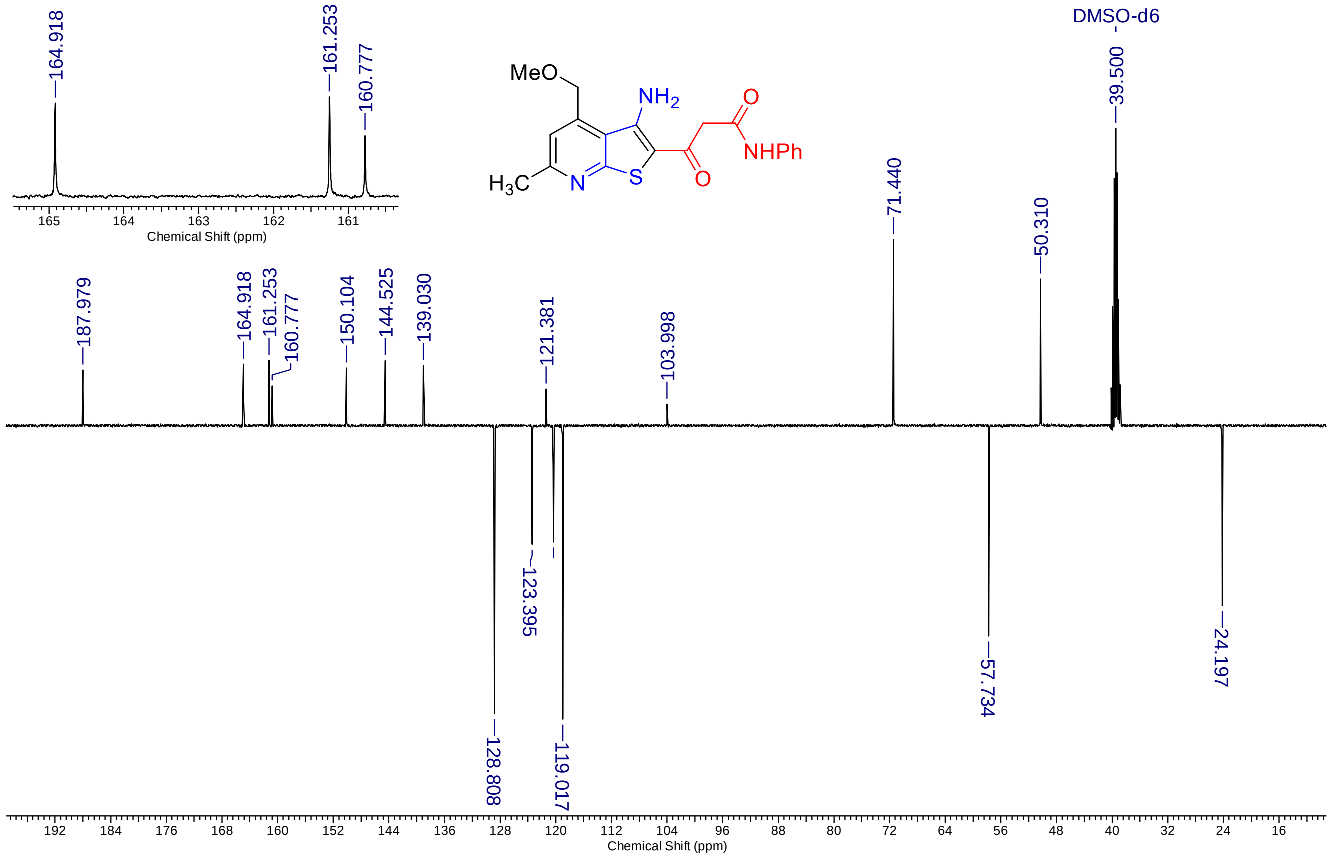

FT-IR spectrum (ATR mode) of thienopyridine 23

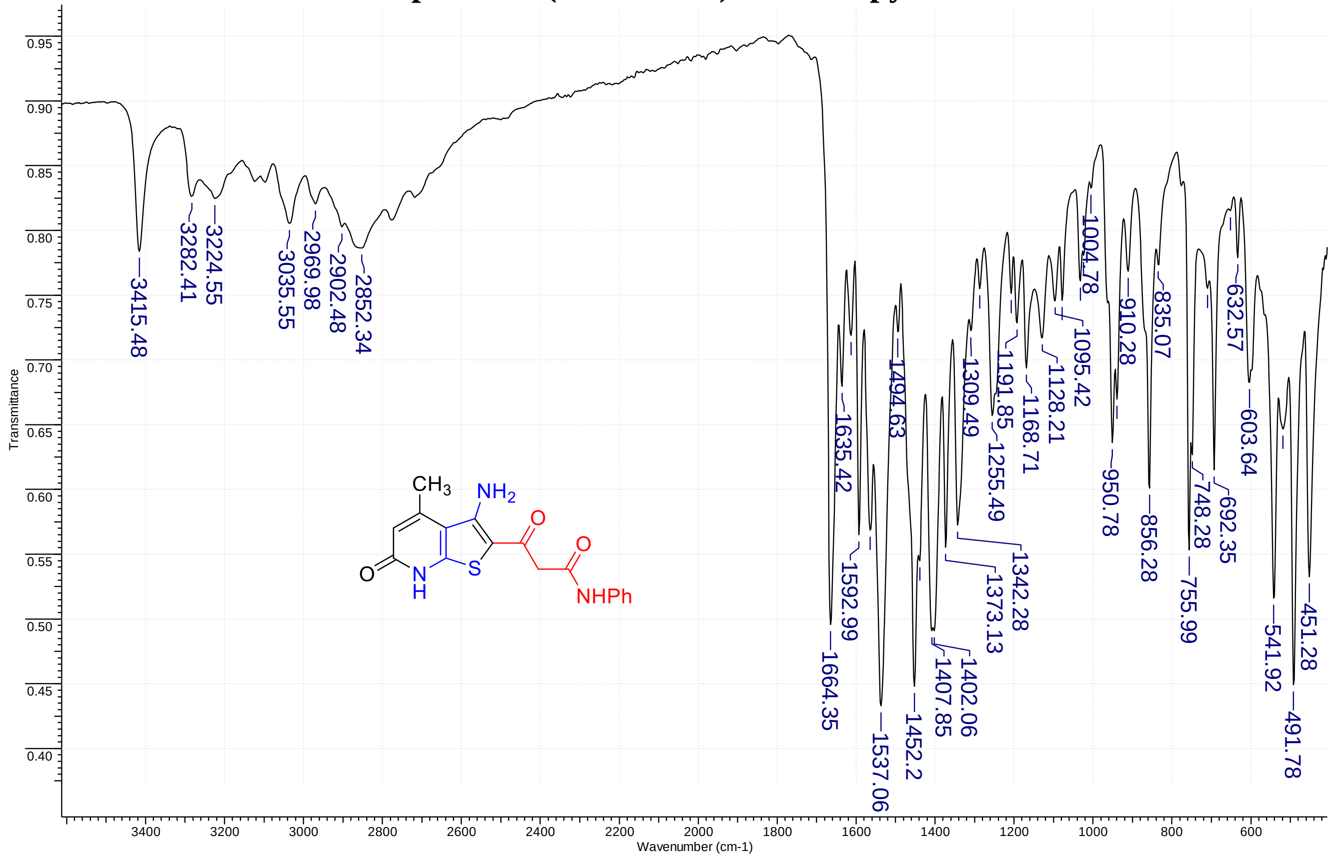

^{1}H NMR spectrum (400 MHz, DMSO-d$_6$) of thienopyridine 23

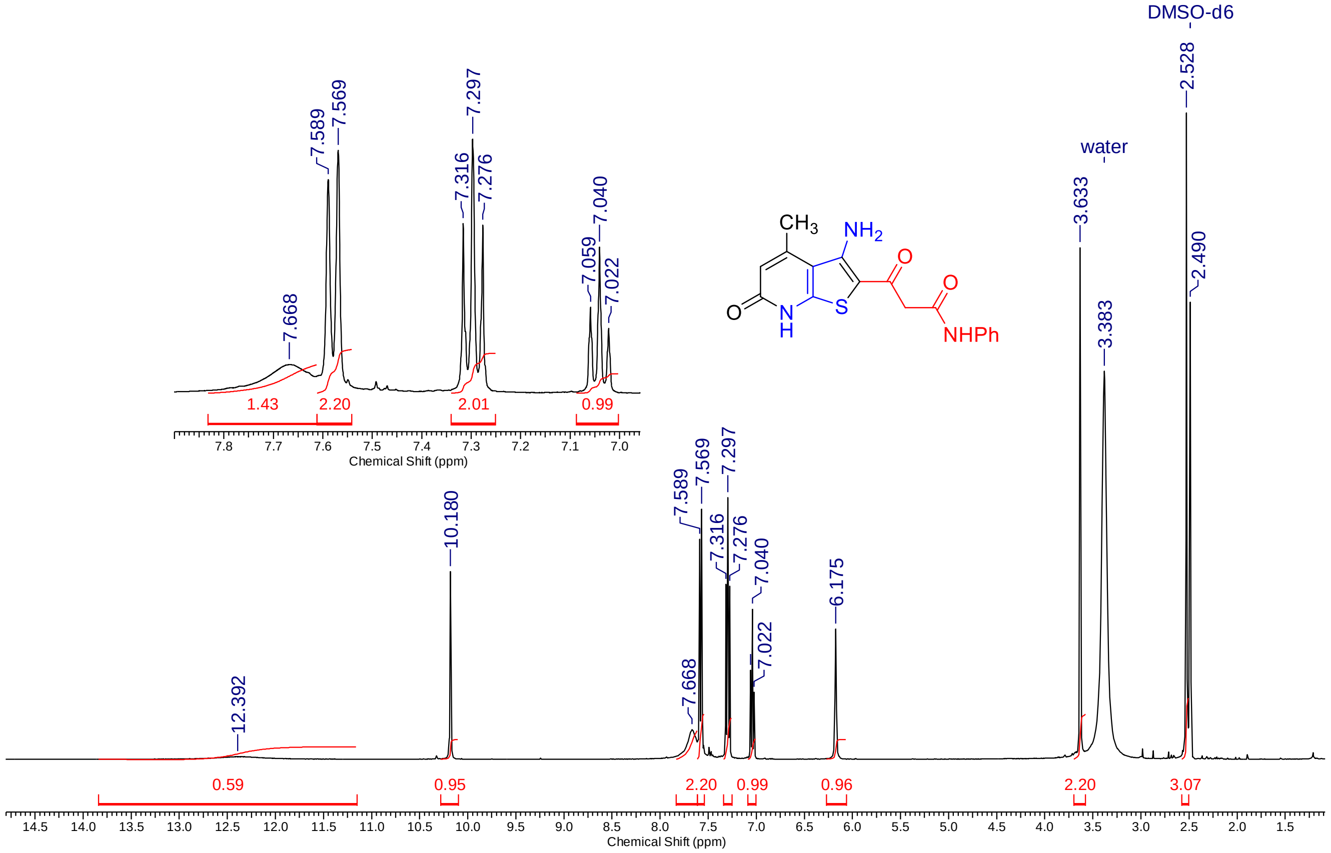

^{13}C DEPTQ NMR spectrum (101 MHz, DMSO-d$_6$) of thienopyridine 23

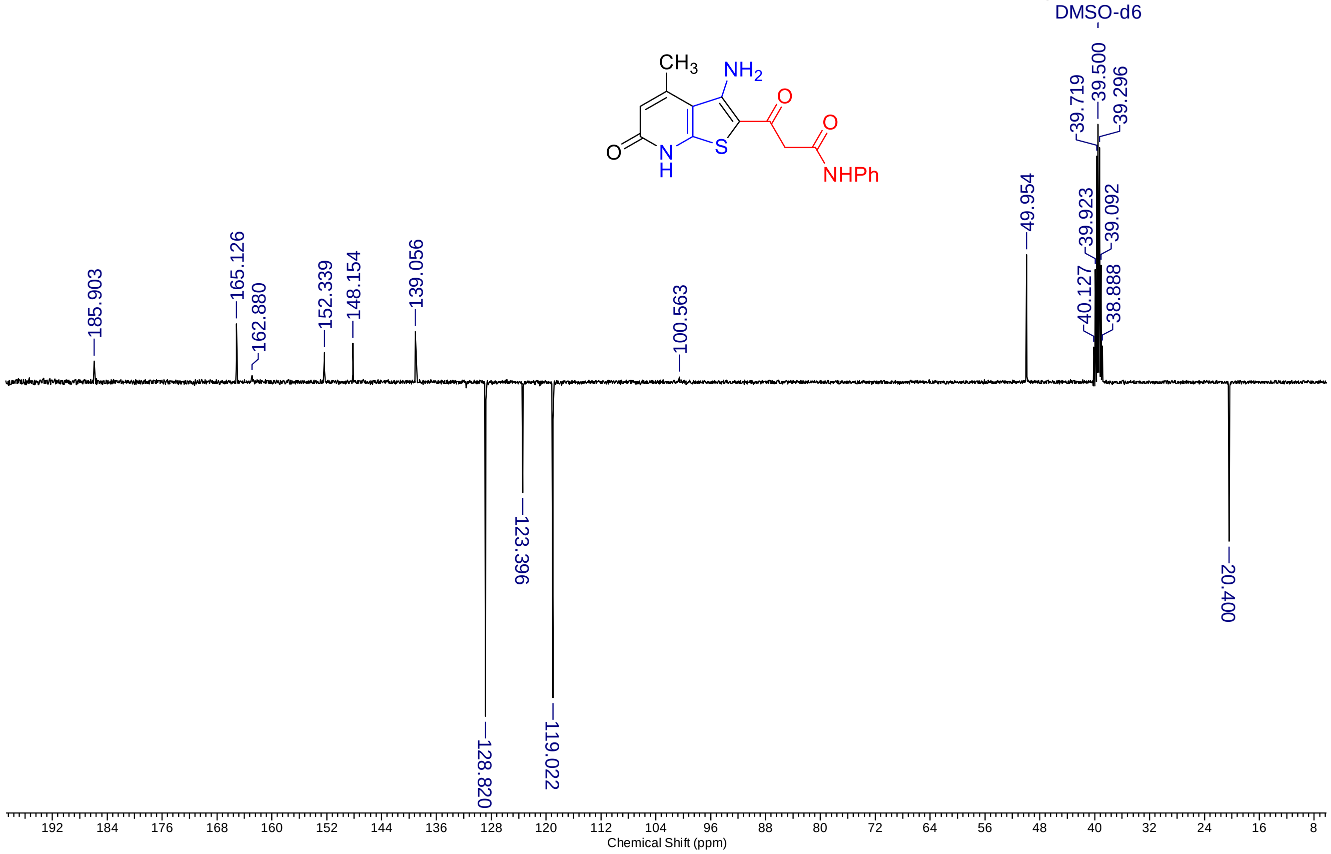

FT-IR spectrum (Nujol mulls) of the crude ketoamide intermediate 20d isolated

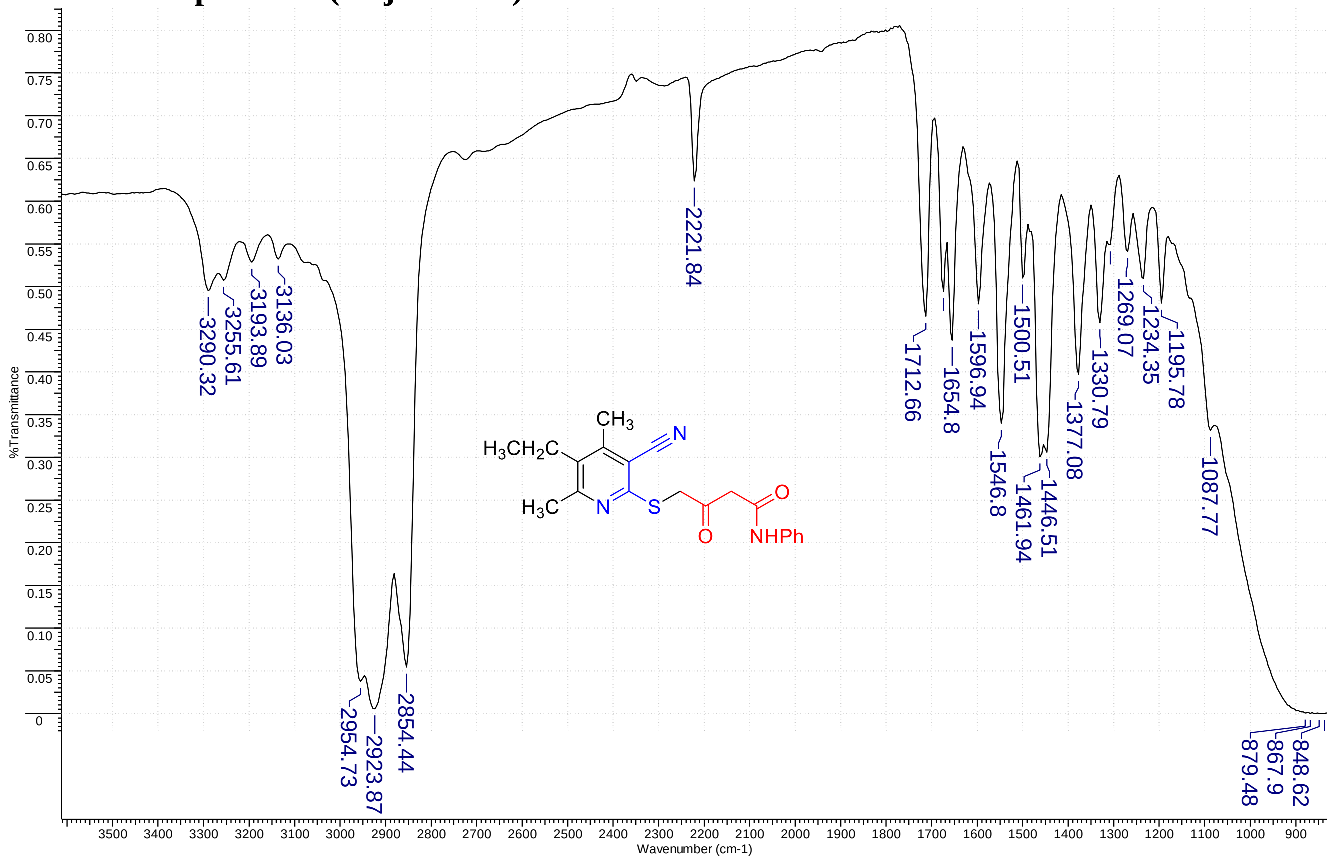

^{1}H NMR spectrum (400 MHz, DMSO-d6) of the crude ketoamide intermediate 20d isolated

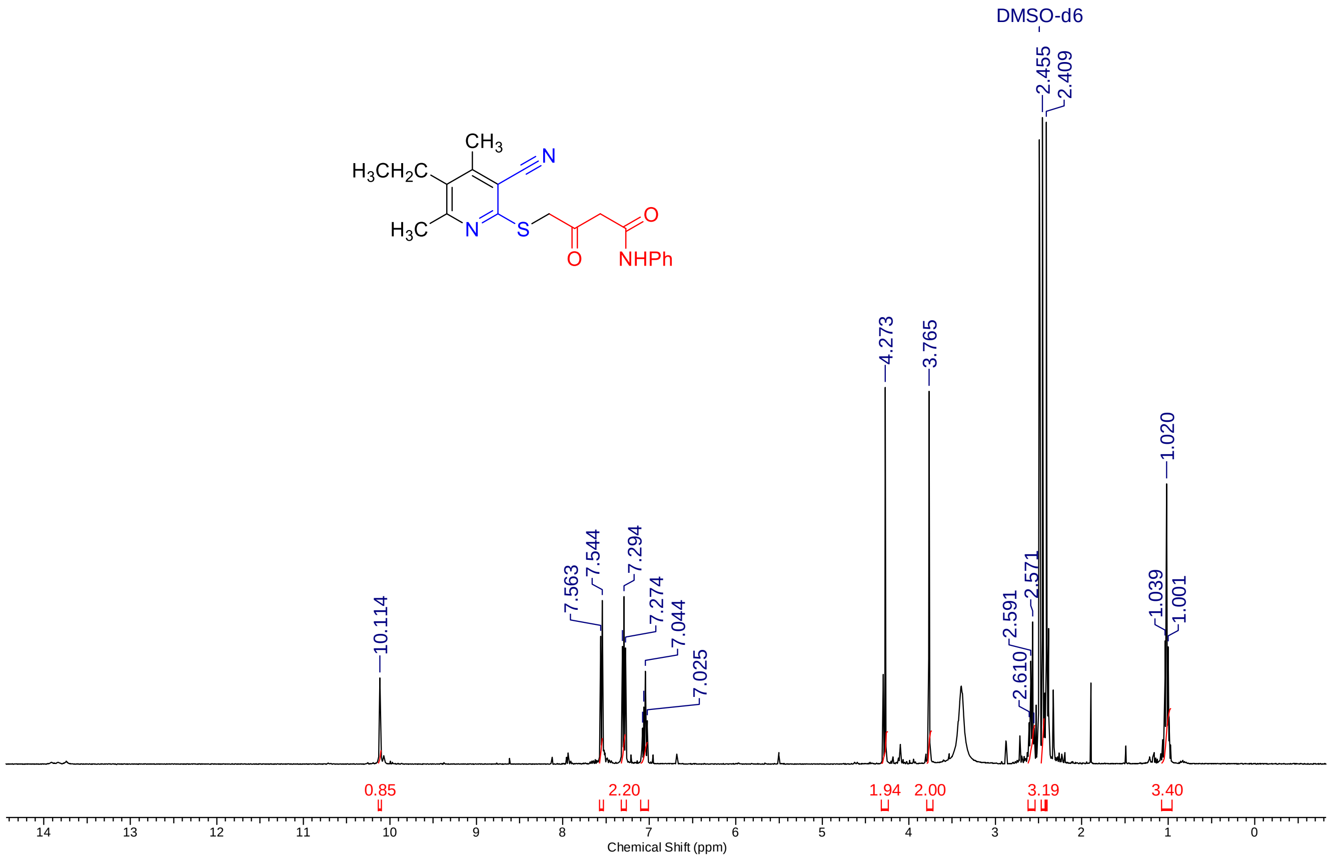

^{13}C DEPTQ NMR spectrum (101 MHz, DMSO-d$_6$) of the crude intermediate 20d isolated

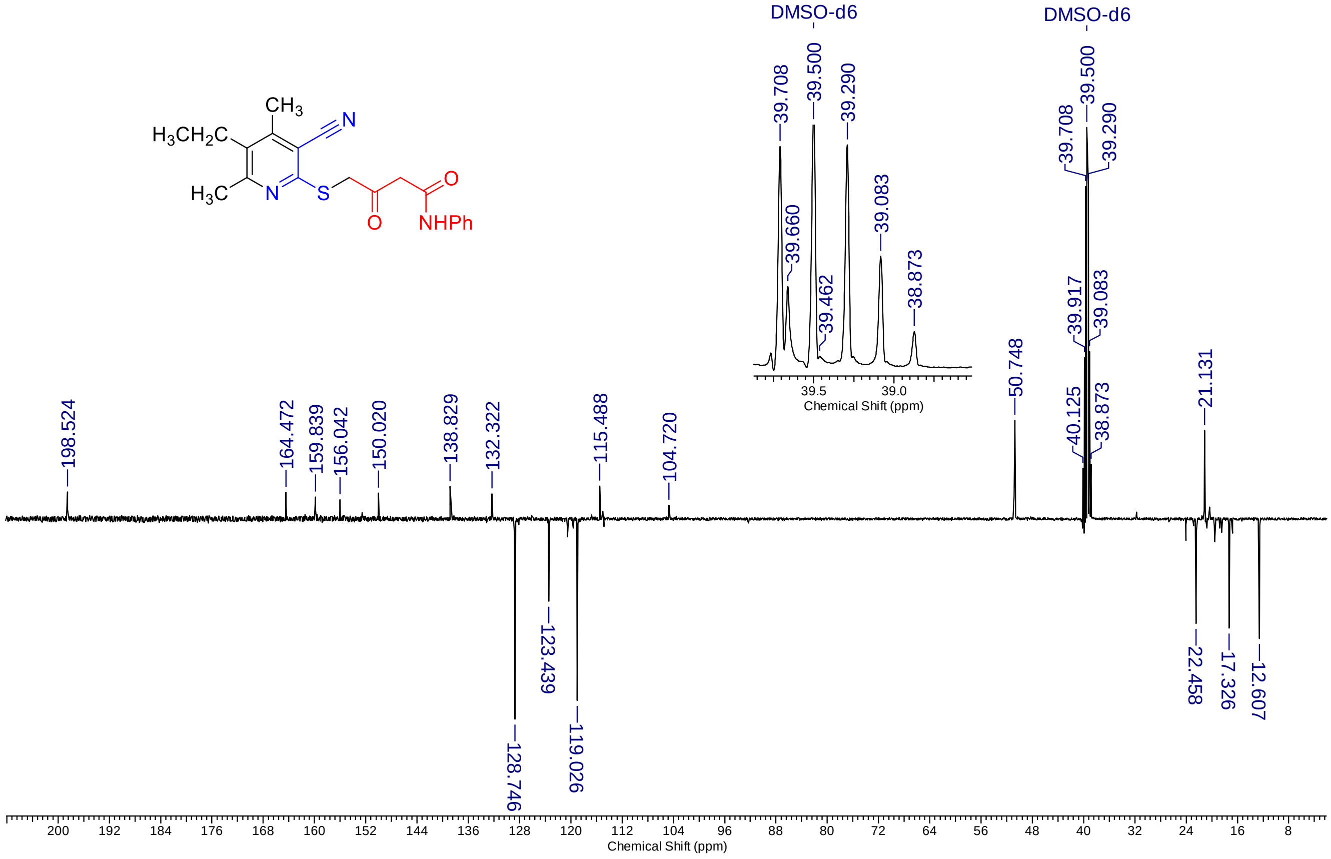

FT-IR spectrum (Nujol mulls) of 4,5,6,7-tetrahyrdothieno[2,3-b]pyridine 24a

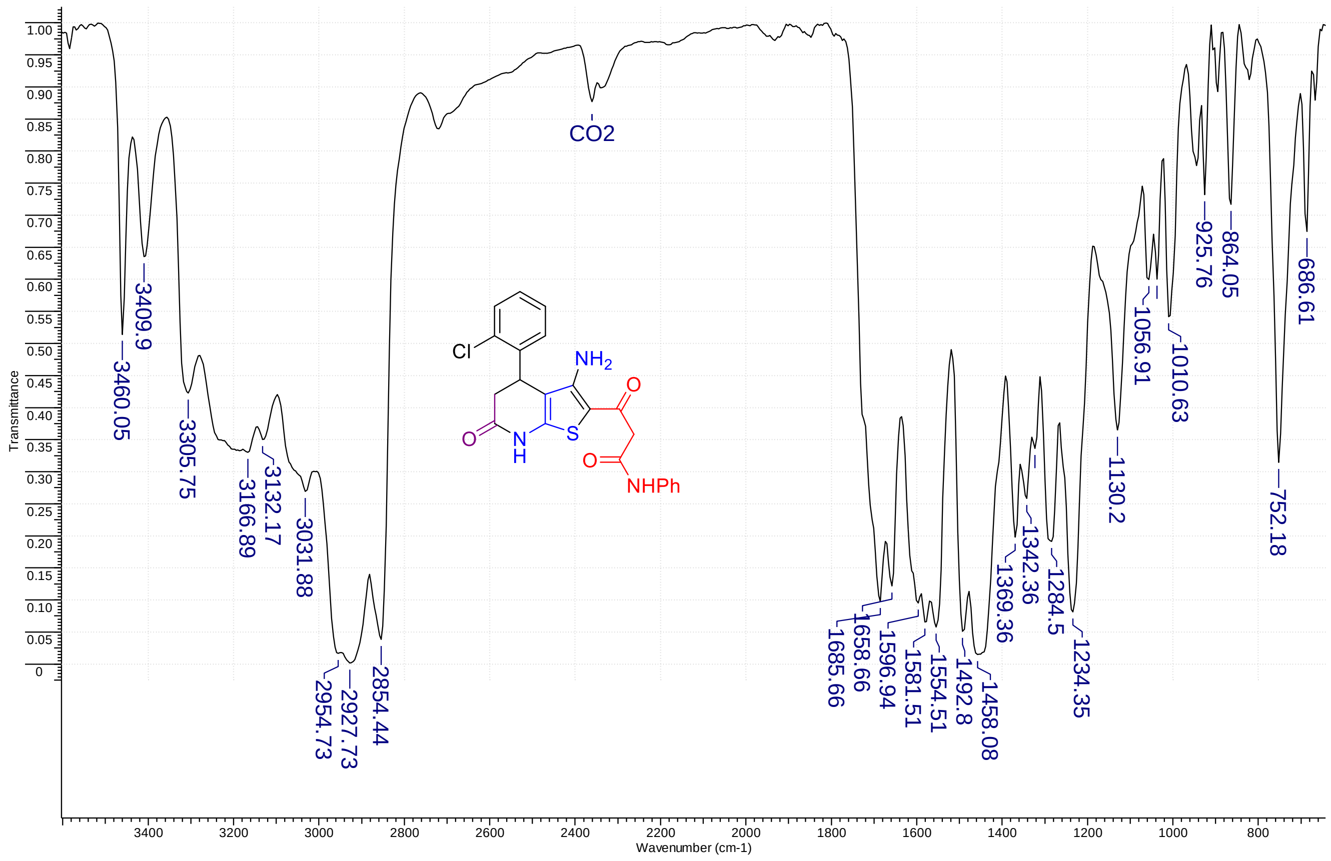

^{1}H NMR spectrum (400 MHz, DMSO-d$_6$) of 4,5,6,7-tetrahyrdothieno[2,3-b]pyridine 24a

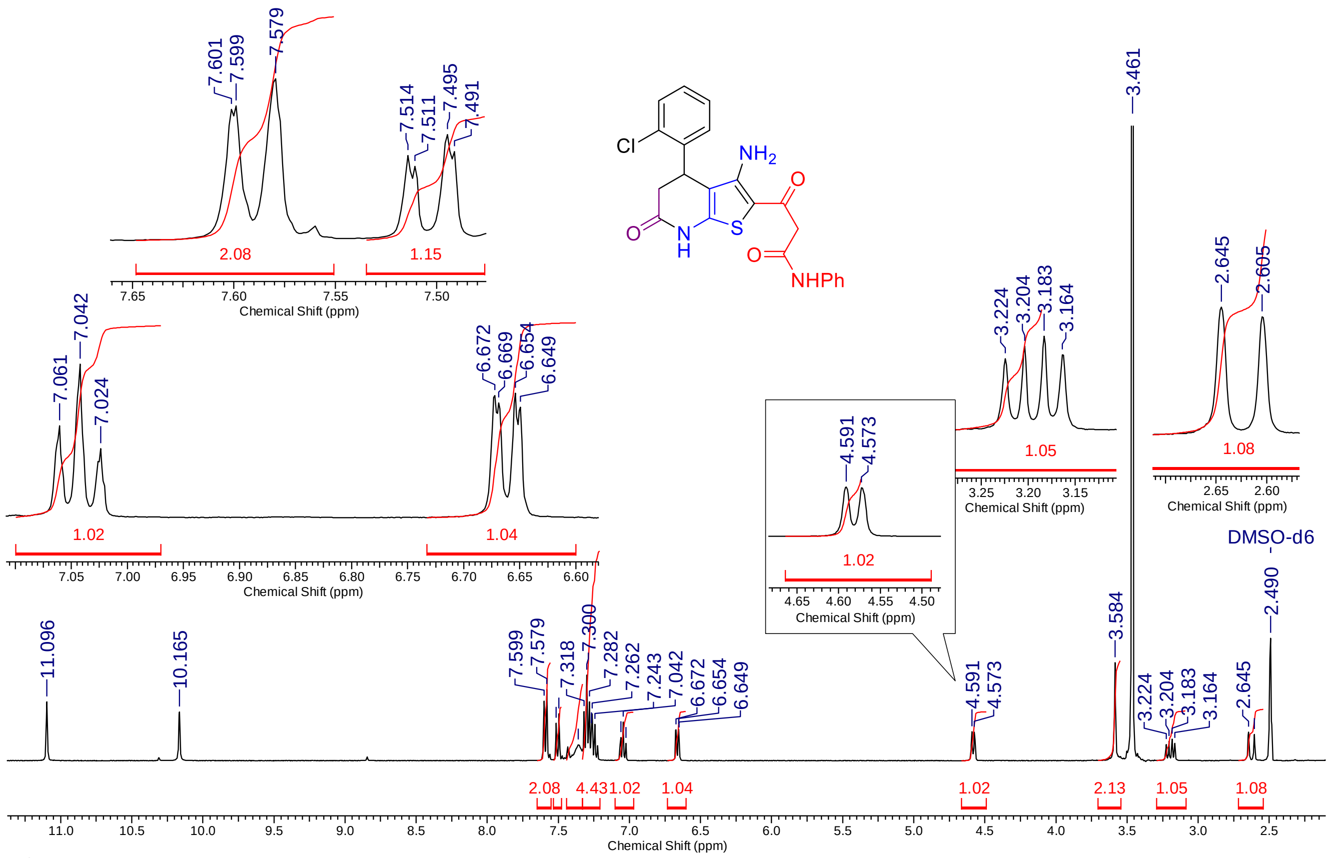

^{13}C NMR spectrum (101 MHz, DMSO-d$_6$) of 4,5,6,7-tetrahyrdothieno[2,3-b]pyridine 24a

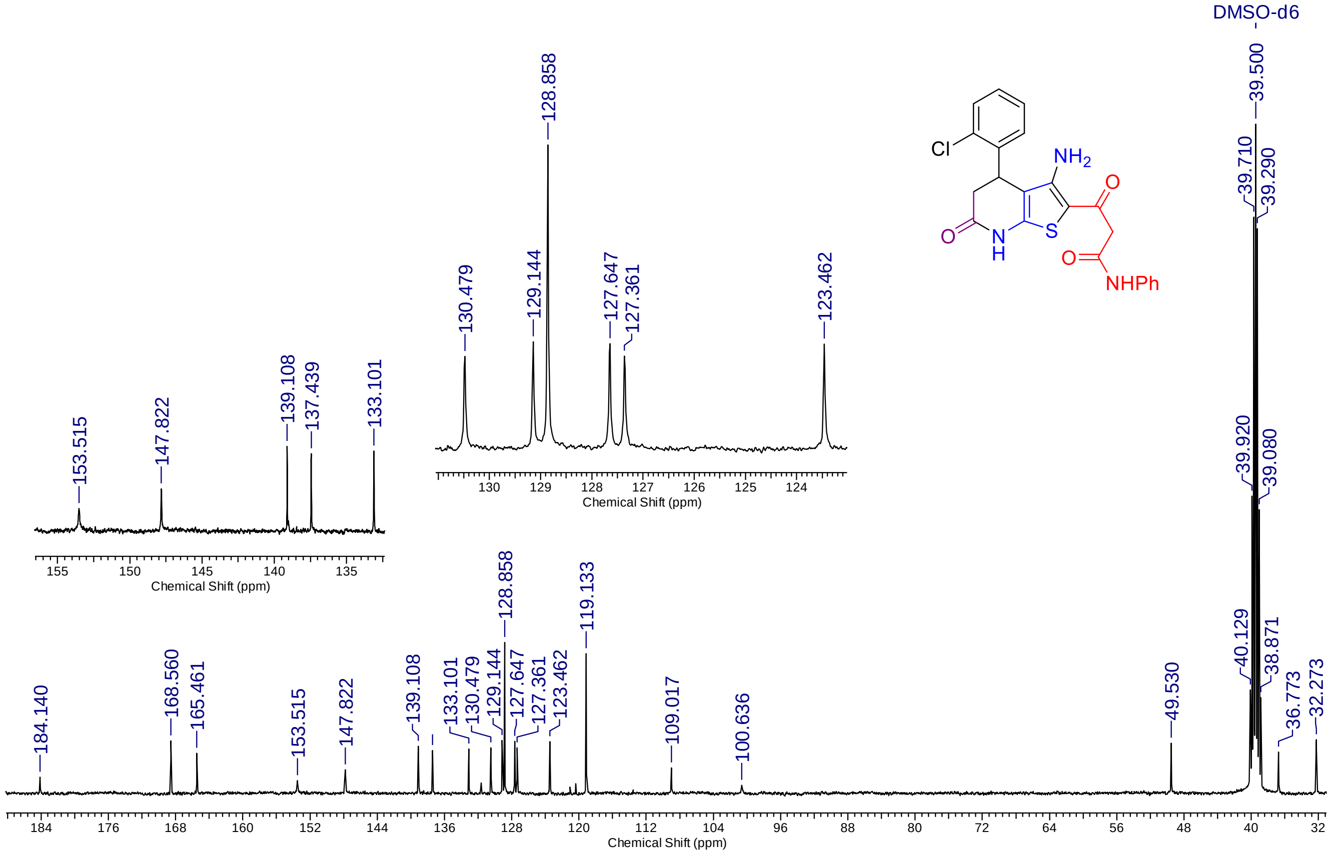

FT-IR spectrum (Nujol mulls) of 4,5,6,7-tetrahyrdothieno[2,3-b]pyridine 24b

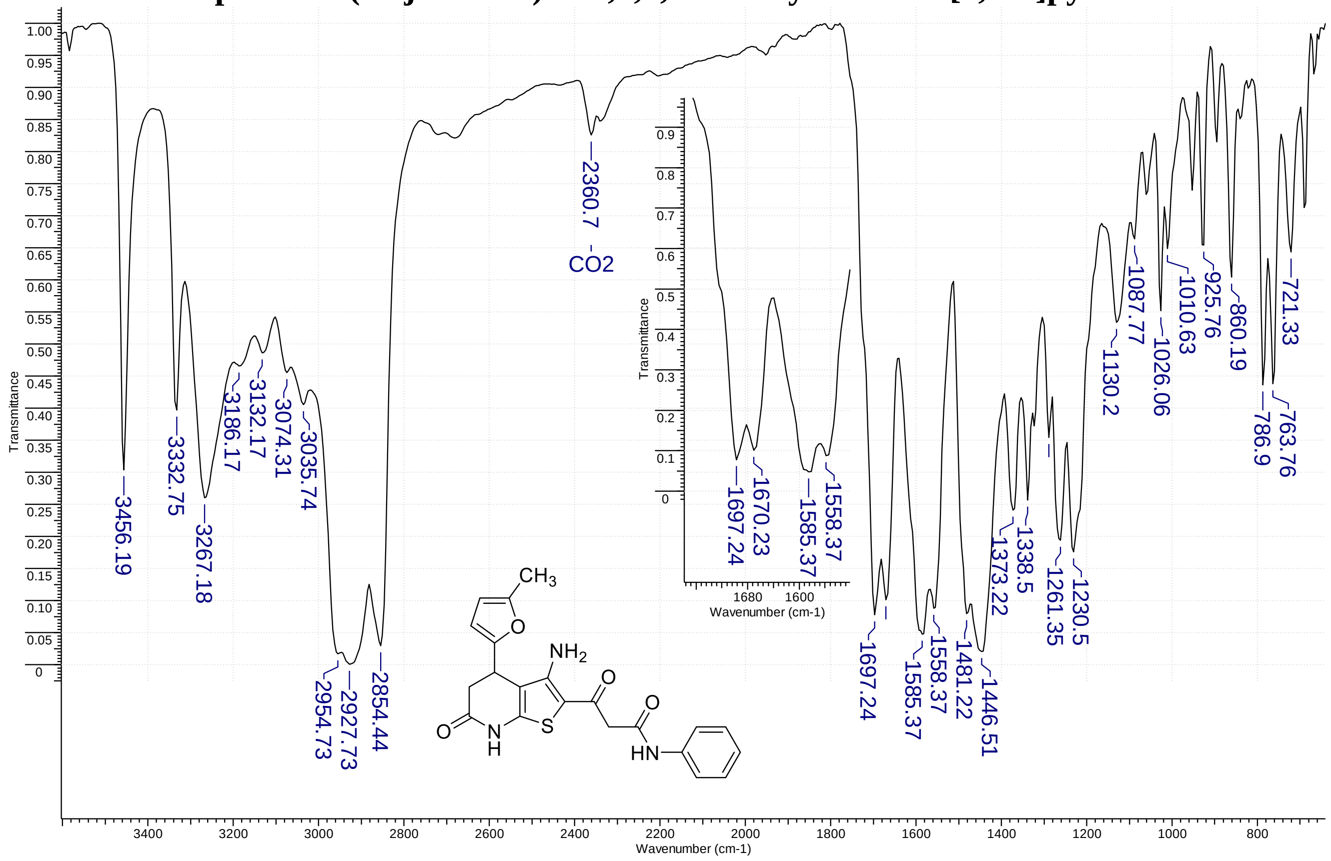

^{1}H NMR spectrum (400 MHz, DMSO-d$_6$) of 4,5,6,7-tetrahyrdothieno[2,3-b]pyridine 24b

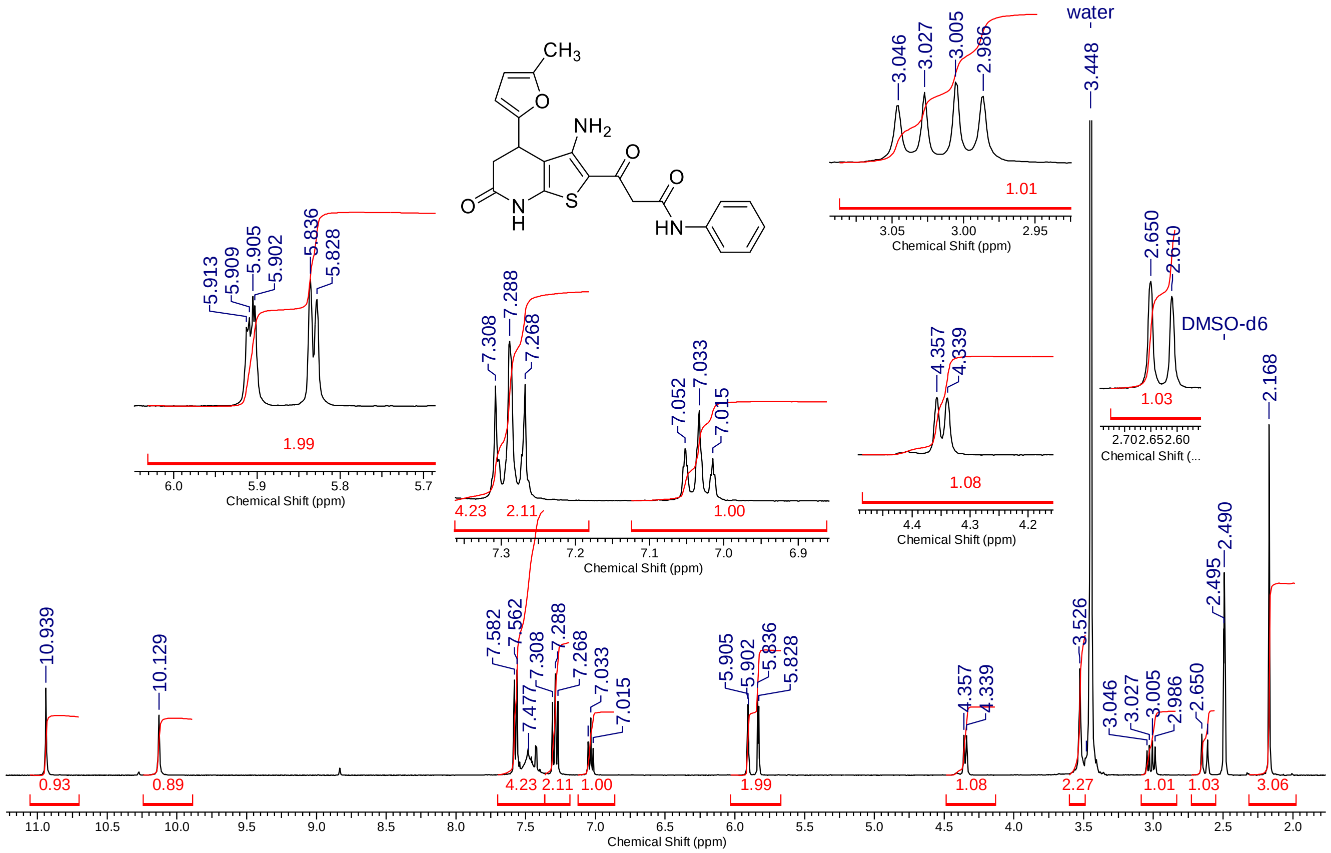

^{13}C NMR spectrum (101 MHz, DMSO-d$_6$) of 4,5,6,7-tetrahyrdothieno[2,3-b]pyridine 24b

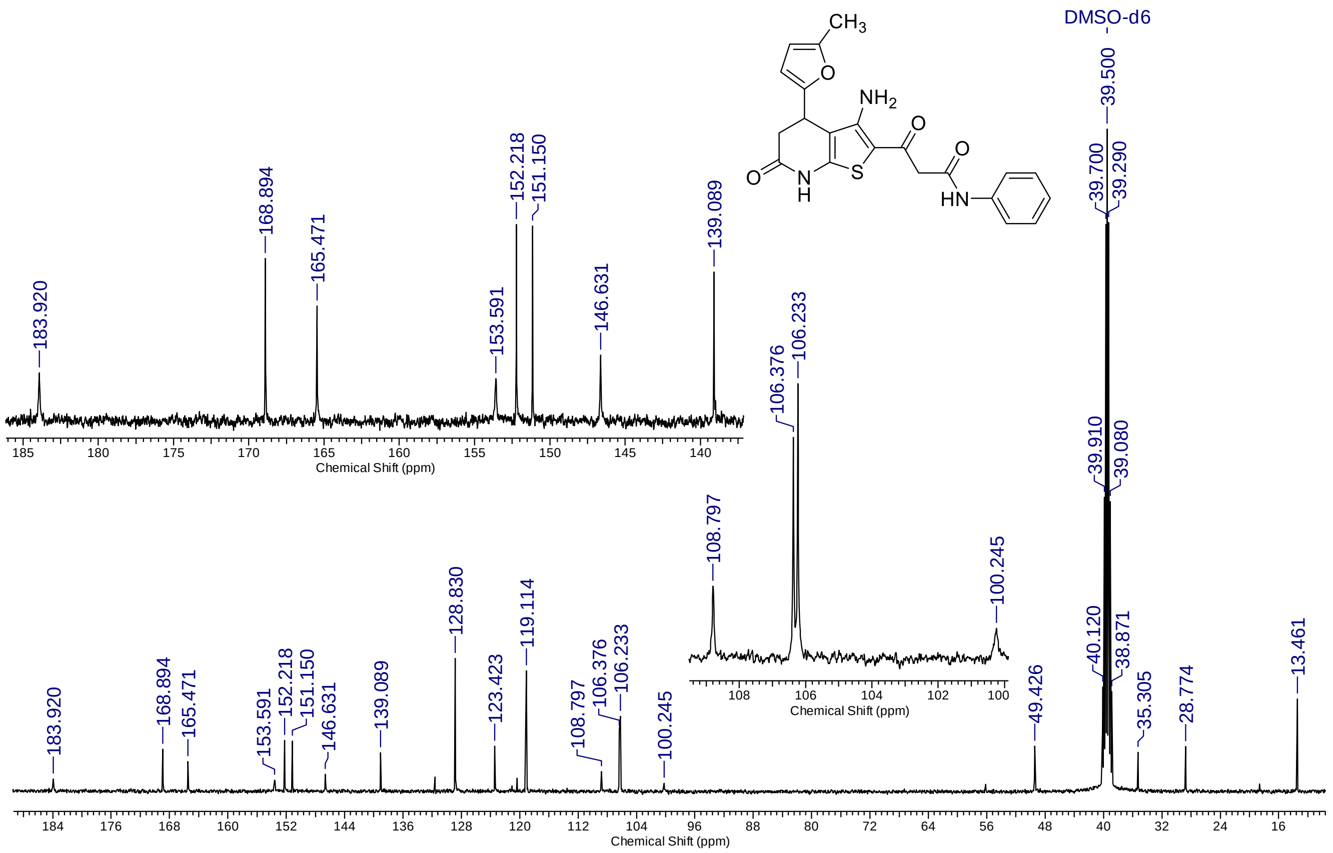

FT-IR spectrum (Nujol mulls) of thiazolyl acetanilide 25

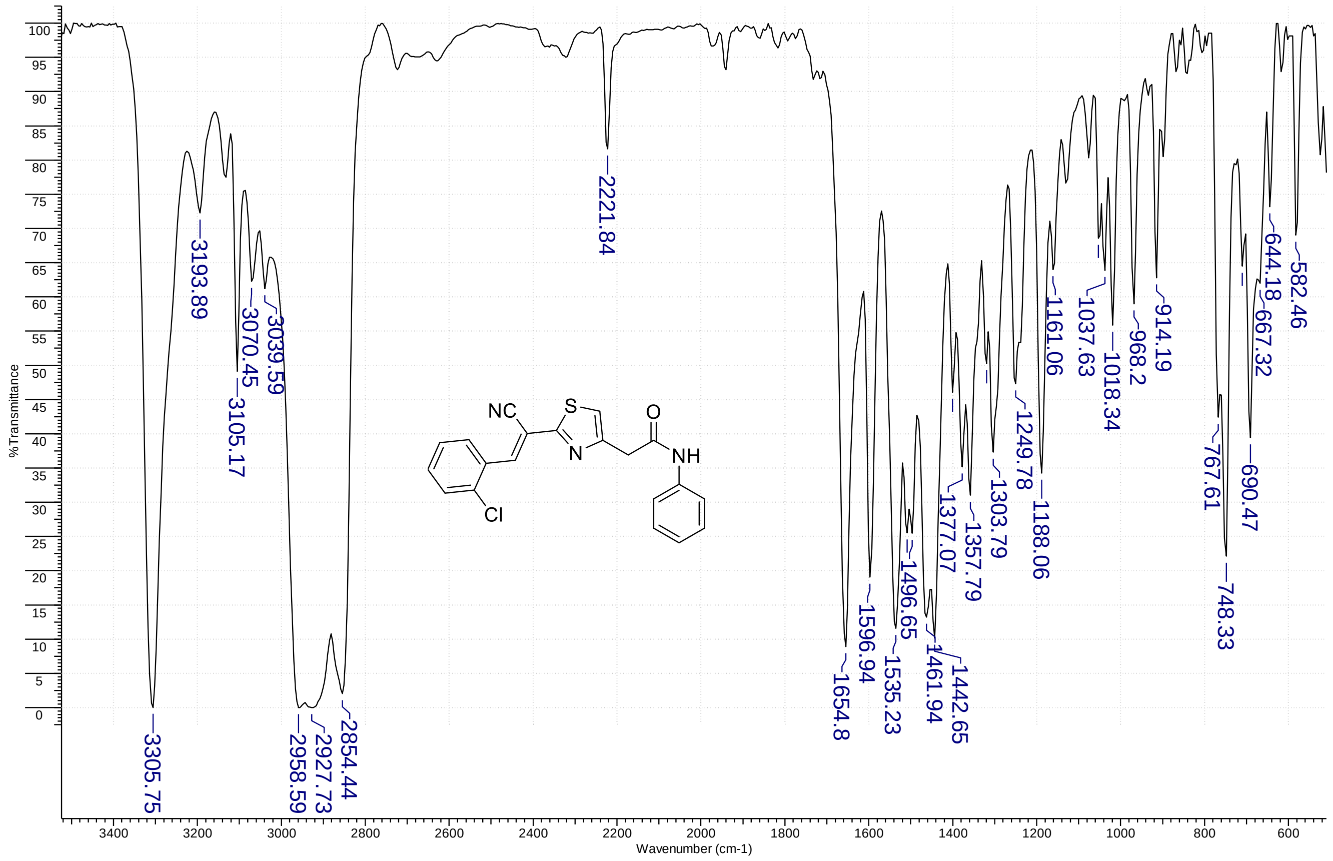

^{1}H NMR spectrum (400 MHz, DMSO-d$_6$) of thiazolyl acetanilide 25

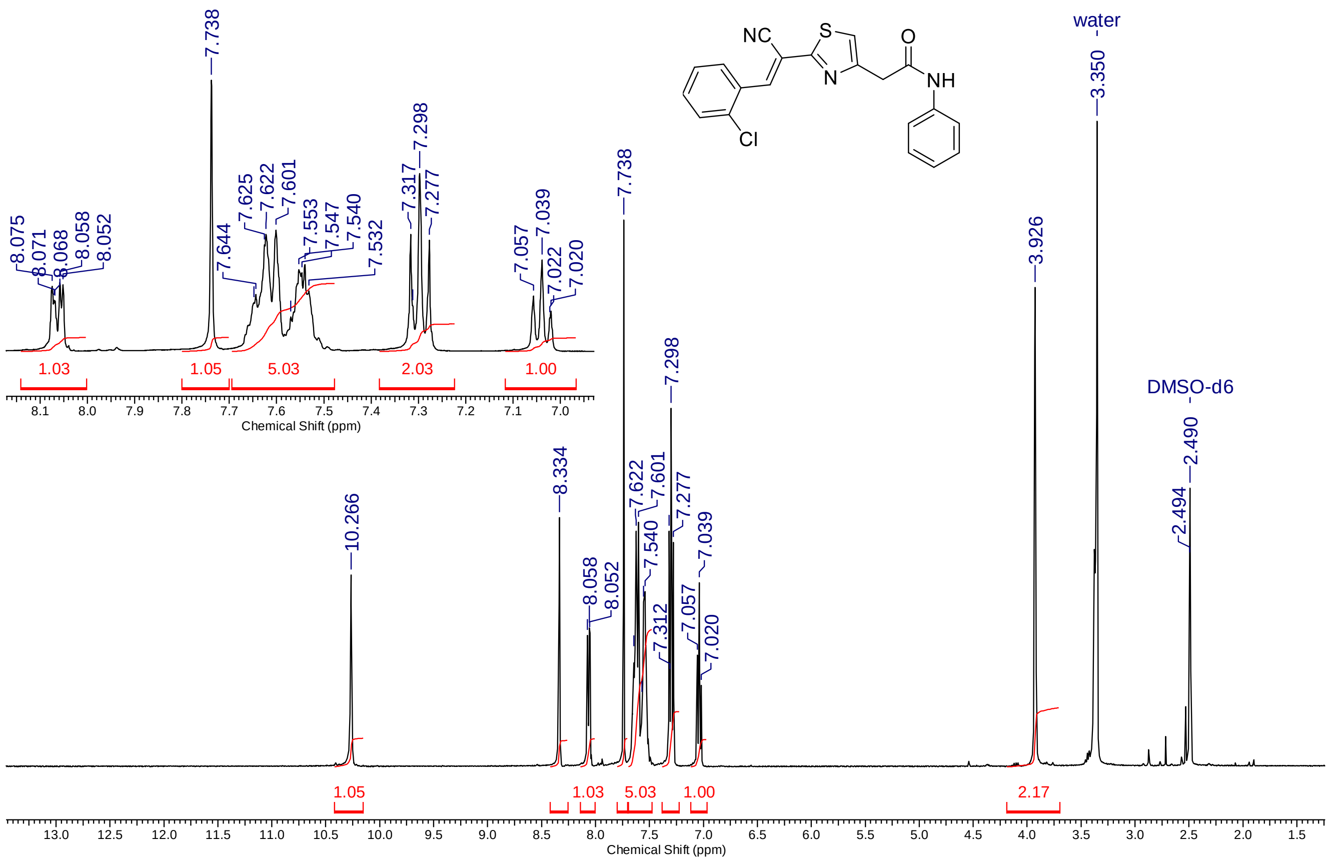

^{13}C DEPTQ NMR spectrum (101 MHz, DMSO-d$_6$) of thiazolyl acetanilide 25

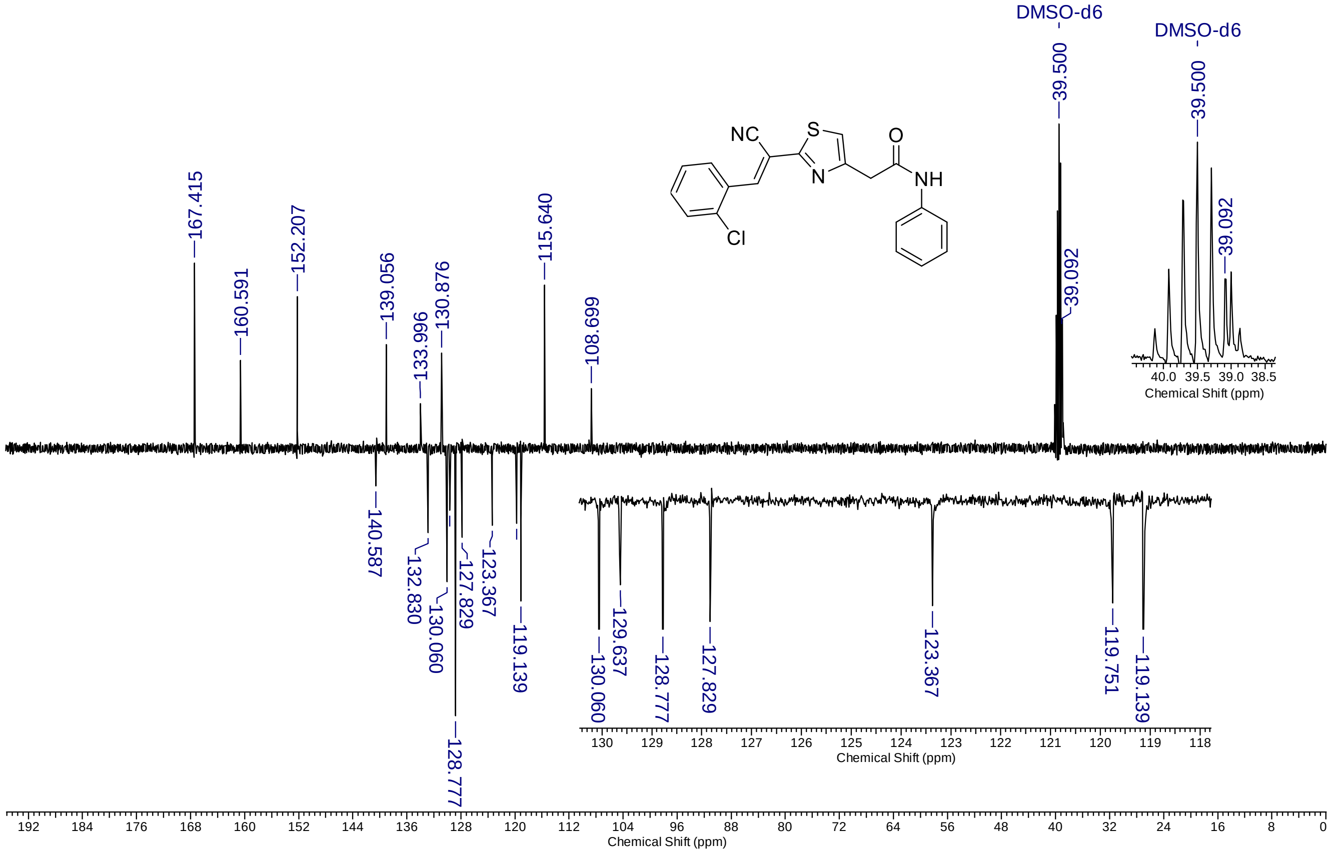

^{1}H NMR spectrum (400 MHz, DMSO-d$_6$) of the mixture product prepared by reaction of thienopyridine β-ketoamide 22a with triethyl orthoformate. Signals of dipyridothiophene 33 are highlighted with red circles; signals of the starting 22a are marked with green diamonds.

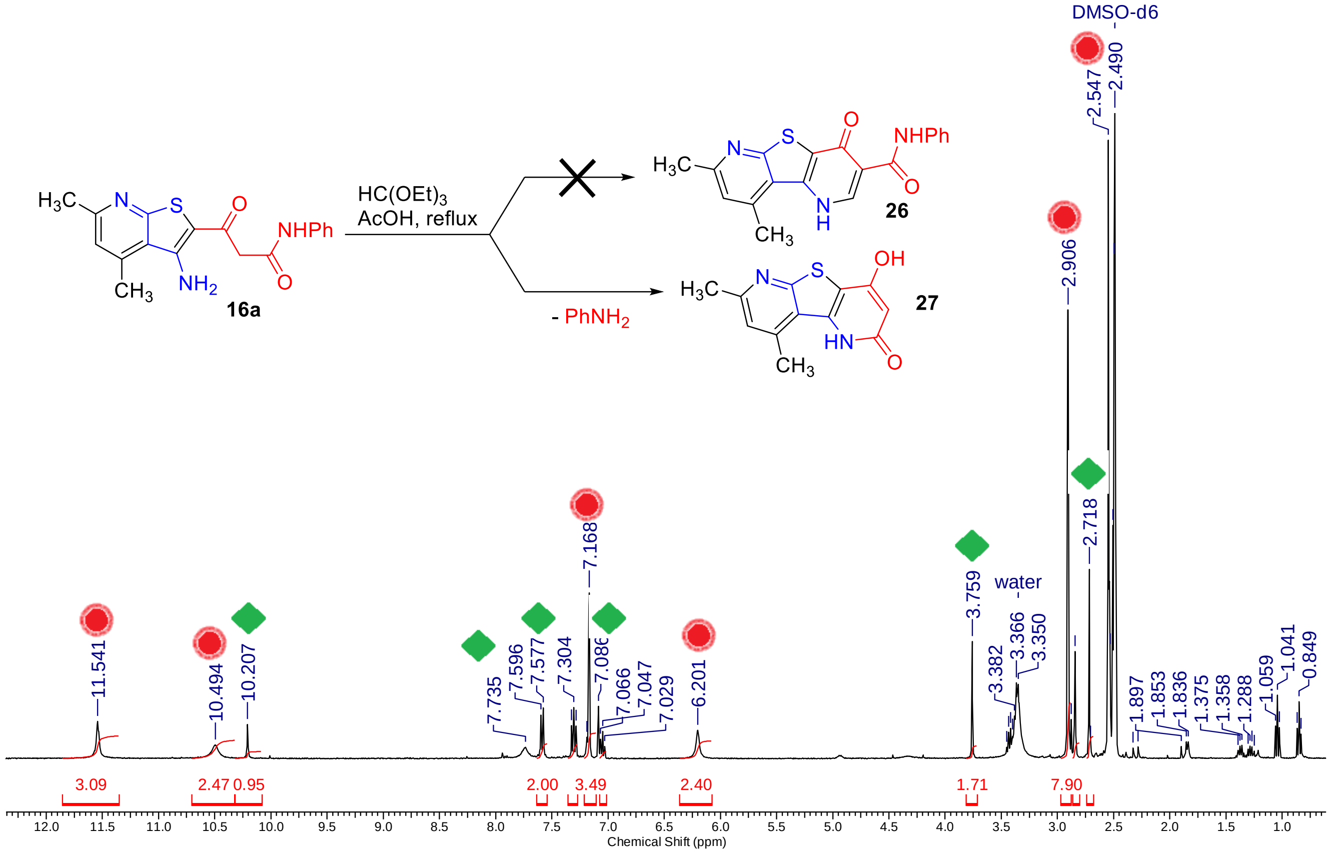

^{1}H NMR spectrum (400 MHz, DMSO-d$_6$) of 4-hydroxy-7,9-dimethylthieno[2,3-*b*:4,5-*b*']dipyridine-2(1*H*)-one 33

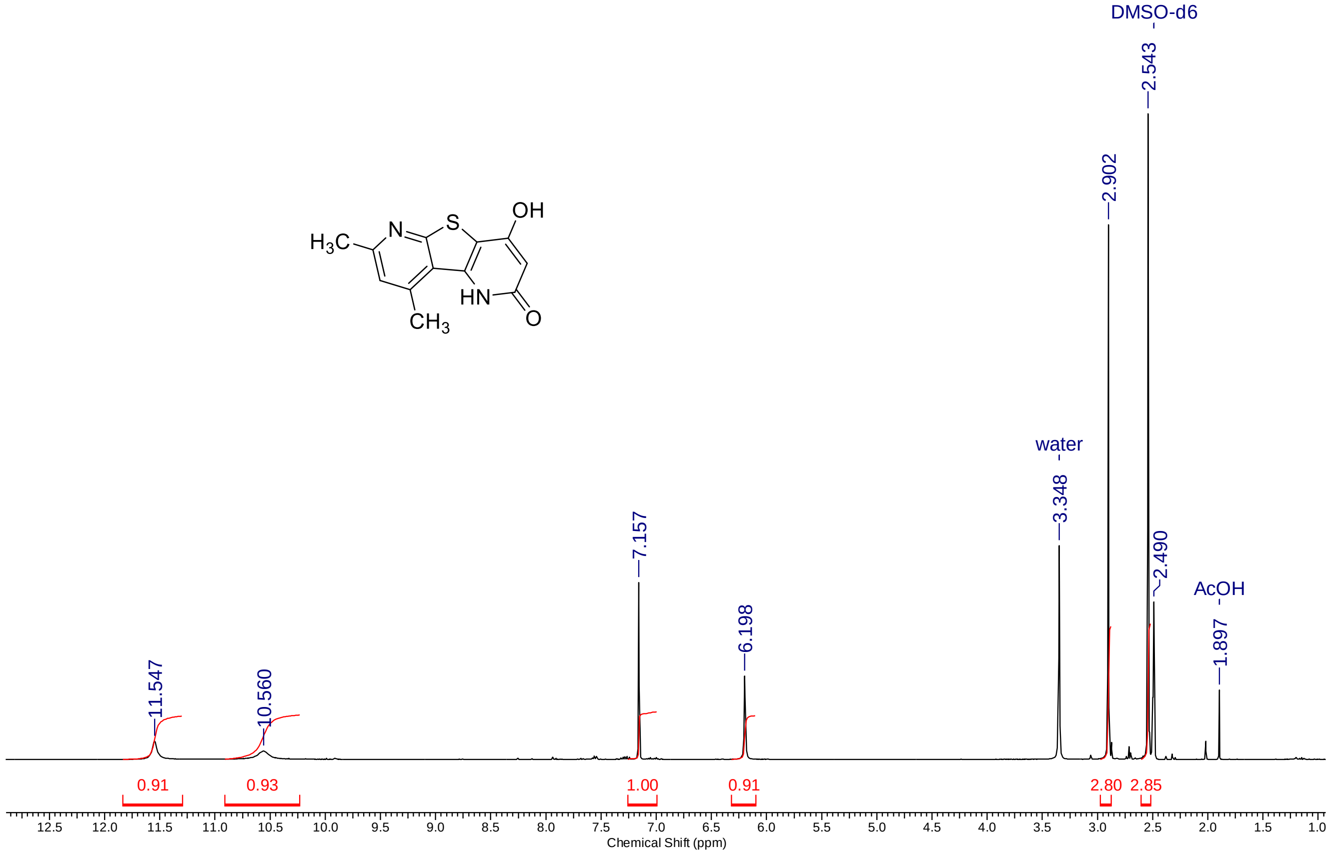

^{13}C DEPTQ NMR spectrum (101 MHz, DMSO-d$_6$) of 4-hydroxy-7,9-dimethylthieno[2,3-*b*:4,5-*b'*]dipyridine-2(1*H*)-one 33

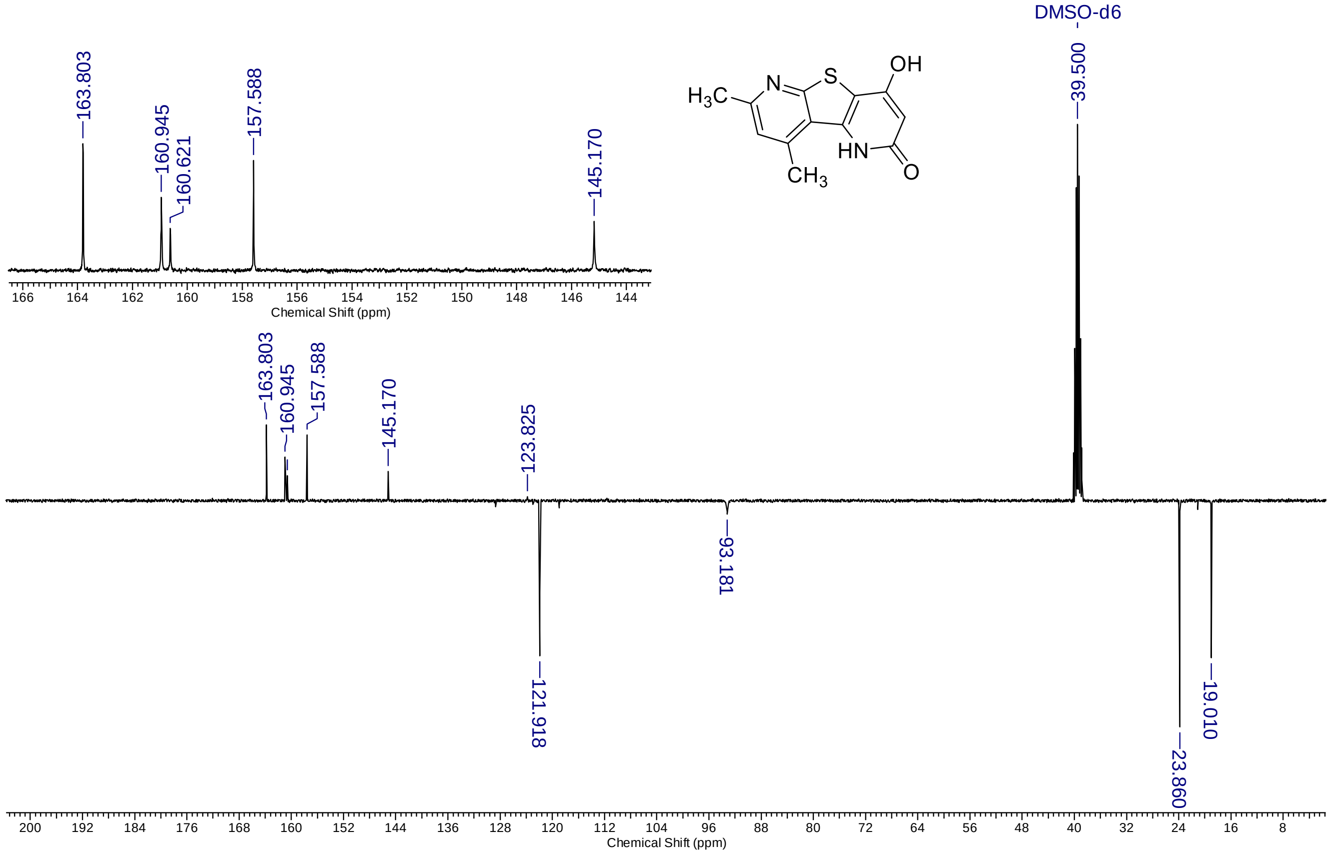

FT-IR spectrum (ATR mode) of 4-hydroxy-7,9-dimethylthieno[2,3-*b*:4,5-*b'*]dipyridine-2(1*H*)-one 33

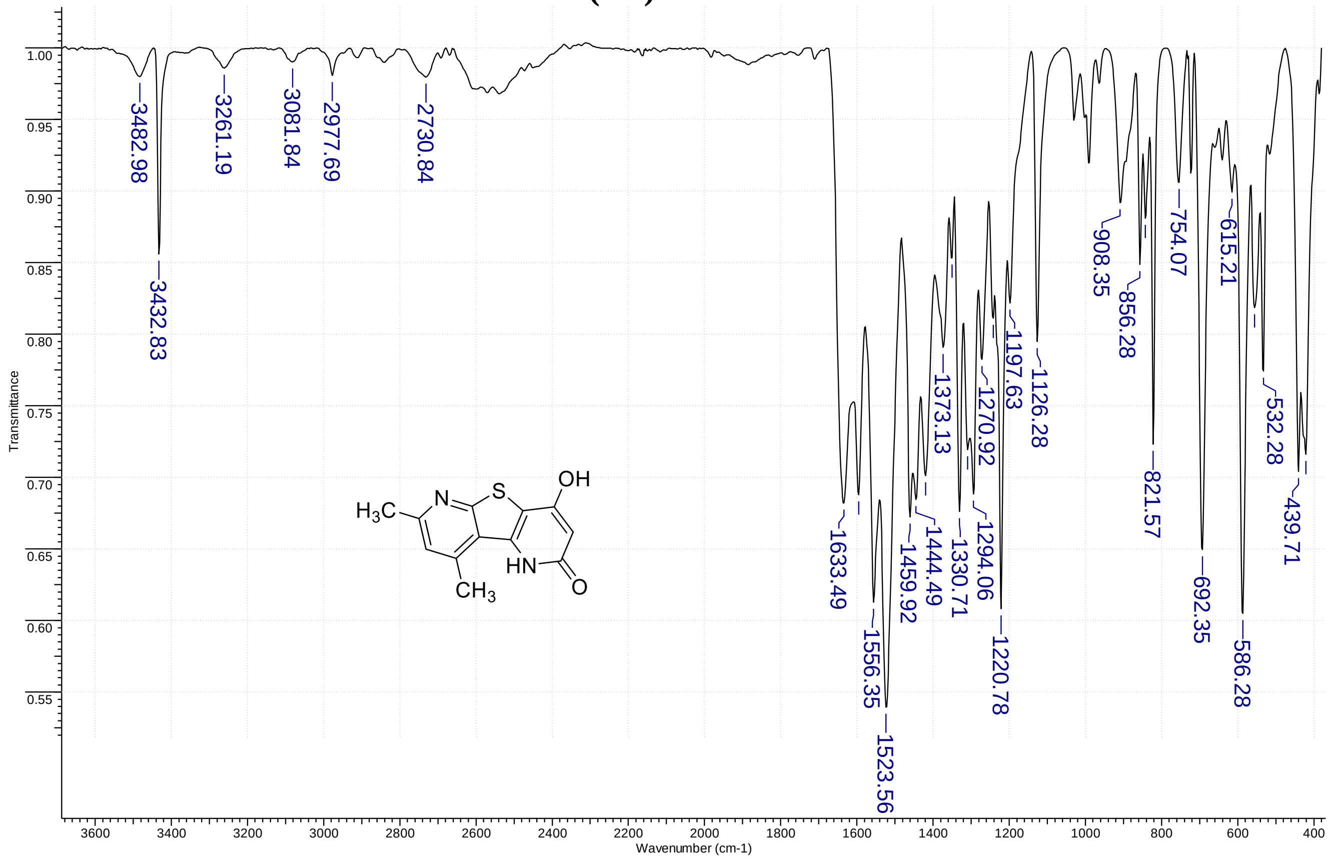

Supplement: Supplementary file 1 [file molecules-31-02975-s001.zip › molecules-4506043-supplementary.pdf]
